# Influence of pressure on a dysprosocenium single-molecule magnet†‡

**DOI:** 10.1039/d2cc06722f

**Published:** 2023-02-06

**Authors:** Vijay S. Parmar, Andreas M. Thiel, Rizwan Nabi, Gemma K. Gransbury, Marie S. Norre, Peter Evans, Sophie C. Corner, Jonathan M. Skelton, Nicholas F. Chilton, David P. Mills, Jacob Overgaard

**Affiliations:** a Department of Chemistry, Aarhus University Langelandsgade 140 Aarhus C DK-8000 Denmark jacobo@chem.au.dk; b Department of Chemistry, The University of Manchester Oxford Road Manchester M13 9PL UK Jonathan.skelton@manchester.ac.uk Nicholas.chilton@manchester.ac.uk David.mills@manchester.ac.uk

## Abstract

The effects of external pressure on a high-performing dysprosocenium single-molecule magnet are investigated using a combination of X-ray diffraction, magnetometry and theoretical calculations. The effective energy barrier (*U*_eff_) decreases from *ca.* 1300 cm^−1^ at ambient pressure to *ca.* 1125 cm^−1^ at 3 GPa. Our results indicate that compression < 1.2 GPa has a negligible effect on the Orbach process, but magnetic relaxation > 1 GPa increases *via* Raman relaxation and/or quantum tunnelling of magnetisation.

## Abstract 2

The effects of high-pressure on the structural and magnetic properties of the dysprosocenium cation are analyzed. Combined with periodic calculations, correlations between structural deformations and magnetic relaxation characteristics are obtained.

## Graphical abstract

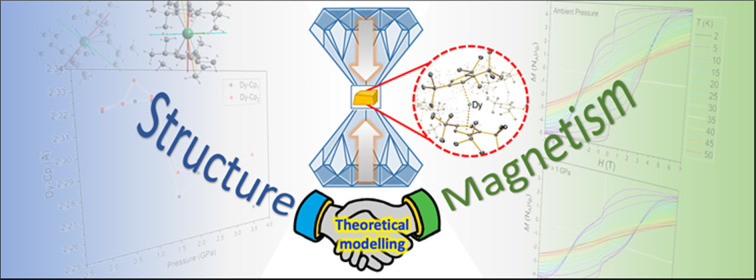

Single-molecule magnets (SMMs) have many potential uses as they can retain magnetic information,^1^ and lanthanide (Ln) SMMs have shown the most impressive properties to date.^2^ SMMs typically require liquid He cooling to show magnetic memory effects, yet highly axial Ln SMMs including dysprosocenium cations^3–7^ and their derivatives,^8–11^ a Tb^2+^ metallocene,^12^ and a Dy_2_ complex^13^ show memory effects close to the boiling point of liquid N_2_ (77 K), which is a far cheaper cryogen. To increase the effective barrier to magnetic reversal (*U*_eff_) the most magnetic *m*_J_ ±15/2 ground state of Dy^3+^, which has an oblate 4f electron density,^14^ is best-stabilized by an axial ligand field with no equatorially-bound ligands.^15,16^

*Ab initio* calculations^3^ and inelastic neutron scattering, magnetometry and solid-state NMR experiments^17,18^ on the dysprosocenium complex [Dy(Cp^ttt^)_2_][B(C_6_F_5_)_4_]·0.5CH_2_Cl_2_ (1·0.5CH_2_Cl_2_ = 1S) have shown that the local rigidity of substituted cyclopentadienyl ligands (Cp^R^) hinders Raman relaxation pathways, providing new design criteria for high-temperature SMMs. Shortening the Dy–Cp^R^ distances could significantly increase the crystal field strength, and computational studies have shown a correlation between *U*_eff_ and metal-ligand distances and bond angles.^3,15,19^

Recently, some of us probed the influence of pressure on the magnetic properties of d-block and Ln SMMs using high-pressure (HP) magnetometry, single-crystal XRD and *ab initio* calculations.^20,21^ For the 3d SMM [Co(SPh)_4_]^2−^, calculations using the geometries from experimental HP studies showed that compressing the molecule increases the axial zero-field splitting parameter, *D*, due to pressure-induced changes to the d-orbital energies.^21^ For a *pseudo*-pentagonal bipyramidal Dy^3+^ complex we found that the axial O-Dy-O angle decreased under pressure, resulting in a large drop in the magnetisation at zero field.^20^ These studies, together with those of others,^22^ demonstrate that HP crystallographic and magnetic experiments, coupled with *ab initio* calculations, can identify new magnetostructural correlations. Here we apply these methods to the dysprosocenium complex [Dy(Cp^ttt^)_2_][B(C_6_F_5_)_4_] (1), together with systematic magnetic decay measurements. This allows us to extract the magnetic relaxation times (*τ*) at various external pressures, providing clearer insights into the effect of pressure on individual magnetic relaxation pathways.

Due to a change in crystallisation conditions there is no lattice solvent in crystals of 1, unlike in the original report of 1S.^3^ Single crystal XRD data were collected at sample pressures from 0–3.52(5) GPa, and the structure of 1 (Fig. 1 and Table S1, ESI‡) was solved at each pressure point (see ESI‡ for details). At ambient pressure, the geometry of the cation in 1 is slightly different to that of 1S: the electrostatic contacts between Dy and the two C–H bonds of the ^*t*^Bu groups are at a narrower angle (C⋯Dy⋯C: 135.9(2)° in 1, *c.f*. 149.95(11)° in 1S), the Cp^ttt^_cent_⋯Dy⋯Cp^ttt^_cent_ angle is 153.66(6)° in 1 and 152.56(7)° in 1S. The decrease in unit cell volume with pressure is well-described using the 3rd order Birch–Murnaghan, Vinet and Murnaghan equations of state.^23–25^ The three models all give a similar bulk modulus of approximately 8 GPa, which is typical for molecular systems.^22,26^ Up to 1 GPa the intermolecular separation and the volume of crystalline void space decreases, with only minor changes to metrical parameters. Above 1 GPa intermolecular distances continue to decrease, but the geometry of the [Dy(Cp^ttt^)_2_]^+^ cation changes more significantly. Crystals of 1 are stable up to 3.52(5) GPa, beyond which they lose crystallinity.

**Fig. 1:**
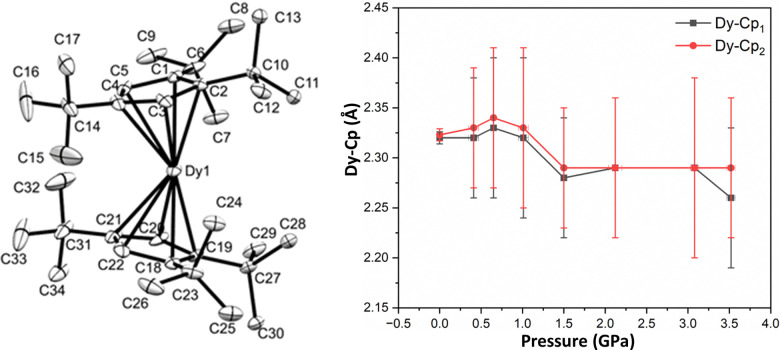
ORTEP drawing of the cation of 1 (left) at 0 GPa without H atoms. The ellipsoids are shown at 30% probability. The same labels are used for all pressure points. Distances between the Dy atom and the centroids of the two Cp^ttt^ rings in 1 with pressure (right).

Two types of intra-molecular distortions occur with increasing pressure: i) the Cp^ttt^ rings squeeze closer to the Dy^3+^ ion, with Dy⋯Cp^ttt^_cent_ distances decreasing from 2.32(1) Å to 2.26(7) and 2.29(7) Å (Fig. 1, *right*); and, ii) the co-planarity of the rings increases and their relative orientation becomes more staggered (Fig. S6 and S7, ESI‡).^3^ There is also a significant increase in the spread of Dy–C distances, indicating that Dy becomes less symmetrically placed relative to the Cp^ttt^ rings (Fig. S5, ESI‡). This distortion is quantified by the angles between the Dy–Cp^ttt^_cent_ vector and the Cp^ttt^ ring normals changing from 3–4° to 8–10° with increasing pressure (Fig. S9, ESI‡). We posit that the two changes have opposite effects on the magnetic properties. The partial “squeezing out” of Dy from the inter-Cp^ttt^ ring region should reduce the axiality of the system, whilst the increased interaction with Cp^ttt^ rings due to decreased Dy–Cp^ttt^_cent_ distance should increase axiality. Even at ambient pressure, the axiality of 1 (and 1S) is compromised by the presence of two methyl carbon atoms (C7 and C24) at distances of ∼2.93 Å from Dy (*c.f.* mean 2.964(7) Å in 1S)^3^ and nearly in the equatorial plane (∠Cp^ttt^_cent_-Dy–C: 82° and 108°). These distances decrease to ∼2.8 Å at 3.52(5) GPa (Fig. S10, ESI‡). We note that different relative orientations of Cp^ttt^ rings (staggered/eclipsed) were previously seen for a series of heavy [Ln(Cp^ttt^)_2_]^+^ cations.^3,27^

To examine how the electronic structure of 1 responds to pressure, we performed *ab initio* calculations using atomic coordinates extracted from the experimental XRD data (see ESI‡ for details). We calculated the energies of the eight Kramers doublets (KDs) of the *J* = 15/2 multiplet (Fig. S20 and Tables S6–S15, ESI‡). These data show that the ground state is stabilised relative to all other states, as seen by the increasing energy gaps between all KD states from ambient pressure to 0.41(6) GPa. The *m*_J_ compositions of the KDs do not change significantly, with all KDs up to and including the sixth being almost pure (> 97%). Magnetic relaxation *via* the Orbach mechanism may proceed through excited KDs where the *g*_z_ direction changes significantly relative to the ground state, or when the *g*_x_ or *g*_y_ values become significant (greater than *ca.* 0.1–0.2).^3^ At ambient pressure, relaxation is through the 5th or 6th KD, suggesting a *U*_eff_ of ∼1300 cm^−1^ (NEVPT2) in agreement with the experimental *U*_eff_ = 1240(20) cm^−1^ (see below). Pressurising has a negligible effect on the electronic structure from 0 to 1.01(6) GPa, but at 1.5 GPa relaxation *via* the 5th KD at 1250 cm^−1^, and at 3.08(5) GPa *via* 4th/5th KD at *ca.* 1125 cm^−1^ is suggested.

Magnetic measurements at ambient pressure were performed on 1 under the same conditions previously reported for 1S.^3^ The room temperature susceptibility value (*χ*_M_*T*_300K_) for 1 is 13.2 cm^3^ K mol^−1^ (Fig. S21, ESI‡), less than the expected free ion value of 14.2 cm^3^ K mol^−1^ for Dy(iii) but similar to the NEVPT2-calculated value of 13.5 cm^3^ K mol^−1^. The *χ*_M_*T* value drops slowly from 300 K down to 35 K due to the depopulation of excited Dy^3+^ crystal field states, followed by a rapid drop below 35 K consistent with magnetic blocking at low temperature. The saturation magnetic moment at 2 K is 4.92 *Nμ*_B_, close to the 5 *Nμ*_B_ expected for a pure *m*_J_ ± 15/2 ground state. At ambient pressure, 1 exhibits open hysteresis up to 66 K at a sweep rate of 21.96(1) Oe s^−1^ around zero field (Fig. 2 and Fig. S22, ESI‡). The shapes of the hysteresis loops are similar to those previously observed for 1S, which exhibits a slightly lower hysteresis temperature of 60 K.^3^ The coercive field at 2 K is 27 kOe and the remanent magnetisation is 79% of the saturated value, compared to 20–25 kOe and 83% for 1S.^3^ The wide hysteresis loops and similar remanent magnetisation at 2 K suggest similar quantum tunnelling of magnetization (QTM) rates in 1 and 1S. The hysteresis temperature of 1 is consistent with field-cooled (FC) and zero-field cooled (ZFC) magnetic susceptibility traces, which bifurcate from each other and from the scaled theoretical trace below 66 K (Fig. S23 and S24, ESI‡). The non-zero initial susceptibility measurement in the ZFC traces and the lower temperatures of the ZFC peaks (38 K at 500 Oe applied field and 42 K at 1 kOe) are consistent with an imperfect zero-field condition.^28^ The ZFC peaks in 1 are in good agreement with the ZFC peak at 38 K in 1S (500 Oe and 1 kOe).^3^

**Fig. 2:**
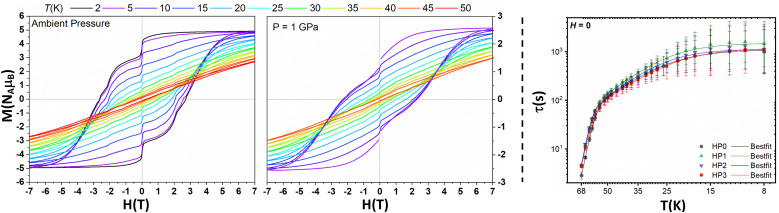
Hysteresis loops of 1 at 2 K and 5–50 K in 5 K steps recorded at ambient pressure (left) and at 1 GPa (centre). For the series at 1 GPa, data below +/-800 Oe at 5 K are omitted due to the superconductive manometer (Pb). *Right*: Relaxation profile (*τ vs*. *T*) for 1 at ambient pressure (HP0), 0.37 GPa (HP1), 0.79 GPa (HP2) and 1.2 GPa (HP3) (see Fig. S41 and S42, ESI‡ for fitted data and variations in the distribution of *τ* (*β*) with temperature).

Direct current (DC) magnetisation decay and alternating current (AC) magnetic susceptibility measurements were respectively used to extract *τ* values for 1 under ambient pressure at 5–57 K and 70–107 K (Tables S16 and S17 and Fig. S25–S29, ESI‡). The relaxation rate profile of 1 shows three clear regimes: Orbach relaxation at high temperature (*T* > 60 K), QTM at low temperature (*T* < 10 K), and Raman relaxation at intermediate temperatures (Fig. S31, ESI‡). The relaxation decay profile of 1 is in good agreement with the data previously reported for 1S^3^ (Fig. S30, ESI‡). The 100 s blocking temperatures for 1 (52 K) and 1S (53 K) are similar. Fitting the entire relaxation profile with CC-FIT2^29^ (Fig. S31, ESI‡) reveals the listed relaxation parameters in Table 1. The Orbach parameters are within error of the values reported for 1S^3^ and suggest relaxation for 1*via* the 5th (1112/1193 cm^−1^, CASSCF/NEVPT2) or the 6th KD (1271/1385 cm^−1^). The Raman regimes for 1 and 1S are equivalent as the relaxation profiles overlay (Fig. S30, ESI‡), despite them being fitted with different parameters (*c.f. C* = 1.664 × 10^−6^ s^−1^ K^−*n*^ and *n* = 2.151 for 1S). We attribute the differences in these parameters to the increased amount of low-temperature data used in this study to fit the QTM rate, the simultaneous fitting of the entire temperature range, and to the weighting of the experimental data points using estimated standard deviations.^29^

Magnetic data was collected on 1 under multiple pressures as a function of temperature (see ESI‡ for details). The hysteresis loops remain open until at least 50 K at 1 GPa and are similar to the ambient pressure data at the highest temperatures where the Orbach mechanism is predominant (Fig. 2). However, the data recorded at 1 GPa shows a greater loss of magnetisation as the field approaches zero at low temperatures, suggesting enhanced QTM and/or Raman relaxation rates under pressure. To check this assumption, magnetisation decay measurements in the range of 8–68 K were performed at four pressures, *viz.* ambient (HP0), 0.37 GPa (HP1), 0.79 GPa (HP2) and 1.2 GPa (HP3) (Fig. S35–S38, ESI‡).

**Table 1:** Magnetic relaxation parameters for 1 at various pressures; the estimated standard deviations (±) are based on the ESDs of *τ* shown in Fig. 2 (right)

Pressure/GPa	*U*_eff_/K	*a* {*τ*_0_ = 10^−a^/s}	*c* {*C* =10^−*c*^/s^−1^K^−*n*^}	*n*	*b* {*τ*_QTM_ = 10^*b*^/s}
Ambient without DAC	1787 ± 28	10.80 ± 0.14	8.22 ± 1.05	3.61 ± 0.61	3.06 ± 0.16
Ambient (HP0)	1355 ± 123	8.18 ± 0.82	7.30 ± 1.28	3.05 ± 0.76	3.04 ± 0.22
0.37 (HP1)	1473 ± 68	8.69 ± 0.44	7.80 ± 0.77	3.31 ± 0.45	3.18 ± 0.19
0.79 (HP2)	1578 ± 129	9.36 ± 0.84	7.87 ± 1.00	3.38 ± 0.58	3.05 ± 0.20
1.2 (HP3)	1386 ± 151	8.16 ± 0.97	7.05 ± 1.26	2.92 ± 0.74	3.07 ± 0.28

The magnetisation decays were fitted to extract *τ* as a function of temperature (Fig. 2 and Tables S19–S22, ESI‡) from which the relaxation parameters were obtained *via* fitting (Fig. 2 and Fig. S40, ESI‡ and Table 1). We note the discrepancy in the magnetic relaxation parameters at ambient pressure without the high-pressure cell (DAC); this is due to the larger temperature range used in fitting the HP0-without DAC data, as the *τ* values at HP0 with and without DAC overlap (Fig. S39, ESI‡). Owing to the fast relaxation rates in the Orbach region, which are at the limit of the instrumental capabilities for decay measurements, the rates and therefore Orbach parameters should be treated with caution. Upon pressurising from ambient to 0.37 GPa we observe a decrease in both Raman and QTM rates, but relaxation in these regions then appears to become faster upon a further increase of pressure to 0.79 and 1.2 GPa (Fig. 2), in agreement with the hysteresis data.

Magnetic relaxation in SMMs arises due to spin-phonon coupling, where vibrational quanta are absorbed, emitted or scattered by the molecule. The phonon density of states (pDOS) thus plays a crucial role in the spin dynamics of such systems.^17^ To get insight into the pDOS we performed periodic density-functional theory (DFT) calculations on each of the XRD structures (see ESI‡). We find that from ambient pressure to 0.41 GPa there is a significant decrease in intensity of features in the low-energy pDOS around ∼75 (A) and ∼225 cm^−1^ (B), whilst the intensity of peaks around ∼275 (C) and 350 cm^−1^ (D) increases (Fig. 3 and Fig. S12–S19, ESI‡). Upon further increase in pressure there is a gradual and uniform shift of the pDOS to higher energies, with the exception of an abrupt decrease in the pDOS at ∼275 cm^−1^ (C) between 0.41 and 0.65 GPa, and no further changes in the peak at ∼350 cm^−1^ (D). As the pressure-dependent relaxation measurements indicate the largest changes to the Raman region rather than the Orbach region, we concentrate mainly on the lowest-energy region below 200 cm^−1^ where the modes will have significant populations at low temperature. Given the abrupt slowing of magnetic relaxation observed in the 0.37 GPa dataset, we suggest that this could be due to the abrupt reduction in the pDOS at ∼75 cm^−1^ (A). The progression to faster relaxation at 0.79 and 1.2 GPa could conceivably arise from the shift in the pDOS to higher energies. However, considering phonon occupation alone this would suggest a reduction in relaxation rates contrary to the experimental observations. There are thus perhaps counterbalancing effects at play beyond phonon occupation, such as changes in the spin-phonon coupling of the low-energy modes that lead to the observed changes in magnetic relaxation rates.

**Fig. 3:**
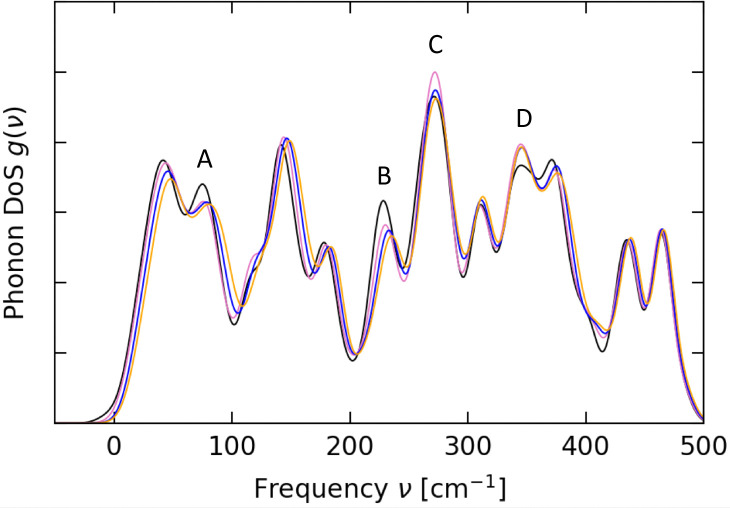
Low-energy pDOS calculated for 1 at 0 GPa (black), 0.41 GPa (pink), 0.65 GPa (blue) and 1.01 GPa (orange).

In summary, we have shown significant geometrical changes to a dysprosocenium cation using high-pressure single crystal X-ray diffraction. Pressurisation leads to loss of intermolecular void space, linear compression of the individual sandwich-like molecules, and movement of Dy away from the molecular centre. Periodic DFT shows that this results in a slight upshift of the low-energy pDOS, which would correlate with slower relaxation in the Raman regime. However, experimental hysteresis loops at 1 GPa suggest that relaxation is enhanced, and thus the subtle change to the pDOS is likely not the only effect, and pressurisation also impacts QTM. *Ab initio* calculations show that the combination of intramolecular changes leads to a reduced energy barrier to relaxation, suggesting that the relaxation at 3.08(5) GPa involves the 4th or 5th Kramers doublet, instead of the 5th or 6th KD at ambient pressure.

HP magnetometry shows a negligible impact of pressure on the Orbach process, whereas the Raman and QTM processes are suppressed at lower pressure but return to ambient levels at pressures around 1 GPa and above. Extrapolating the changes in magnetisation decay following pressure changes from 0.37 to 1.2 GPa suggests that the faster Raman and QTM processes are likely to dominate when pressures exceed 1.2 GPa. In conclusion, this work presents a proof of concept for mechanistically exploring the magnetic dynamics of SMMs under pressure, and could be utilised for unravelling molecular and bulk structure-property relationships.

This work was supported by VILLUM FONDEN (12391), the UK EPSRC (EP/R002605X/1), the European Research Council (CoG-816268, StG-851504), the Royal Society (URF for N.F.C., URF191320), UKRI (FLF for J.M.S., MR/T043121/1), and ESS-Lighthouse SMART. We thank the Centres for Integrated Materials Research (iMAT) and Scientific Computing (https://phys.au.dk/forskning/cscaa) at Aarhus University, the EPSRC National Electron Paramagnetic Resonance Service for access to the SQUID magnetometer and the University of Manchester for access to the Computational Shared Facility. *Via* our membership of the UK's HEC Materials Chemistry Consortium (funded by EPSRC EP/R029431), we used the ARCHER2 UK National Supercomputing Service.

## Conflicts of interest

There are no conflicts to declare.

## Supplementary Material

CC-059-D2CC06722F-s001

CC-059-D2CC06722F-s002
