# Supplementary material for: Influence of pressure on a dysprosocenium single-molecule magnet
Source: Chem Commun (Camb). 2023 Feb 6;59(18):2656–9. doi: 10.1039/d2cc06722f (PMC9972519; doi:10.1039/d2cc06722f)
Supplement: CC-059-D2CC06722F-s001 [file CC-059-D2CC06722F-s001.pdf]

This journal is © The Royal Society of Chemistry 2023

Influence of pressure on a dysprosocenium single-molecule magnet

Vijay S. Parmar, [a] Andreas M. Thiel,[a] Rizwan Nabi,[b] Gemma K. Gransbury,[b] Marie S. Norre,[a] Peter Evans,[b] Sophie C. Corner,[b] Jonathan M. Skelton,*[b] Nicholas F. Chilton,*[b] David P. Mills,*[b] and Jacob Overgaard*[a]

[a] Department of Chemistry, Aarhus University, Langelandsgade 140, DK-8000 Aarhus C, Denmark.
[b] Department of Chemistry, The University of Manchester, Oxford Road, Manchester, M13 9PL, UK.

Contents

1. Synthetic details....S2
2. Crystallographic details....S3
3. Periodic DFT calculations....S19
4. CASSCF calculations....S23
5. Ambient pressure magnetic measurements....S30
6. High pressure magnetic measurements:....S42
7. References....S68

1. Synthetic details

General procedures:

All manipulations were performed under an atmosphere of argon with rigorous exclusion of oxygen and water using Schlenk line and glove box techniques. $[Dy(Cp^{ttt})_2Cl]$[1–3] (Cp^{ttt}=1,2,4-tri(*tert*-butyl)cyclopentadienyl) and $[(Et_3Si)_2H][B(C_6F_5)_4]$[4,5] were prepared according to published procedures. Benzene was dried over potassium and fluorobenzene was dried over CaH_2 by heating the solvents and drying agents under reflux for >18 hours and then distilling. *n*-hexane was dried by passing over an alumina column. Solvents were stored over K mirrors (benzene, *n*-hexane) or 4 Å molecular sieves (fluorobenzene) and degassed before use. The identity of the product was confirmed by elemental analysis and ATR-IR spectroscopy carried out on a powdered sample.

Synthesis of $[Dy(Cp^{ttt})_2][B(C_6F_5)_4]$ (**1**):

1 was obtained using an adaptation of the published procedure for the synthesis of **1**·CH_2Cl_2 (**1S**[1,2]): Benzene (15 ml) was added to a mixture of $[Dy(Cp^{ttt})_2Cl]$ (0.5054 g, 0.76 mmol) and $[(Et_3Si)_2H][B(C_6F_5)_4]$ (0.6923 g, 0.76 g). The resulting yellow mixture was stirred overnight to give a suspension of a yellow solid in a colourless solution. The solvent was removed *in vacuo* and the product was dissolved in fluorobenzene (5 ml). The resulting yellow solution was filtered to remove a small amount of pale solid and reduced in volume to 1 ml before being layered with *n*-hexane (15 ml). Large yellow crystals of **1** were formed after standing the mixture for 1 week. The supernatant solution was decanted off and the crystals dried under vacuum (0.5538 g, 56%). Anal. calc. for $C_{58}H_{58}BDyF_{20}$ (%): C, 53.24; H, 4.47. Found: C, 52.78; H, 4.48. FTIR (ATR, microcrystalline; $\tilde{v}$ / cm^{-1}): 3016 (vw), 2965 (w), 2947 (w), 2907 (w), 2870 (w), 2781 (w), 1643 (w), 1512 (st), 1460 (st), 1410 (v w), 1400 (w), 1367 (m), 1340 (v w), 1276 (m), 1238 (m), 1198 (w), 1162 (w), 1082 (st), 1034 (v w), 1025 (w), 975 (st), 923 (w), 908 (w), 850 (m), 830 (v w), 809 (w), 775 (m), 756 (m), 725 (w), 706 (w), 683 (m), 662 (m), 639 (v w), 612 (m), 601 (w), 573 (m), 549 (w), 532 (v w), 515 (v w), 497 (v w), 472 (w), 439 (w), 417 (vw).

2. Crystallographic details

High-pressure structures were measured at room temperature in an Almax plate DAC with diamonds with a 600 µm diameter culet and an approximate opening angle of 40°. Steel gaskets were used with an indentation thickness of approximately 150 µm and a gasket hole diameter of 250-300 µm for both crystals. The gasket hole was drilled with a Hylozoic Products Micro Electric Discharge Machining System (EDM). In this study the Fomblin Y and Daphne 7373 oils were used as pressure transmitting media (PTM). Daphne 7373 is a common PTM in the SMM community, mostly because it is also used in HP cells for magnetic measurements, despite it being hydrostatic only up to 2.2 GPa.[6] It can, however, be used above the hydrostatic limit. Fomblin Y is not a common PTM and its hydrostaticity is unknown, but it was selected as the crystals were stored in this oil and there was no uncertainty about their stability in it. It was observed that Fomblin Y does not mix well with Paratone-N oil and it is very susceptible to forming and stabilizing air bubbles, which made it difficult to close the DAC. Despite this, it was used due to concerns about the stability of the crystals in other media. We later found that the decomposition observed when closing the DAC with Daphne 7373 was due to the screening of crystals beforehand and not the specific use of this PTM.

In order to limit the time that the crystals were exposed to air, the DAC was pre-packed with the gasket, ruby and PTM such that the crystals could be transferred straight from the Paratone-N oil into the PTM.

A 50×190×190 µm yellowish-transparent crystal was placed in the DAC with Fomblin Y as the pressure transmitting medium. Because of the sensitivity of the sample, the crystal was not screened beforehand. A picture of this crystal inside the DAC can be seen in Figure S2, and all crystallographic details are given in Table S1.

All crystallographic measurements were performed on a Rigaku Oxford Diffraction SuperNova equipped with an Atlas CCD detector and a molybdenum X-ray source. Data was integrated using CrysalisPRO, while the structure was solved using SHELXT and refined using SHELXL. Ambient pressure data was measured on a 50×50×100 µm crystal of compound **1** in Paratone-N oil on a nylon loop at 100 K. The crystal structure is shown in Figure S1.

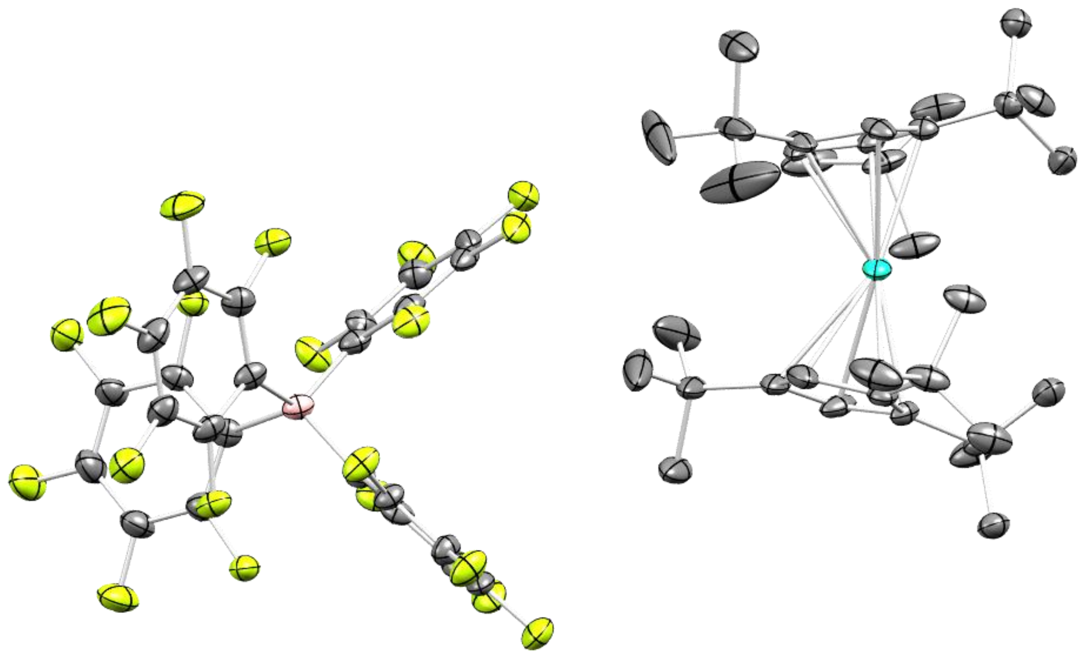

Figure S1. ORTEP drawing of the asymmetric unit of **1** at ambient pressure at 100 K. Hydrogen atoms have been omitted for clarity. Thermal ellipsoids are shown at 50% probability. Atom colours: C - grey; Dy - turquoise; F - yellow; B - pink.

With HP data 3D peak finding was used to find the highest number of correct peaks. During data reduction, the recommended settings for HP data were used. The opening angle used for the data reduction to avoid including reflections lying in the gasket shadow was 38°, which was a compromise between avoiding the gasket shadow and losing completeness. For background evaluation a range of 5° and a repeat frequency of 10° was used, and for background integration a smart background with a frame range of 3° was used.

A resolution limit of 1 Å was used and all frames with a R_{int} value above 0.6 were rejected. In order to achieve chemically sensible structures of compound **1** from the high-pressure data, several restraints were applied during the structure refinement. Both the cyclopentadienyl and the C_6F_5 rings were restrained to be regular pentagons and hexagons, respectively, and the C_6F_5 rings were restrained to be flat using the FLAT restraint. All reflections with a negative intensity were omitted while all reflections with an error/esd > 3 were manually checked and most of them omitted. Most of these reflections were either in the gasket shadow or were overlapped by diamond peaks or gasket rings.

All atoms except Dy were refined isotropically, with the exception of the HP3 dataset for which the data quality was poor and all atoms had to be refined isotropically. Furthermore, it was necessary to use the AFIX33 restraint several structures and sometimes on several C atoms. Specifically, AFIX33 was used on C15 in the HP3 dataset, C8 and C12 in HP6A, and C34 in HP7A. The HP8 dataset was poorer and several restraints were needed, namely AFIX33 on C7, C13, C15, C17, C30, C32 and C33 and SADI on the *tert*-Bu groups. The Cp rings are restrained and the C-C bond distances are fixed to 1.42 Å.

The pressure in the DAC was determined using an in-house setup with a green laser. The measured fluorescence spectrum at each pressure point was analyzed by a homemade Matlab script. The pressure was measured both before and after the diffraction measurements and the average taken to be the pressure during the data collection. The standard deviation on each measured pressure was also calculated, but as the pressure fluctuation during the experiments was larger than this the difference between the start and end pressure was taken as the uncertainty instead.

Due to the ambient pressure dataset having been measured at 100 K, there was no ambient pressure volume (V_0) available for the EoS fitting. Having a measure of V_0 was found to be necessary, and so as a substitute the entire pressure range was shifted by -0.41 GPa, effectively making the first pressure point the ambient one, the EoS fits performed on the adjusted range, and the pressure range shifted back after fitting. This leads to some uncertainty in the estimated V_0.

Due to pressure fluctuations the pressure of HP4 was estimated based on the unit-cell volume and the EoS fitted to 1.5 GPa, and thus was not part of the EoS fit.

Table S1. Crystallographic data for the eight datasets collected in this work. * Pressure estimated based on the unit-cell volume and fitted EoS.

Dataset name	CHP0	CHP1	CHP2	CHP3	CHP4	CHP5	CHP6	CHP7
Pressure (GPa)	0	0.41(6)	0.65(2)	1.01(6)	1.5*	2.12(8)	3.08(5)	3.52(5)
T (K)	100	295	295	295	295	295	295	295
Z	2	2	2	2	2	2	2	2
Space group	*P*-1	*P*-1	*P*-1	*P*-1	*P*-1	*P*-1	*P*-1	*P*-1
a (Å)	11.6278(3)	11.6573(8)	11.6015(8)	11.5239(18)	11.429(5)	11.355(2)	11.290(2)	11.287(3)
b (Å)	12.6173(5)	12.4230(9)	12.2877(10)	12.176(2)	12.062(6)	12.014(3)	11.883(3)	11.804(4)
c (Å)	20.3029(7)	19.898(3)	19.663(4)	19.539(8)	19.312(19)	19.033(13)	18.809(10)	18.747(14)
α (°)	107.031(3)	106.869(11)	106.861(12)	106.90(3)	106.754(18)	107.39(4)	107.49(4)	107.427(5)
β (°)	96.387(3)	95.482(10)	95.221(11)	95.05(3)	95.037(17)	95.26(4)	95.15(3)	95.31(4)
γ (°)	99.377(3)	100.196(6)	100.515(7)	100.716(14)	100.632(10)	100.76(2)	100.624(17)	100.58(3)
V (Å^3)	2769.8(2)	2680.8(5)	2606.6(6)	2548(1)	2477(2)	2404(2)	2337(2)	2316(2)
Resolution (Å)	0.83	1	1	1	1	1	1	1
Completeness (%)	100	39	38	37	39	38	39	39
Average redundancy	2.6	10.1	8.6	6.2	-	8.2	8.5	7
Average I/σ	16.2	12.9	9.2	5.9	16.8	6.3	6.5	5.3
R1 (%)	4.02	8.06	8.61	12.43	10.90	14.00	14.14	15.24
$wR2$ (%)	9.44	21.15	23.97	33.22	37.64	36.79	36.88	39.67
R_{int} (%)	5.31	12.2	16.22	21.60	8.59	22.26	20.94	24.22
GooF	1.045	1.052	1.083	1.145	1.786	1.242	1.226	1.188
$\Delta\rho_{min, max}$ (e Å^{-3})	-0.8, 1.2	-0.7, 0.5	-1.0, 0.7	-1.2, 1.2	-1.9, 1.9	-1.3, 0.8	-1.2, 0.6	-1.0, 0.6

CCDC 2046705–2046709 & 2046711–20467 contains the supplementary crystallographic data of the high-pressure series of **1**. These data can be obtained free of charge via www.ccdc.cam.ac.uk/data_request/cif.

Table S2. Structural parameters of interest from the HP series on **1**. The uncertainties on the Cp-Dy-Cp, torsion angle, Cp-Cp angle and Dy distortion from the $Cp_{1,2}$ normal were set as the C7-Dy-C24 uncertainty. The uncertainties on the Dy-$Cp_{1,2}$ were set as the Dy-C24 uncertainty. * Pressure estimated based on the unit-cell volume and fitted EoS.

Dataset name	CHP0	CHP1	CHP2	CHP3	CHP4	CHP5	CHP6	CHP7
Pressure (GPa)	0	0.41(6)	0.65(2)	1.01(6)	1.5*	2.12(8)	3.08(5)	3.52(5)
T (K)	100	295	295	295	295	295	295	295
Dy-C1 (Å)	2.562(4)	2.55(1)	2.54(2)	2.54(2)	2.45(2)	2.45(2)	2.43(2)	2.45(3)
Dy-C2 (Å)	2.596(4)	2.61(2)	2.61(2)	2.61(2)	2.54(2)	2.56(3)	2.56(3)	2.54(4)
Dy-C3 (Å)	2.641(4)	2.68(1)	2.69(2)	2.69(2)	2.64(2)	2.70(3)	2.72(3)	2.66(3)
Dy-C4 (Å)	2.684(4)	2.67(2)	2.67(2)	2.67(3)	2.65(2)	2.68(4)	2.69(4)	2.64(4)
Dy-C5 (Å)	2.592(4)	2.59(2)	2.58(2)	2.58(3)	2.56(2)	2.53(4)	2.52(4)	2.51(4)
Dy-C18 (Å)	2.554(4)	2.54(2)	2.55(2)	2.54(2)	2.51(2)	2.46(2)	2.43(3)	2.51(3)
Dy-C19 (Å)	2.579(4)	2.57(2)	2.58(2)	2.57(3)	2.51(2)	2.49(4)	2.45(4)	2.46(3)
Dy-C20 (Å)	2.644(4)	2.68(1)	2.68(2)	2.67(2)	2.61(2)	2.65(2)	2.66(3)	2.59(3)
Dy-C21 (Å)	2.699(4)	2.70(2)	2.71(2)	2.70(3)	2.67(3)	2.72(4)	2.76(5)	2.71(4)
Dy-C22 (Å)	2.606(4)	2.61(2)	2.63(2)	2.63(3)	2.62(2)	2.61(3)	2.63(4)	2.66(3)
Dy-Cp_1 (Å)	2.320(6)	2.32(6)	2.33(7)	2.32(8)	2.28(6)	2.29(7)	2.29(9)	2.26(7)
Dy-Cp_2 (Å)	2.323(6)	2.33(6)	2.34(7)	2.33(8)	2.29(6)	2.29(7)	2.29(9)	2.29(7)
Cp_1-Dy-Cp_2 (°)	153.45(8)	155.7(7)	155.5(9)	156(1)	156.3(7)	155(2)	154(2)	153(1)
Torsion angle (C4-$Cp_{1,cent}$-$Cp_{2,cent}$-C21) (°)	58.31(8)	62.1(7)	62.2(9)	66(1)	60.7(7)	69(2)	62(2)	67(1)
Cp-Cp angle (°)	21.80(8)	20.5(7)	20.8(9)	21(1)	17.2(7)	19(2)	17(2)	20(1)
Dy-C7 (Å)	2.939(5)	2.97(2)	2.97(2)	2.96(3)	2.97(2)	2.96(4)	2.85(5)	2.86(4)
Dy-C24 (Å)	2.911(6)	2.93(6)	2.87(7)	2.86(8)	2.89(6)	2.85(7)	2.76(9)	2.73(7)
Dy distortion from Cp_1 normal (°)	3.18(8)	3.7(7)	4.3(9)	4(1)	4.6(7)	7(2)	8(2)	6(1)
Dy distortion from Cp_2 normal (°)	3.93(8)	4.6(7)	4.5(9)	4(1)	4.8(7)	8(2)	10(2)	7(1)

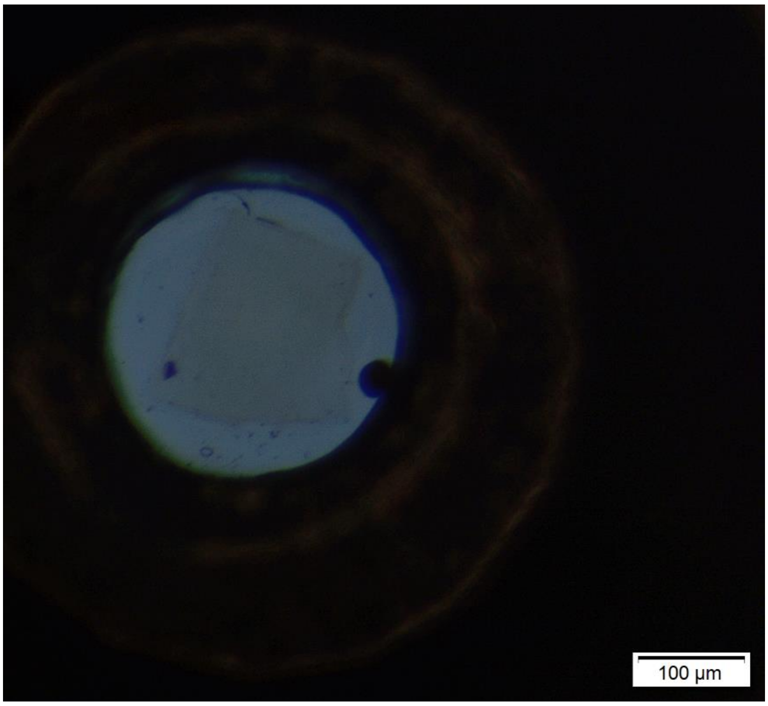

Figure S2. Picture of a crystal of **1** inside the gasket hole in a closed DAC. The ruby crystal is visible to the right of the hole.

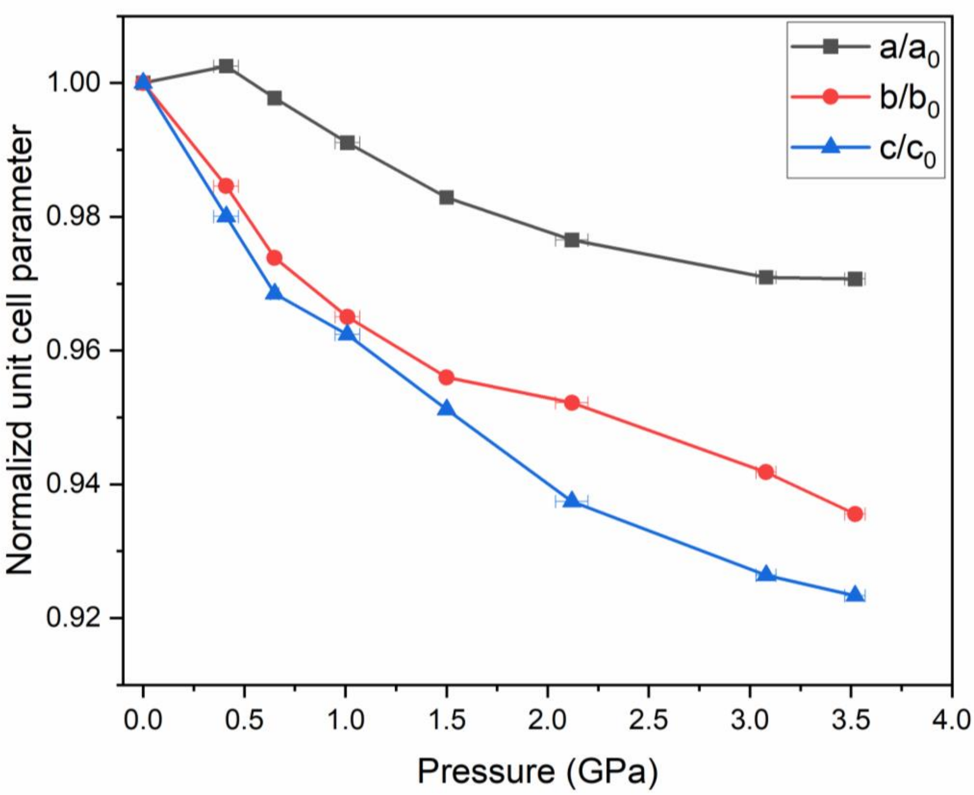

Figure S3. Normalized unit-cell parameters as a function of pressure: a/a_0 (black), b/b_0 (red) and c/c_0 (blue). The compressibility of one of the lattice parameters more than the others depends on the crystal packing; more compact packing and stronger intermolecular interaction along one direction can reduce the compressibility in that direction (see Figure S11 and Table S4).

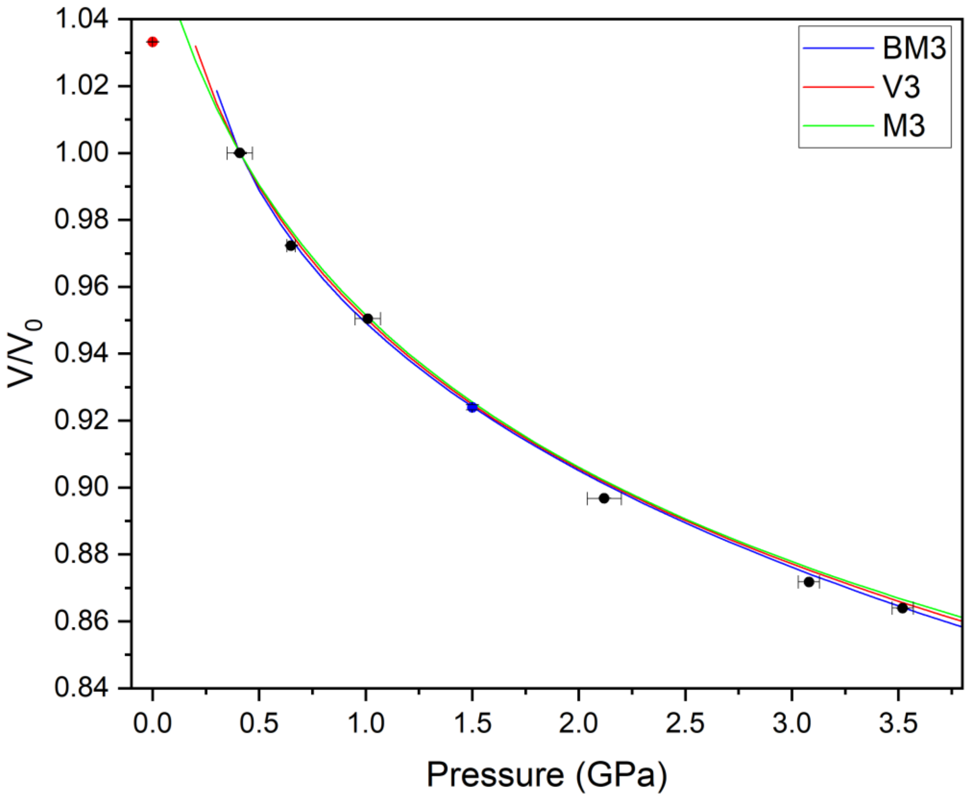

Figure S4. Normalized unit-cell volume of **1** as a function of pressure. Three EoS fits are overlaid as lines: V3 - 3rd-order Vinet (red); BM3 - 3rd-order Birch-Murnaghan (blue); and M3 - 3rd-order Murnaghan (green). Error bars are included on both the volume and pressure axes. The red and blue data points show the HP0 (ambient) volume collected at 100 K and the HP4 volume collected at 1.5 GPa (estimated), both of which were excluded from the fit as explained in the text.

Table S3. Parameters for the EoS fits on **1**. BM3 - 3rd-order Birch-Murnaghan; V3 - 3rd-order Vinet; M3 - 3rd-order Murnaghan. These fits were carried out without an ambient volume, as explained in the text.

Method	BM3	V3	M3
χ^2	0.73	0.67	0.68
V_0 (Å^3)	2677(17)	2673(12)	2671(11)
K_0 (GPa)	7(3)	8.3(18)	8.9(16)
K'	20(9)	14(2)	11.0(16)
K'' (GPa^{-1})	-37.022	-6.3328	-

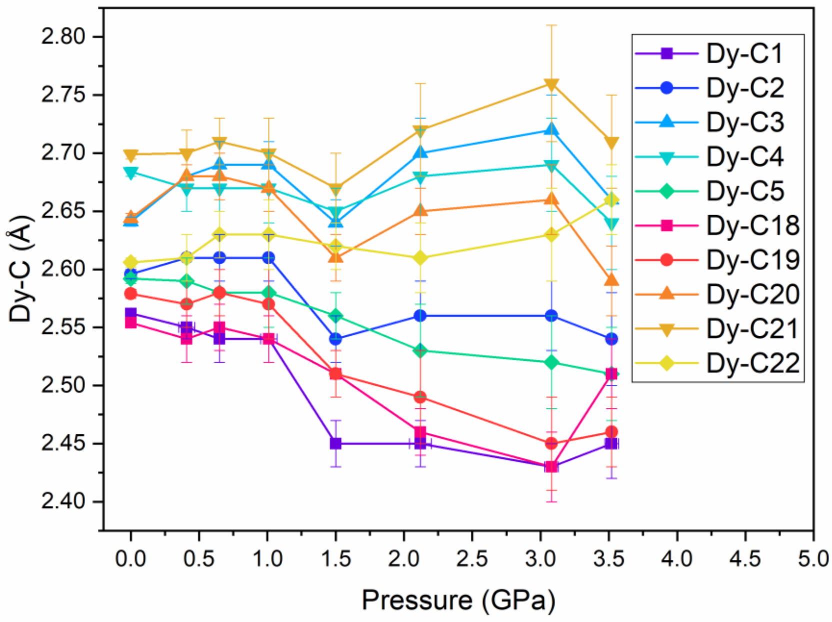

Figure S5. Distances between the Dy cation and carbon atoms in the two Cp rings as a function of pressure.

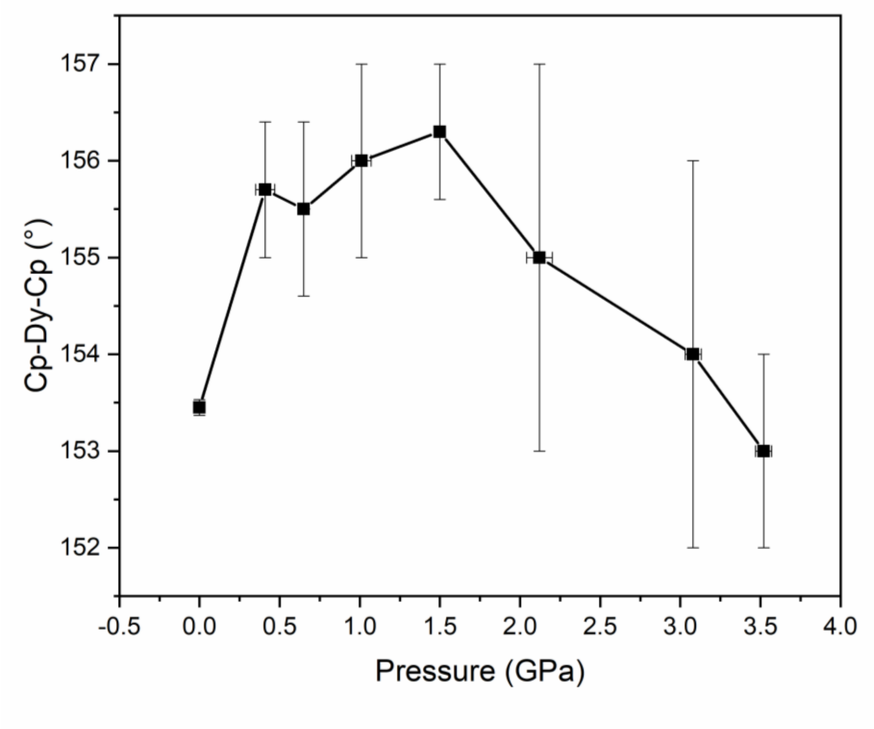

Figure S6. Angle between the Dy cation and the two Cp ring centroids as a function of pressure.

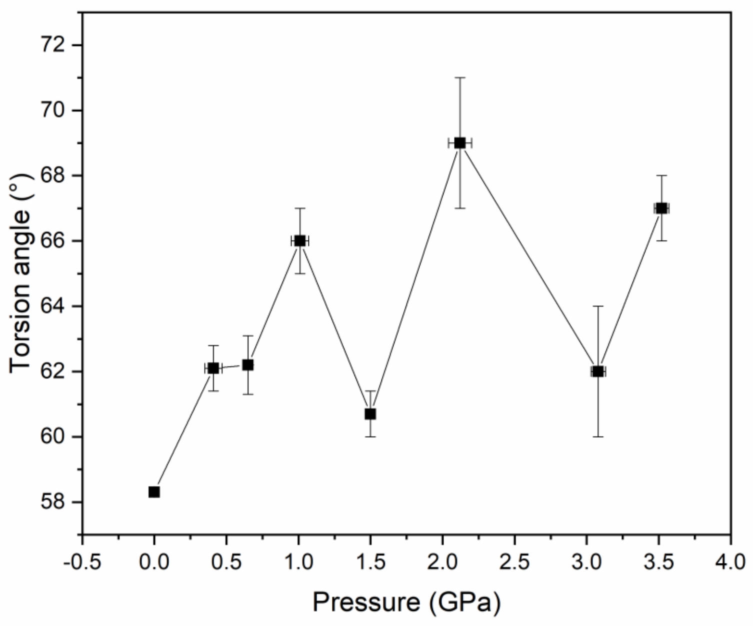

Figure S7. Torsion angle between the two Cp rings, defined as the torsion angle between C4-$Cp_{1,cent}$-$Cp_{2,cent}$-C21, as a function of pressure.

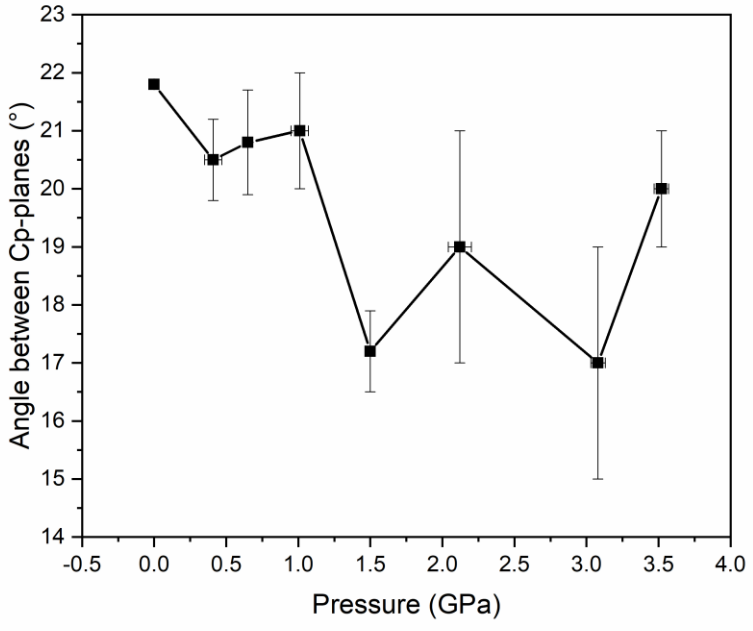

Figure S8. Cp-Cp angle between the planes defined by the two Cp rings as a function of pressure.

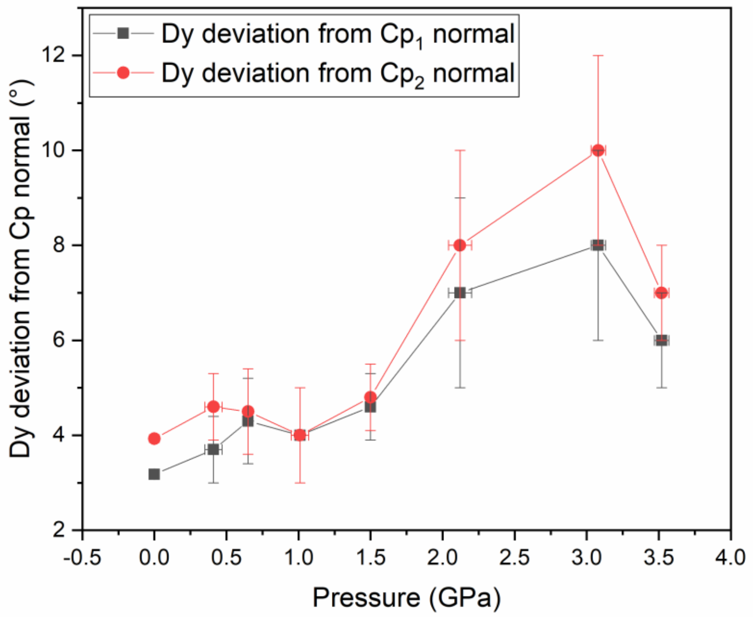

Figure S9. Angle between the Dy-Cp_{cent} and Cp ring normal as a function of pressure.

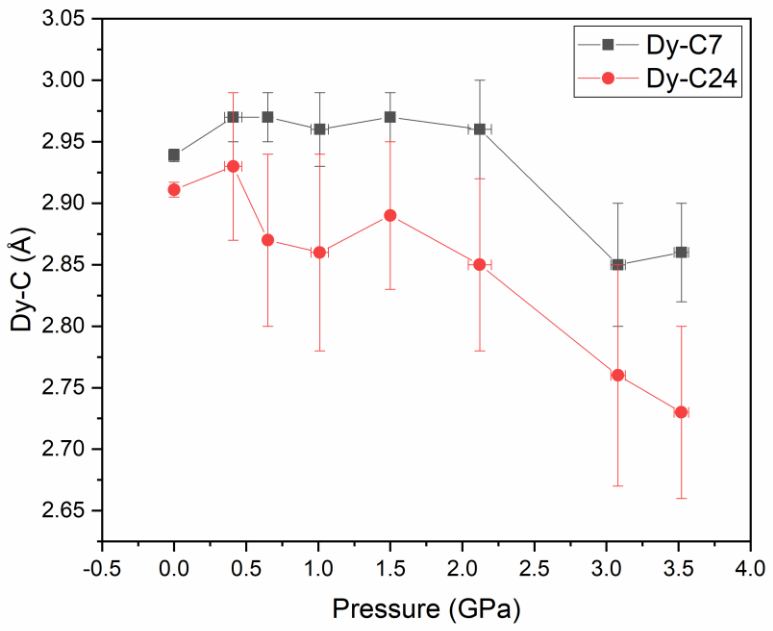

Figure S10. Distance between the Dy cation and the two closest methyl groups as a function of pressure (C7 - black and C24 - red).

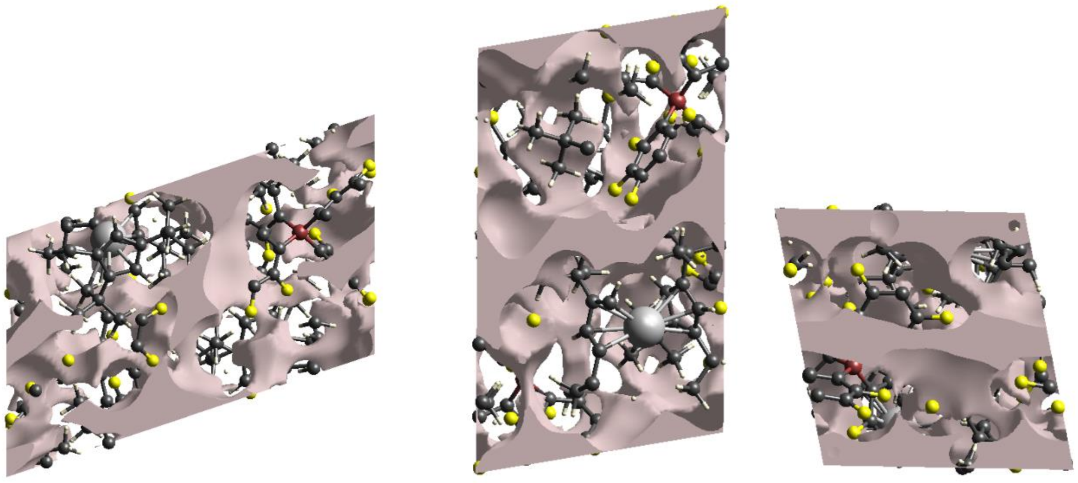

CHP0

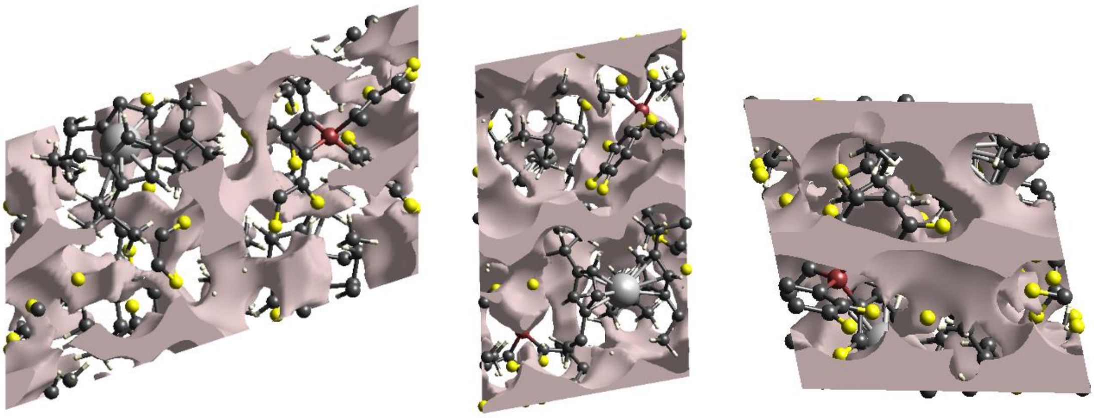

CHP1

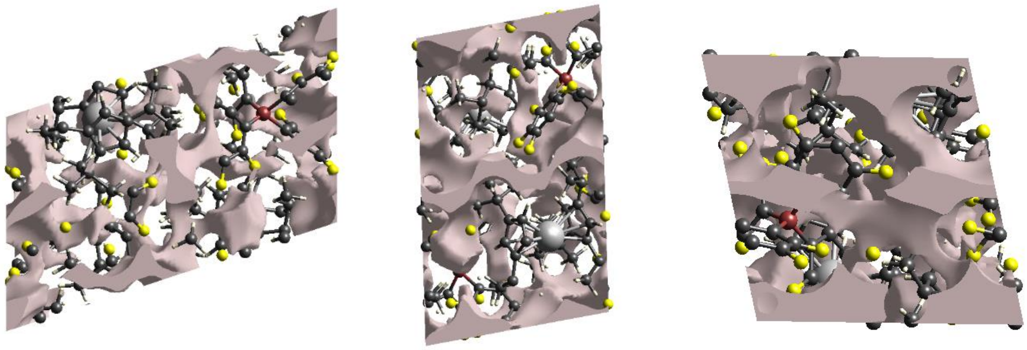

CHP2

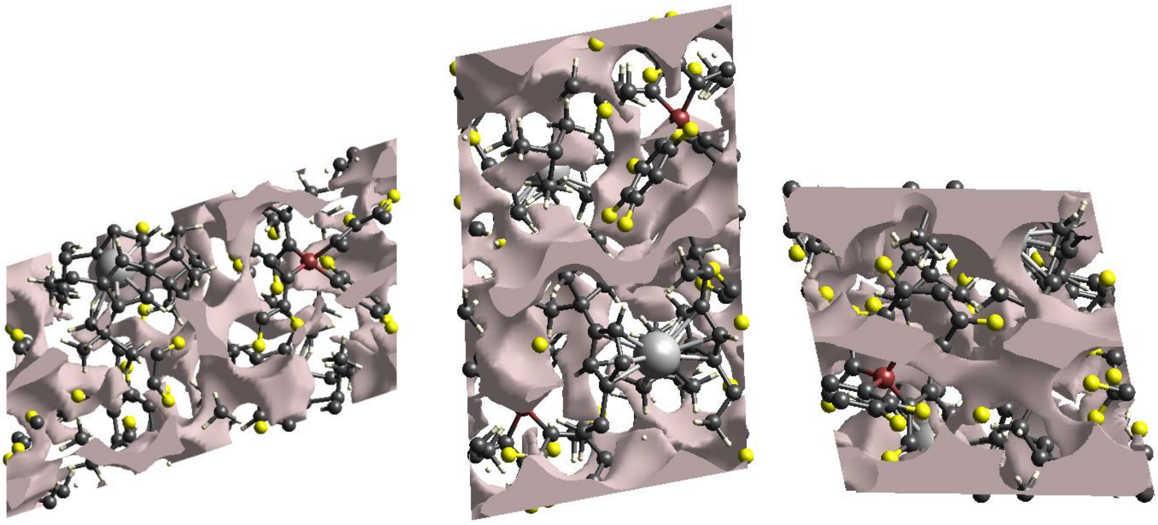

CHP3

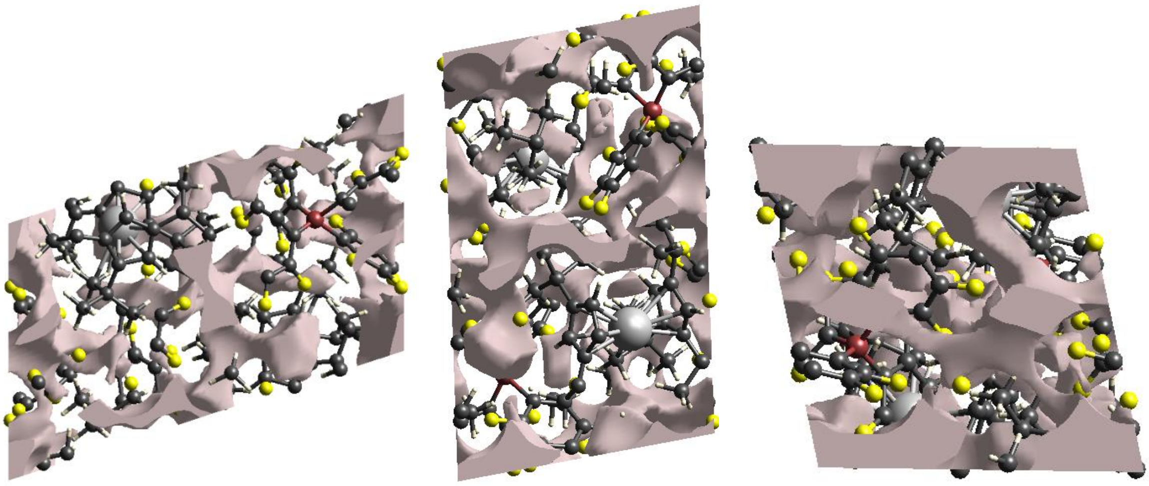

CHP4

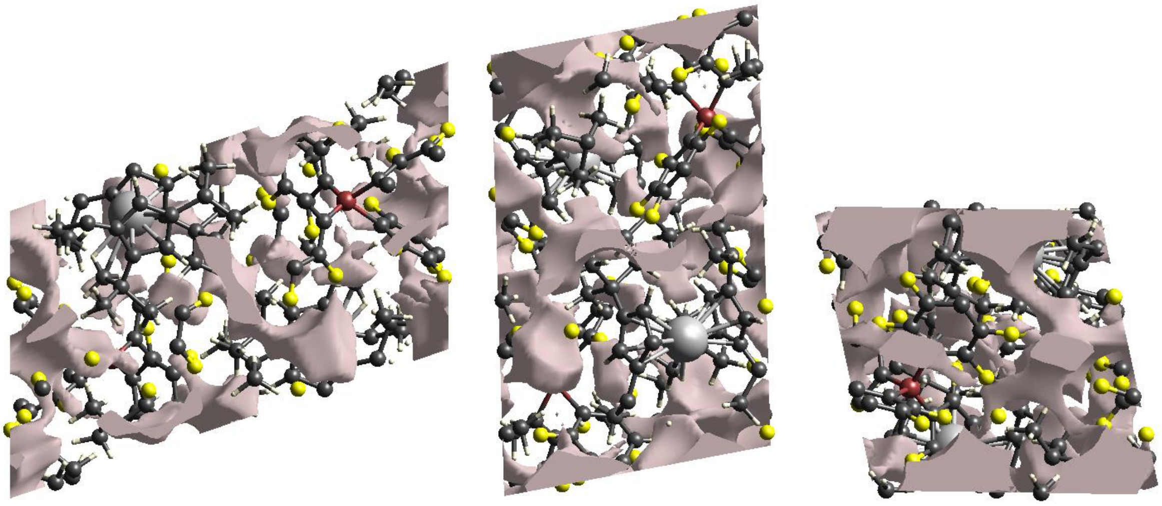

CHP5

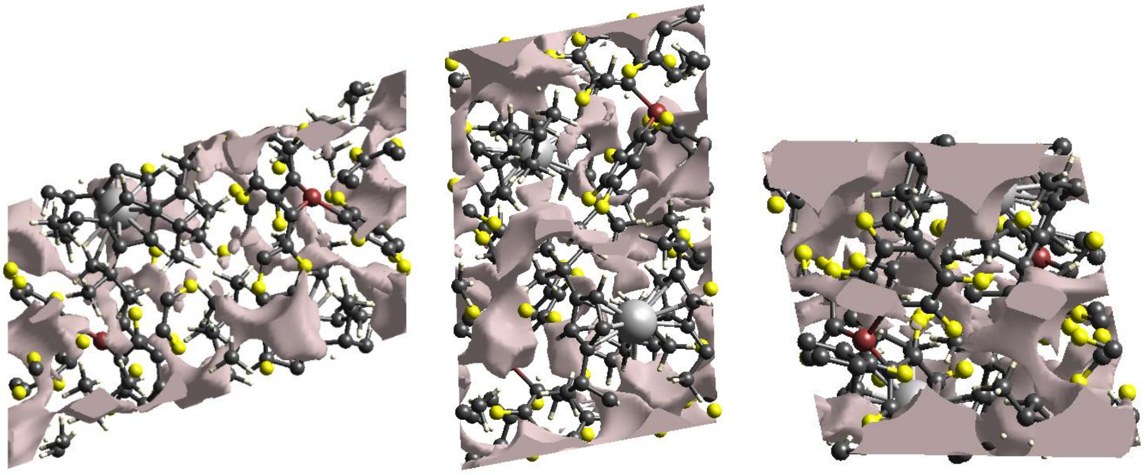

CHP6

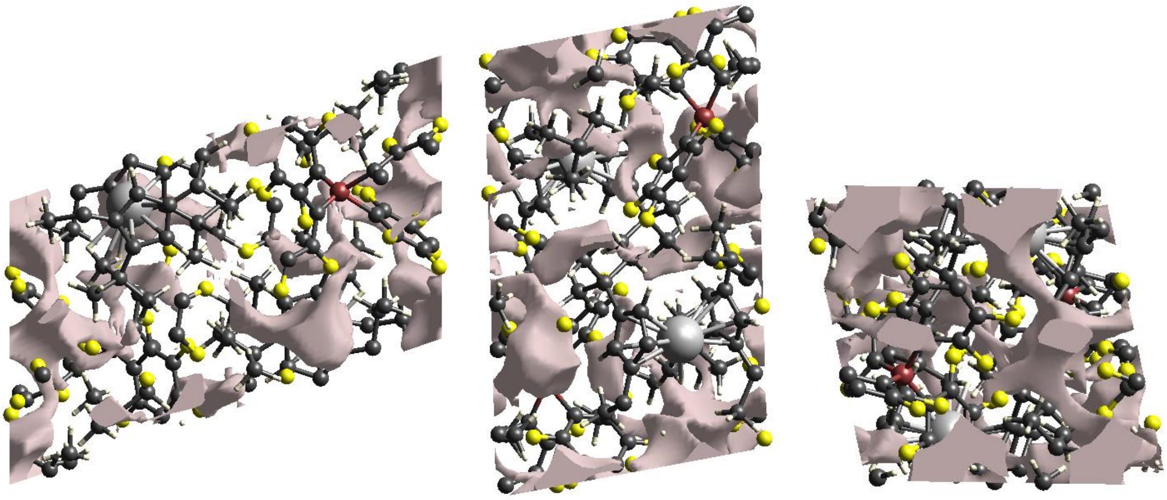

CHP7

Figure S11. Unit cell collected at different pressures with the void space drawn as a grey isosurface (0.002 e au^{-3}) calculated using CrystalExplorer.[7] The pressure increases from top to bottom, and three orientations are shown for each pressure.

Table S4. Calculated void volume and % void space in the unit cells collected at different pressures (isosurface value of 0.002 e au^{-3}).

Pressure (GPa)	Void Volume (Å^3)	% Void
0	469	16.9
0.41(6)	402	15.0
0.65(2)	318	12.2
1.01(6)	280	11.0
1.58*	233	9.4
2.12(8)	180	7.5
3.08(5)	141	6.0
3.52(5)	120	5.2

* Pressure estimated based on the unit-cell volume and fitted EoS.

The change in unit cell volume can be traced using the equation of state (Figure S4; Table S4). When pressurising the crystal from ambient conditions, initially the molecules start coming closer due to the squeezing-out of a lot of empty space in the crystal; at a pressure of 2.12 GPa, most of the void space in the crystal has been lost (Figure S11, Table S4) and the unit cell has significantly contracted hence pressurizing further reflects more on the changes in the molecule than the unit cell parameters.

Comparison with related Dysprosocenium crystal structures

In 2017, two different research groups reported an axial Dy complex with substituted cyclopentadienyl ligands $[Dy(Cp^{ttt})_2][B(C_6F_6)_4]\cdot0.5CH_2Cl_2$ (**1S**), where Cp^{ttt} will in the following be referred to as Cp for brevity. Metal ions sandwiched between two cyclopentadienyl ligands are referred to as metallocenium cations, and thus **1S** is sometimes referred to as a dysprosocenium cation.

The complexes from both studies crystallized in the triclinic space group *P*-1, with two molecular units as well as one solvent dichloromethane (DCM) in the unit cell. The two independently published structures and unit cells are slightly different. The **1S** structure reported by Goodwin *et al.*[1] will be referred to as "DyCp1" while the structure reported by Guo *et al.*[2] will be referred to as "DyCp2". In this work, we use a solvent-free analogue which has subsequently been synthesized by changing the crystallization solvent from DCM to fluorobenzene. This version will be referred to simply as **1**. Like the two previously-reported crystal structures, **1** adopts the triclinic space group *P*-1.

Table S5. Structural parameters for the three ambient-pressure DyCp structures.

	**1**	DyCp1	DyCp2
T (K)	100	150	150
Dy-$Cp_{1,cent}$ (Å)	2.321	2.314	2.309
Dy-$Cp_{2,cent}$ (Å)	2.322	2.318	2.324
$Cp_{1,cent}$-Dy-$Cp_{2,cent}$ (°)	153.44	152.56	152.845
Torsion angle (°)	58.35	73.96	73.75
Cp-Cp angle (°)	21.84	23.74	23.60
Dy-F10 (Å)	5.738	5.697	5.702
Dy-C7 (Å)	2.939	2.956	2.966
Dy-C24 (Å)	2.911	2.971	2.966
Dy-Dy (Å)	10.443	10.413	10.413
DyCp RMSD (Å)	-	0.4385	0.4999
DyCp Max D (Å)	-	1.2525	1.2863
DyCp1 RMSD (Å)	0.4385	-	0.2671
DyCp1 Max D (Å)	1.2525	-	1.1582

In **1**, the Cp ligands are almost equidistant from the Dy(III) ion with a mean distance between the Dy and Cp centroid of 2.32 Å. The angle formed between the Cp centroids and Dy is 153.44°. Both DyCp1 and DyCp2 feature similar ligand distances and angles. In order to determine the rotation of the Cp ligands relative to each other, we define a relevant torsion angle between C4, the centroid of Cp_1 (defined as the ring containing C1), the centroid of Cp_2 and C21 (see Figure 1 in the main text for labelling). With this definition, any multiple of 72° corresponds to the ligands being eclipsed. In **1**, the rings are in a staggered position with a torsion angle of 58°. This is very different from both DyCp1 and DyCp2, which exhibit nearly perfectly eclipsed ligands with a torsion angle of ~74°. The angle between the planes defined by the rings is 21.84° in **1** meaning they are slightly more parallel than in both of the previously-reported DCM

solvates (~24°). The shortest Dy-F distance is ~5.7 Å, which is too far for any coordination and thus in **1**, like in both previous structures, the anion is non-coordinating. In all three structures the shortest Dy-Dy distance is 10.4 Å at ambient pressure. As an additional means of quantifying the differences between the structures the Molecule Overlay function in Mercury was used. This function overlays two similar molecules and provides the root mean square deviation (RMSD) and the maximal deviation (Max D). This showed that out of the two original structures, **1** resembles DyCp1 more than DyCp2, but DyCp1 and DyCp2 are more alike due to the similar torsion angles.

3. Periodic DFT calculations

Periodic DFT calculations to obtain the phonon density of states (pDOS) were performed using VASP 5.4.4.[8–10] The Perdew−Burke−Ernzerhof (PBE) generalized-gradient approximation (GGA) functional with the DFT-D3 dispersion correction was used to model electron exchange and correlation.[11–13] We used plane-wave basis sets with a kinetic-energy cutoff of 850 eV and integrated the electronic Brillouin zone using the Γ-point. Both parameters were chosen to converge the absolute total energy of the ambient pressure crystal structure of **1** to within 1 meV atom^{-1} and the cell pressure to within 1 kbar (0.1 GPa). The use of the Γ-point to sample the BZ is also justified by the large unit-cell dimensions and the explicit convergence testing. We performed a geometry optimisation for each crystal structure collected at each pressure, allowing both atomic positions and the cell shape to optimise, but we fixed the cell volume to the experimentally-determined values to mimic the applied pressure. All optimisations were performed to tight energy convergence criteria of 10^{-8} eV on the total energy during the electronic minimisation and 10^{-3} eV Å^{-1} on the forces during structural relaxation.

Phonon frequencies were calculated using the supercell finite-displacement approach implemented in the Phonopy code,[14,15] with VASP as the force calculator. The second-order force constants were determined using 2×2×1 supercell expansions containing 1,104 atoms. During the single-point force calculations, additional support grids with 8× the number of points as the standard grids were used to ensure accurate forces. The pDOS curves were evaluated by interpolating the phonon frequencies onto uniform 8×8×8 Γ-centred grids of phonon wavevectors (*q*-points). Phonon dispersions were evaluated by interpolating the frequencies at strings of *q*-points passing through the high-symmetry points in the *P*-1 Brillouin zone. Despite the large supercells used to compute the force constants, there are still some small imaginary modes in the lowest-pressure dispersion plots (Figures S25 and S26), and the wavevectors of these modes suggest a 2×2×2 supercell expansion would be required to eliminate them. However, comparing the low-energy pDOS at these pressures to the higher pressures, at which we do not observe imaginary modes (Figures S27-31), shows a similar structure with an almost rigid shift in energy. We therefore do not believe that these parasitic imaginary modes result in significant error in the calculated pDOS cruves. We note, however, that all attempts to remove the imaginary modes in the 3.5 GPa structure failed, and we therefore do not provide data for this pressure.

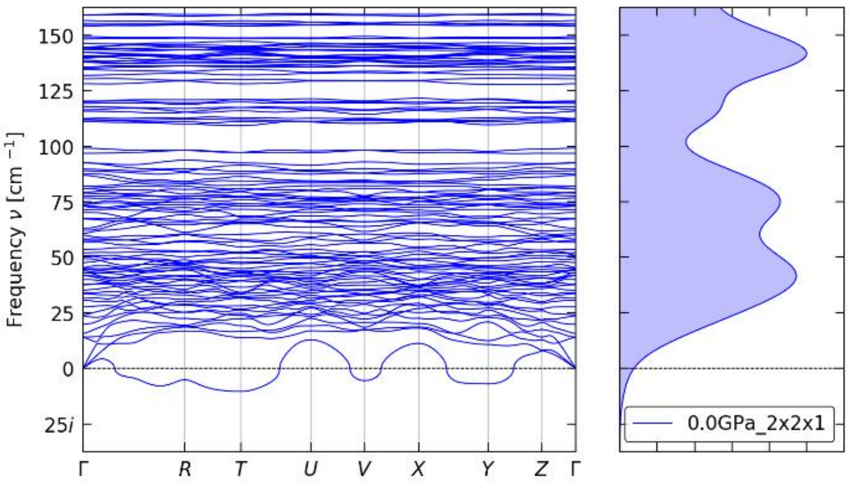

Figure S12. Calculated low-energy dispersion and pDOS of **1** at 0 GPa.

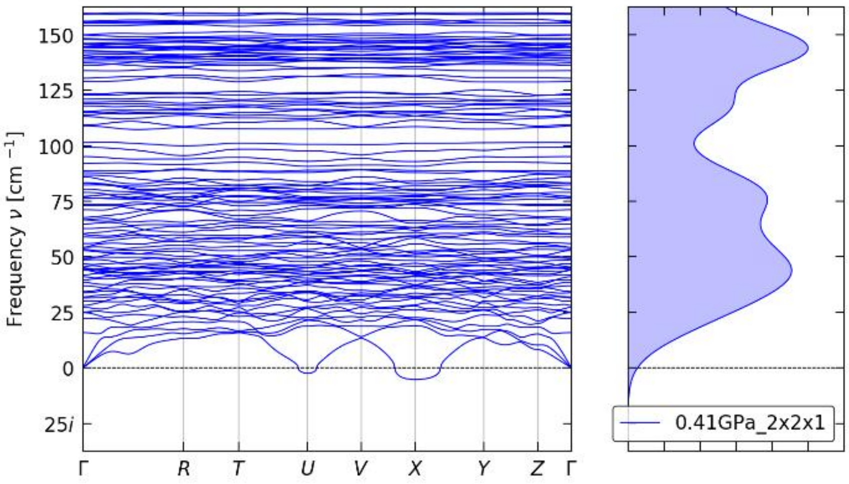

Figure S13. Calculated low-energy dispersion and pDOS of **1** at 0.41 GPa.

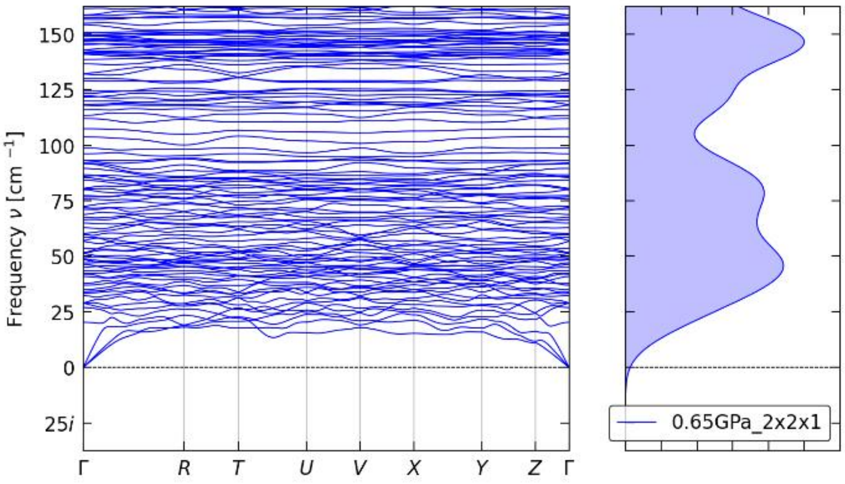

Figure S14. Calculated low-energy dispersion and pDOS of **1** at 0.65 GPa.

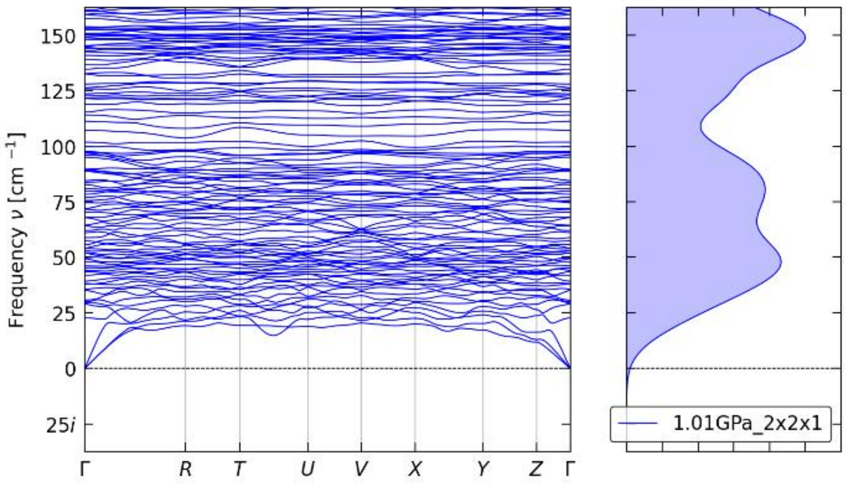

Figure S15. Calculated low-energy dispersion and pDOS of **1** at 1.01 GPa.

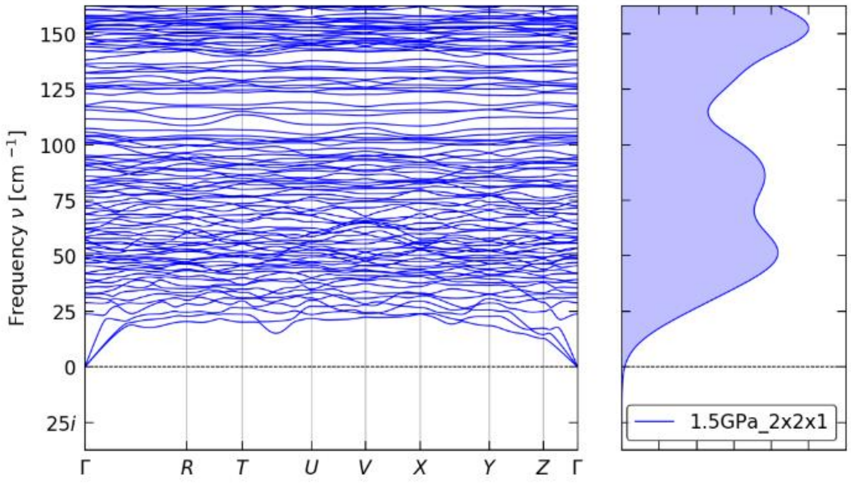

Figure S16. Calculated low-energy dispersion and pDOS of **1** at 1.5 GPa.

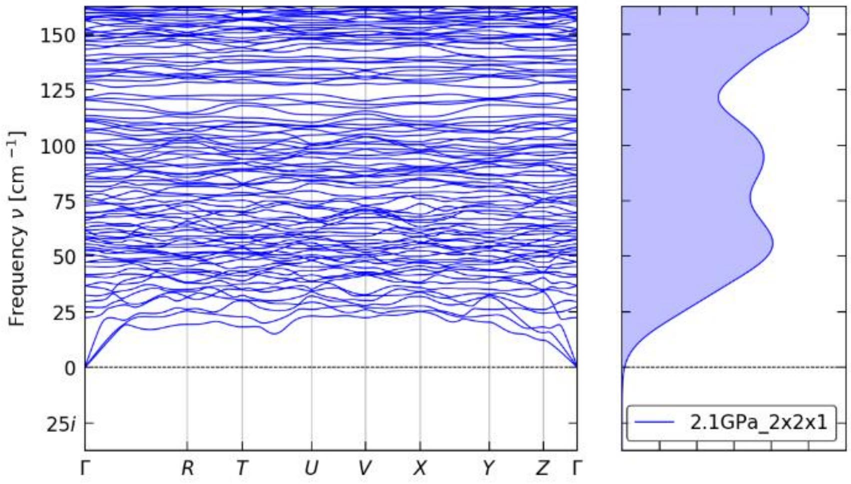

Figure S17. Calculated low-energy dispersion and pDOS of **1** at 2.1 GPa.

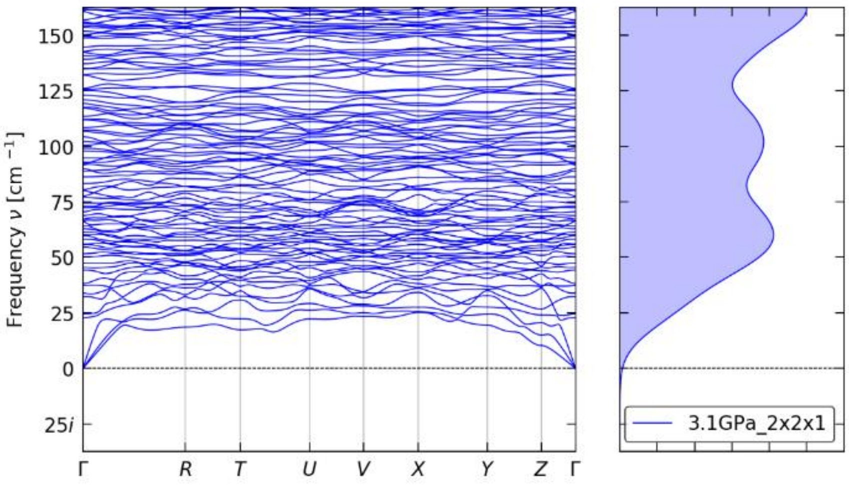

Figure S18. Calculated low-energy dispersion and pDOS of **1** at 3.1 GPa.

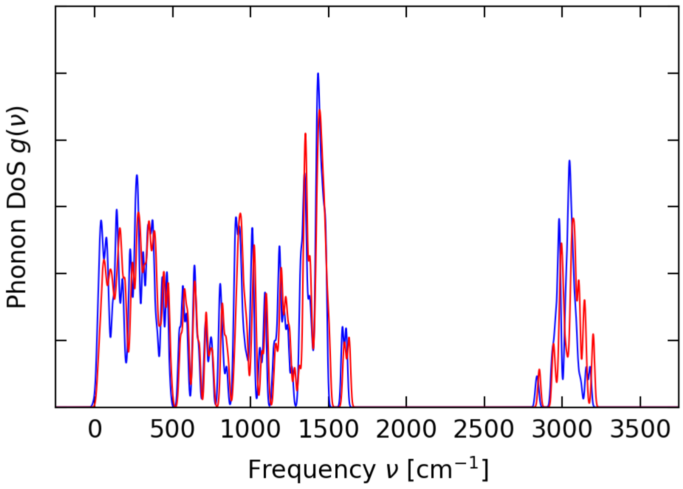

Figure S19. Comparison of the calculated pDOS of **1** at 0 GPa (blue) and 3.1 GPa (red).

4. CASSCF calculations

All CASSCF calculations were performed in ORCA 4.1.2.[16] The structures measured by X-ray diffraction were used in complete active space self-consistent field (CASSCF[17]) calculations performed with 9 electrons in 7 (4f) active orbitals, and 21 sextets were calculated.

For compound **1**, the SARC2-DKH-QZVP basis set and SARC2-DKH-QZVP/JK auxiliary basis set was used for Dy while DKH-Def2-TZVP and DKH-Def2-TZVP/JK was used for the remaining atoms.[18–20] These basis sets correspond to [8s7p5d3f2g1h] for Dy, [6s3p2d1f] for C and [3s1p] for H.

Using the results from the CASSCF calculations a second-order n-electron valence state perturbation theory (NEVPT2) calculation was performed to correct for dynamic correlation. After NEVPT2 the results were used in a quasi-degenerate perturbation theory calculation, which is performed to include spin-orbit coupling

We conducted a test on the ambient-pressure structure where the anion was also included in the calculation. This calculation was performed on the structures included in the asymmetric unit cell at ambient pressure, but due to memory limits, it had to be performed with much smaller basis sets. The complex itself was therefore also recalculated using the smaller basis sets for comparison, which also makes comparison between results obtained with different basis set sizes possible. The basis sets used for Dy were SARC2-DKH-QZV (SARC2-DKH-QZVP/JK) while DKH-Def2-SVP (DKH-Def2-SVP/JK) were used for the remaining atoms. Changing to smaller basis sets had almost no effect on the energy levels (<20 cm^{-1}) and led to no significant changes in g-values and angles.

Inclusion of an anion also did not seem to have a large effect, with only small changes in the energy levels and almost no changes in the g-values. It is possible that a larger effect would be seen at higher pressures or if the anion containing the shortest Dy-F distance was included instead. However, the anion is still far from the complex, even at the highest pressure.

We note that the results for the 3.52(5) GPa structure suggest an increase in U_{eff} to ~1300 cm^{-1}, so we posit that the highest-pressure XRD structure may be less reliable than the others.

Upon pressurising and the resultant bending of the molecules, the C7 and C24 carbons from the tBu groups come significantly closer to the Dy(III) centre. Consequently, the Hydrogens on the C7 and C24 carbons may project electron density in the equatorial plane close to Dy(III) introducing the transverse $B_k^{q\neq0}$ crystal field components resulting in reduced magnetic anisotropy. These effects are included in the CASSCF calculations shown in Figure S20 and Tables S6-S15.

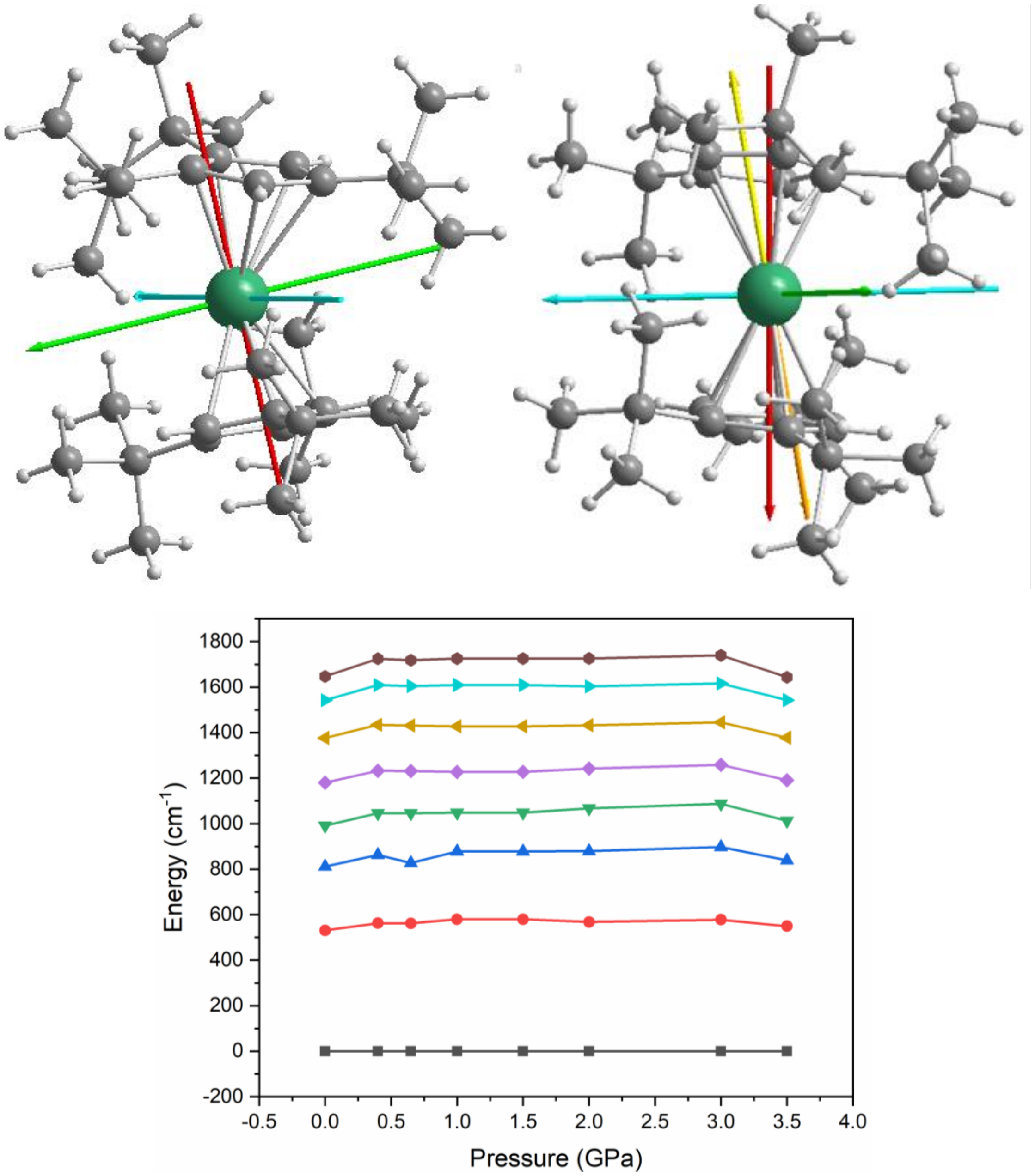

Figure S20. Eigenvectors for g_z of the g-tensor at ambient pressure (top-left) and 1.5 GPa (top-right), shown for the ground state Kramers doublet (red) and the 5th (orange), 6th (yellow), 7th (green) and 8th (cyan) KDs of **1**. The energies of the KDs as a function of pressure are shown in the plot on the bottom: 1st - black, 2nd - red, 3rd - blue, 4th - green, 5th - purple, 6th - ochre, 7th - cyan and 8th - brown.

Table S6. Energies, g-values and wavefunction composition of the lowest KDs for HP0.

E(CASSCF) / cm^{-1}	E(NEVPT2) / cm^{-1}	g_x	g_y	g_z	∠g_z / °	Wavefunction
0	0	0	0	19.98	-	99.8%|±15/2>+0.2%|±13/2>
491.14	531.04	0	0	17.01	0.85	99.9%|±13/2>
777.03	811.94	0	0	14.42	1.62	99.3%|±11/2>+0.4%|±7/2>+0.2%|±15/2>+0.1%|±9/2>
960.58	991.68	0.03	0.04	11.76	2.54	99.0%|±9/2>+0.8%|±5/2>+0.1%|±11/2>
1126.45	1179.56	0.18	0.25	9.03	2.82	99.1%|±7/2>+0.4%|±3/2>+0.4%|±11/2>+0.1%|±1/2>
1286.08	1376.56	1.34	1.67	6.31	2.25	98.5%|±5/2>+0.8%|±9/2>+0.6%|±3/2>+0.1%|±1/2>
1414.87	1542.30	2.1	3.55	5.69	89.61	95.4%|±3/2>+3.5%|±1/2>+0.6%|±5/2>+0.4%|±7/2>
1495.90	1646.62	1.07	6.43	14.06	89.83	96.3%|±1/2>+3.6%|±3/2>+0.1%|±5/2>+0.1%|±7/2>

Table S7. Energies, g-values and wavefunction composition of the lowest KDs for HP0 obtained with the smaller basis set.

E(CASSCF) / cm^{-1}	E(NEVPT2) / cm^{-1}	g_x	g_y	g_z	∠g_z / °	Wavefunction
0	0	0	0	19.98	-	99.8%|±15/2>+0.2%|±11/2>
491.19	543.19	0	0	17	0.80	99.9%|±13/2>+0.1%|±9/2>
771.59	831.21	0.01	0.01	14.42	1.43	99.3%|±11/2>+0.3%|±7/2>+0.2%|±15/2>+0.1%|±9/2>
948.74	1009.76	0.04	0.05	11.76	2.64	99.0%|±9/2>+0.8%|±5/2>+0.1%|±11/2>+0.1%|±13/2>
1111.81	1192.94	0.22	0.31	9.04	2.71	99.2%|±7/2>+0.4%|±11/2>+0.4%|±3/2>+0.1%|±1/2>+0.1%|±5/2>
1271.29	1384.65	1.35	1.76	6.32	2.07	98.4%|±5/2>+0.7%|±9/2>+0.6%|±3/2>+0.1%|±1/2>+0.1%|±7/2>
1400.60	1545.76	2.43	3.51	6.22	89.77	94.5%|±3/2>+4.4%|±1/2>+0.6%|±5/2>+0.4%|±7/2>
1485.05	1650.37	1.03	5.96	14.44	89.44	94.5%|±1/2>+4.4%|±3/2>+0.2%|±5/2>+0.1%|±7/2>

Table S8. Energies, g-values and wavefunction composition of the lowest KDs for CHP0 with the smaller basis set and including the anion.

<table>
<tr><th>E(CASSCF) / cm^{-1}</th><th>E(NEVPT2) / cm^{-1}</th><th>g_x</th><th>g_y</th><th>g_z</th><th>$\angle g_z$ / °</th><th>Wavefunction</th></tr>
<tr><td>0</td><td>0</td><td>0</td><td>0</td><td>19.87</td><td>-</td><td>99.8%|±15/2>+0.2%|±11/2></td></tr>
<tr><td>498.80</td><td>552.93</td><td>0</td><td>0</td><td>16.94</td><td>0.68</td><td>99.9%|±13/2></td></tr>
<tr><td>781.99</td><td>843.96</td><td>0</td><td>0</td><td>14.38</td><td>1.13</td><td>99.3%|±11/2>+0.4%|±7/2>+0.2%|±15/2>+0.1%|±9/2></td></tr>
<tr><td>959.89</td><td>1022.64</td><td>0.03</td><td>0.04</td><td>11.71</td><td>2.53</td><td>99.0%|±9/2>+0.8%|±5/2>+0.1%|±11/2></td></tr>
<tr><td>1123.86</td><td>1206.61</td><td>0.18</td><td>0.25</td><td>8.99</td><td>2.51</td><td>99.1%|±7/2>+0.4%|±3/2>+0.4%|±11/2>+0.1%|±1/2></td></tr>
<tr><td>1285.10</td><td>1400.54</td><td>1.34</td><td>1.67</td><td>6.26</td><td>1.94</td><td>98.5%|±5/2>+0.8%|±9/2>+0.6%|±3/2>+0.1%|±1/2></td></tr>
<tr><td>1416.88</td><td>1564.73</td><td>2.1</td><td>3.55</td><td>6.3</td><td>89.75</td><td>95.4%|±3/2>+3.5%|±1/2>+0.6%|±5/2>+0.4%|±7/2></td></tr>
<tr><td>1504.00</td><td>1672.54</td><td>1.07</td><td>6.43</td><td>14.48</td><td>89.40</td><td>96.3%|±1/2>+3.6%|±3/2>+0.1%|±5/2>+0.1%|±7/2></td></tr>
</table>

Table S9. Energies, g-values and wavefunction composition of the lowest KDs for CHP1.

<table>
<tr><th>E(CASSCF) / cm^{-1}</th><th>E(NEVPT2) / cm^{-1}</th><th>g_x</th><th>g_y</th><th>g_z</th><th>$\angle g_z$ / °</th><th>Wavefunction</th></tr>
<tr><td>0</td><td>0</td><td>0</td><td>0</td><td>19.98</td><td>-</td><td>99.8%|±15/2>+0.2%|±11/2></td></tr>
<tr><td>517.93</td><td>562.55</td><td>0</td><td>0</td><td>16.99</td><td>1.48</td><td>99.8%|±13/2>+0.1%|±11/2>+0.1%|±9/2></td></tr>
<tr><td>810.58</td><td>862.91</td><td>0.01</td><td>0.01</td><td>14.42</td><td>2.94</td><td>99.1%|±11/2>+0.4%|±9/2>+0.2%|±15/2>+0.2%|±7/2>+0.1%|±13/2></td></tr>
<tr><td>996.31</td><td>1045.88</td><td>0.02</td><td>0.03</td><td>11.81</td><td>4.85</td><td>98.9%|±9/2>+0.5%|±5/2>+0.4%|±11/2>+0.1%|±13/2>+0.1%|±3/2></td></tr>
<tr><td>1162.24</td><td>1232.05</td><td>0.26</td><td>0.31</td><td>9.07</td><td>5.75</td><td>99.3%|±7/2>+0.2%|±11/2>+0.2%|±3/2>+0.2%|±5/2>+0.1%|±1/2></td></tr>
<tr><td>1328.61</td><td>1433.96</td><td>0.65</td><td>1.17</td><td>6.33</td><td>4.39</td><td>98.5%|±5/2>+0.6%|±3/2>+0.5%|±9/2>+0.2%|±7/2>+0.2%|±1/2></td></tr>
<tr><td>1467.15</td><td>1608.88</td><td>2.81</td><td>3.59</td><td>5.18</td><td>89.78</td><td>94.8%|±3/2>+4.3%|±1/2>+0.5%|±5/2>+0.2%|±7/2></td></tr>
<tr><td>1557.44</td><td>1724.61</td><td>1.04</td><td>6.27</td><td>14.21</td><td>88.98</td><td>95.39%|±1/2>+4.3%|±3/2>+0.2%|±5/2>+0.1%|±7/2></td></tr>
</table>

Table S10. Energies, g-values and wavefunction composition of the lowest KDs for CHP2.

E(CASSCF) / cm^{-1}	E(NEVPT2) / cm^{-1}	g_x	g_y	g_z	$\angle g_z$ / °	Wavefunction
0	0	0	0	19.98	-	99.8%|±15/2>+0.2%|±11/2>
516.62	561.52	0	0	16.99	1.66	99.8%|±13/2>+0.1%|±11/2>+0.1%|±9/2>
808.01	826.69	0.01	0.01	14.42	3.50	98.8%|±11/2>+0.6%|±9/2>+0.3%|±7/2>+0.2%|±15/2>+0.1%|±11/2>
992.47	1045.85	0.04	0.06	11.81	5.10	98.6%|±9/2>+0.6%|±5/2>+0.6%|±11/2>+0.1%|±13/2>+0.1%|±3/2>
1156.67	1230.87	0.47	0.58	9.07	6.27	99.1%|±7/2>+0.3%|±11/2>+0.3%|±3/2>+0.2%|±5/2>+0.1%|±1/2>
1321.78	1431.26	0.71	1.67	6.31	4.87	98.2%|±5/2>+0.7%|±3/2>+0.6%|±9/2>+0.2%|±1/2>+0.2%|±7/2>
1458.97	1604.64	2.51	3.56	5.46	87.90	94.7%|±3/2>+4.2%|±1/2>+0.7%|±5/2>+0.4%|±7/2>+0.1%|±9/2>
1547.89	1718.18	1.05	6.31	14.2	89.13	95.41%|±1/2>+4.4%|±3/2>+0.3%|±5/2>+0.1%|±7/2>

Table S11. Energies, g-values and wavefunction composition of the lowest KDs for CHP3.

E(CASSCF) / cm^{-1}	E(NEVPT2) / cm^{-1}	g_x	g_y	g_z	$\angle g_z$ / °	Wavefunction
0	0	0	0	19.98	-	99.9%|±15/2>+0.1%|±11/2>
533.19	579.80	0	0	16.97	0.59	99.9%|±13/2>+0.1%|±9/2>
820.95	878.50	0	0.01	14.41	1.21	99.5%|±11/2>+0.3%|±7/2>+0.1%|±15/2>+0.1%|±9/2>
994.80	1048.72	0	0.1	11.79	3.15	99.2%|±9/2>+0.6%|±5/2>+0.1%|±11/2>+0.1%|±13/2>+0.1%|±3/2>
1155.96	1227.49	0.21	0.23	9.04	3.25	99.1%|±7/2>+0.4%|±3/2>+0.3%|±11/2>+0.1%|±5/2>+0.1%|±1/2>
1322.67	1427.42	1.64	1.97	6.26	2.83	97.9%|±5/2>+1.0%|±3/2>+0.6%|±9/2>+0.3%|±1/2>+0.2%|±7/2>
1467.38	1609.31	1.48	3.53	5.3	88.52	95.6%|±3/2>+2.9%|±1/2>+1.1%|±5/2>+0.4%|±7/2>+0.1%|±9/2>
1558.17	1725.13	1.09	6.98	13.7	89.43	96.7%|±1/2>+3.0%|±3/2>+0.2%|±5/2>+0.1%|±7/2>

Table S12. Energies, g-values and wavefunction composition of the lowest KDs for CHP4.

<table>
<tr><th>E(CASSCF) / cm^{-1}</th><th>E(NEVPT2) / cm^{-1}</th><th>g_x</th><th>g_y</th><th>g_z</th><th>$\angle g_z$ / °</th><th>Wavefunction</th></tr>
<tr><td>0</td><td>0</td><td>0</td><td>0</td><td>19.98</td><td>-</td><td>99.8%|±15/2>+0.2%|±11/2></td></tr>
<tr><td>526.37</td><td>571.73</td><td>0</td><td>0</td><td>16.98</td><td>1.59</td><td>99.8%|±13/2>+0.1%|±11/2>+0.1%|±9/2></td></tr>
<tr><td>822.79</td><td>880.09</td><td>0</td><td>0.01</td><td>14.41</td><td>3.34</td><td>99.0%|±11/2>+0.2%|±7/2>+0.5%|±9/2>+0.2%|±15/2>+0.1%|±13/2></td></tr>
<tr><td>1009.92</td><td>1067.40</td><td>0.12</td><td>0.13</td><td>11.8</td><td>4.48</td><td>98.8%|±9/2>+0.5%|±11/2>+0.4%|±7/2>+0.1%|±3/2>+0.1%|±13/2></td></tr>
<tr><td>1175.59</td><td>1254.23</td><td>1.41</td><td>1.68</td><td>8.97</td><td>6.39</td><td>98.8%|±7/2>+0.7%|±5/2>+0.2%|±3/2>+0.2%|±11/2>+0.1%|±1/2></td></tr>
<tr><td>1347.15</td><td>1459.53</td><td>0.08</td><td>3.1</td><td>6.19</td><td>6.33</td><td>97.7%|±5/2>+1.0%|±3/2>+0.7%|±7/2>+0.4%|±9/2>+0.3%|±1/2></td></tr>
<tr><td>1487.68</td><td>1637.48</td><td>2.35</td><td>3.49</td><td>6.01</td><td>88.53</td><td>94.1%|±3/2>+4.5%|±1/2>+0.9%|±5/2>+0.3%|±7/2>+0.1%|±9/2></td></tr>
<tr><td>1580.20</td><td>1755.47</td><td>1.02</td><td>6.1</td><td>14.39</td><td>89.07</td><td>95.0%|±1/2>+4.6%|±3/2>+0.3%|±5/2>+0.1%|±7/2></td></tr>
</table>

Table S13. Energies, g-values and wavefunction composition of the lowest KDs for CHP5.

<table>
<tr><th>E(CASSCF) / cm^{-1}</th><th>E(NEVPT2) / cm^{-1}</th><th>g_x</th><th>g_y</th><th>g_z</th><th>$\angle g_z$ / °</th><th>Wavefunction</th></tr>
<tr><td>0</td><td>0</td><td>0</td><td>0</td><td>19.98</td><td>-</td><td>99.8%|±15/2>+0.2%|±11/2></td></tr>
<tr><td>524.33</td><td>567.99</td><td>0</td><td>0</td><td>16.99</td><td>2.92</td><td>99.4%|±13/2>+0.4%|±11/2>+0.1%|±9/2></td></tr>
<tr><td>817.93</td><td>879.72</td><td>0.01</td><td>0.01</td><td>14.44</td><td>6.15</td><td>97.4%|±11/2>+1.9%|±9/2>+0.4%|±13/2>+0.2%|±15/2>+0.1%|±7/2></td></tr>
<tr><td>1004.70</td><td>1067.21</td><td>0.11</td><td>0.12</td><td>11.89</td><td>6.79</td><td>97.2%|±9/2>+1.9%|±11/2>+0.3%|±5/2>+0.2%|±7/2>+0.2%|±13/2>+0.1%|±3/2 ></td></tr>
<tr><td>1162.66</td><td>1241.80</td><td>1.04</td><td>1.31</td><td>9.14</td><td>11.00</td><td>98.7%|±7/2>+0.5%|±5/2>+0.3%|±9/2>+0.3%|±1/2>+0.2%|±3/2>+0.1%|±11/2></td></tr>
<tr><td>1323.69</td><td>1432.20</td><td>1.46</td><td>3.71</td><td>6.18</td><td>12.03</td><td>96.2%|±5/2>+2.3%|±3/2>+0.7%|±1/2>+0.5%|±7/2>+0.3%|±9/2></td></tr>
<tr><td>1459.85</td><td>1603.06</td><td>2.56</td><td>3.34</td><td>8.44</td><td>89.19</td><td>89.1%|±3/2>+8.3%|±1/2>+2.1%|±5/2>+0.3%|±7/2>+0.2%|±9/2></td></tr>
<tr><td>1556.48</td><td>1726.00</td><td>0.87</td><td>4.6</td><td>15.58</td><td>88.66</td><td>90.7%|±1/2>+8.4%|±3/2>+0.7%|±5/2>+0.2%|±7/2></td></tr>
</table>

Table S14. Energies, g-values and wavefunction composition of the lowest KDs for CHP6.

<table>
<tr><th>E(CASSCF) / cm^{-1}</th><th>E(NEVPT2) / cm^{-1}</th><th>g_x</th><th>g_y</th><th>g_z</th><th>∠g_z / °</th><th>Wavefunction</th></tr>
<tr><td>0</td><td>0</td><td>0</td><td>0</td><td>19.97</td><td>-</td><td>99.8%|±15/2>+0.2%|±11/2></td></tr>
<tr><td>532.60</td><td>578.01</td><td>0</td><td>0</td><td>16.98</td><td>3.50</td><td>99.2%|±13/2>+0.6%|±11/2>+0.2%|±9/2></td></tr>
<tr><td>830.35</td><td>897.60</td><td>0.02</td><td>0.03</td><td>14.43</td><td>6.83</td><td>96.5%|±11/2>+2.4%|±9/2>+0.5%|±13/2>+0.3%|±7/2>+0.2%|±11/2></td></tr>
<tr><td>1016.66</td><td>1087.01</td><td>0.2</td><td>0.25</td><td>11.93</td><td>8.70</td><td>96.2%|±9/2>+2.5%|±13/2>+0.6%|±5/2>+0.2%|±7/2>+0.2%|±3/2>+0.1%|±13/2 ></td></tr>
<tr><td>1170.86</td><td>1258.06</td><td>1.12</td><td>1.61</td><td>9.15</td><td>12.96</td><td>97.7%|±7/2>+0.9%|±5/2>+0.5%|±3/2>+0.3%|±1/2>+0.3%|±9/2>+0.2%|±11/2></td></tr>
<tr><td>1329.59</td><td>1444.68</td><td>2.02</td><td>4.75</td><td>6.18</td><td>25.22</td><td>93.3%|±5/2>+3.7%|±3/2>+1.6%|±1/2>+0.7%|±7/2>+0.6%|±9/2></td></tr>
<tr><td>1466.07</td><td>1616.21</td><td>2.28</td><td>3.16</td><td>9.5</td><td>90.98</td><td>85.3%|±3/2>+9.5%|±1/2>+4.2%|±5/2>+0.8%|±7/2>+0.2%|±9/2></td></tr>
<tr><td>1564.08</td><td>1739.61</td><td>0.82</td><td>4.1</td><td>15.96</td><td>91.30</td><td>88.6%|±1/2>+10.2%|±3/2>+1.0%|±5/2>+0.2%|±7/2></td></tr>
</table>

Table S15. Energies, g-values and wavefunction composition of the lowest KDs for CHP7.

<table>
<tr><th>E(CASSCF) / cm^{-1}</th><th>E(NEVPT2) / cm^{-1}</th><th>g_x</th><th>g_y</th><th>g_z</th><th>∠g_z / °</th><th>Wavefunction</th></tr>
<tr><td>0</td><td>0</td><td>0</td><td>0</td><td>19.98</td><td>-</td><td>99.8%|±15/2>+0.2%|±11/2></td></tr>
<tr><td>505.21</td><td>549.21</td><td>0</td><td>0</td><td>16.99</td><td>1.24</td><td>99.8%|±13/2>+0.1%|±9/2>+0.1%|±11/2></td></tr>
<tr><td>791.88</td><td>839.16</td><td>0.01</td><td>0.01</td><td>14.4</td><td>2.03</td><td>98.8%|±11/2>+0.6%|±7/2>+0.2%|±9/2>+0.2%|±15/2>+0.1%|±13/2></td></tr>
<tr><td>968.17</td><td>1013.09</td><td>0.07</td><td>0.09</td><td>11.76</td><td>4.47</td><td>98.3%|±9/2>+1.3%|±5/2>+0.2%|±11/2>+0.1%|±13/2>+0.1%|±3/2></td></tr>
<tr><td>1125.90</td><td>1191.37</td><td>0.12</td><td>0.28</td><td>9.03</td><td>4.34</td><td>98.0%|±7/2>+0.9%|±3/2>+0.6%|±11/2>+0.3%|±1/2>+0.1%|±5/2></td></tr>
<tr><td>1278.83</td><td>1377.81</td><td>2.99</td><td>3.52</td><td>6.14</td><td>5.89</td><td>94.99%|±5/2>+2.7%|±3/2>+1.3%|±9/2>+0.9%|±1/2>+0.2%|±7/2></td></tr>
<tr><td>1407.63</td><td>1542.76</td><td>1.15</td><td>3.3</td><td>7.94</td><td>88.19</td><td>91.1%|±3/2>+4.7%|±1/2>+3.0%|±5/2>+1.0%|±7/2>+0.1%|±9/2></td></tr>
<tr><td>1487.14</td><td>1643.32</td><td>1.01</td><td>5.69</td><td>14.7</td><td>89.56</td><td>94.1%|±1/2>+5.4%|±3/2>+0.4%|±5/2>+0.1%|±7/2></td></tr>
</table>

5. Ambient pressure magnetic measurements

Magnetic measurements at ambient pressure were performed on a Quantum Design MPMS3 superconducting quantum interference device (SQUID) magnetometer. All dc measurements were taken in dc scan measurement mode with a 30 mm scan length and 4 s scan time. A sample of **1** (29.3 mg) restrained in eicosane (16.9 mg) was prepared as described previously.[1] Raw magnetic data were corrected for the diamagnetic contribution of the sample holder (straw + borosilicate tube) and eicosane, corrected for the shape of the sample by dividing by 0.963 (calculated with Quantum Design MPMS3 Geometry Simulator assuming a uniform cylinder of diameter 4.06 mm and height of 5.85 mm) and corrected for the intrinsic diamagnetic contribution of the sample, estimated as the molecular weight (g mol^{-1}) multiplied by 0.5 x 10^{-6} cm^3 K mol^{-1}.

The equilibrium susceptibility was measured in temperature settle mode on cooling under a 1 kOe static applied field. From 300 to 100 K, measurements were recorded every 20 K with a cooling rate of 10 K min^{-1} and a 2.5 min delay on measuring each point. From 90 to 60 K, measurements were recorded every 10 K with a cooling rate of 5 10 K min^{-1} and a 10 min delay on measuring each point. From 50 to 1.8 K, 21 data points were measured evenly spaced in log *T*. Between 50 and 10 K, 10 and 5 K and 5 and 1.8 K, the cooling rates were 2, 1 and 1 K min^{-1}, respectively, and the delays before measuring were 20, 30 and 30 min respectively.

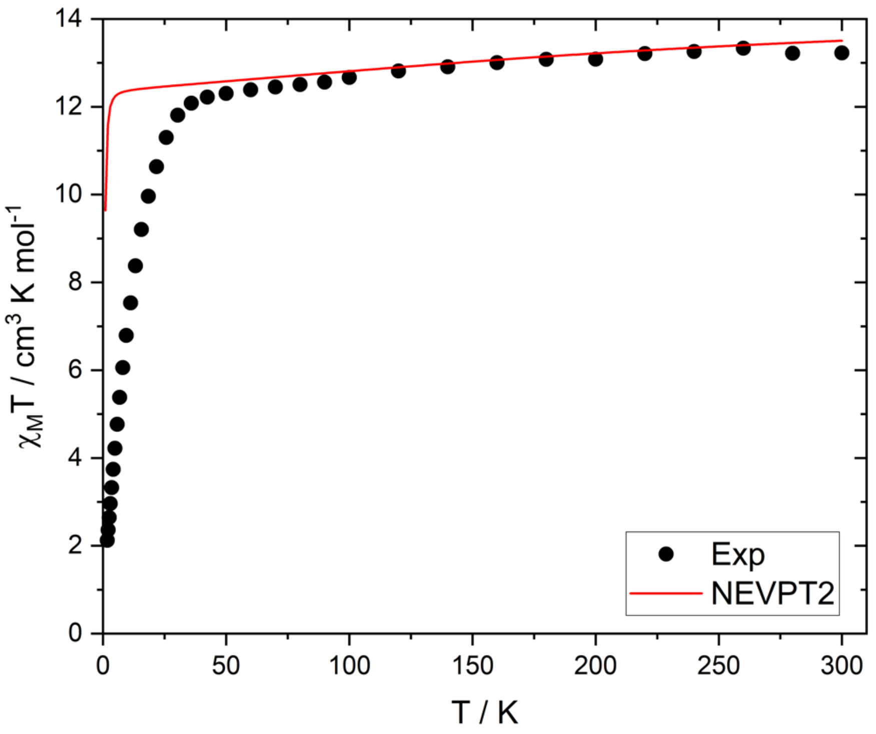

Figure S21. Equilibrium magnetic susceptibility $\chi_M T$ vs T for **1** sealed in an NMR tube under vacuum and a 1000 G applied field (black dots) and susceptibility calculated for the entire asymmetric unit using ORCA (red line).

Direct current hysteresis measurements were made by magnetising the samples at 70 kOe and sweeping the field through 0 to -70 kOe then back through 0 to 70 kOe at constant temperature. The measurement sequence was repeated at 2 K, 5-50 K in 5 K intervals and 52-70 K in 2 K intervals. Measurements were made in continuous field sweep mode, with slight delays between sections of different rates. Any spurious points were removed. For 20 < |H| ≤ 70 kOe, measurements were recorded in approximately 1250 Oe steps with an average sweep rate of 90.6(2) Oe s^{-1}. For 10 < |H| ≤ 20 kOe, measurements were recorded approximately every 1000 Oe with an average sweep rate of 51.9(8) Oe s^{-1}. For -10 ≤ H ≤ 10 kOe, measurements were recorded approximately every 250 Oe with an average sweep rate of 21.96(1) Oe s^{-1}. The sweep rates were chosen to closely match the sweep rates in the measurements performed on **1** under high pressure and in those previously reported for **1S**.[1] The sweep rate around zero field is sufficiently slow to give reliable hysteresis loops, and these measurements are in excellent agreement with those previously reported for **1S**.[1]

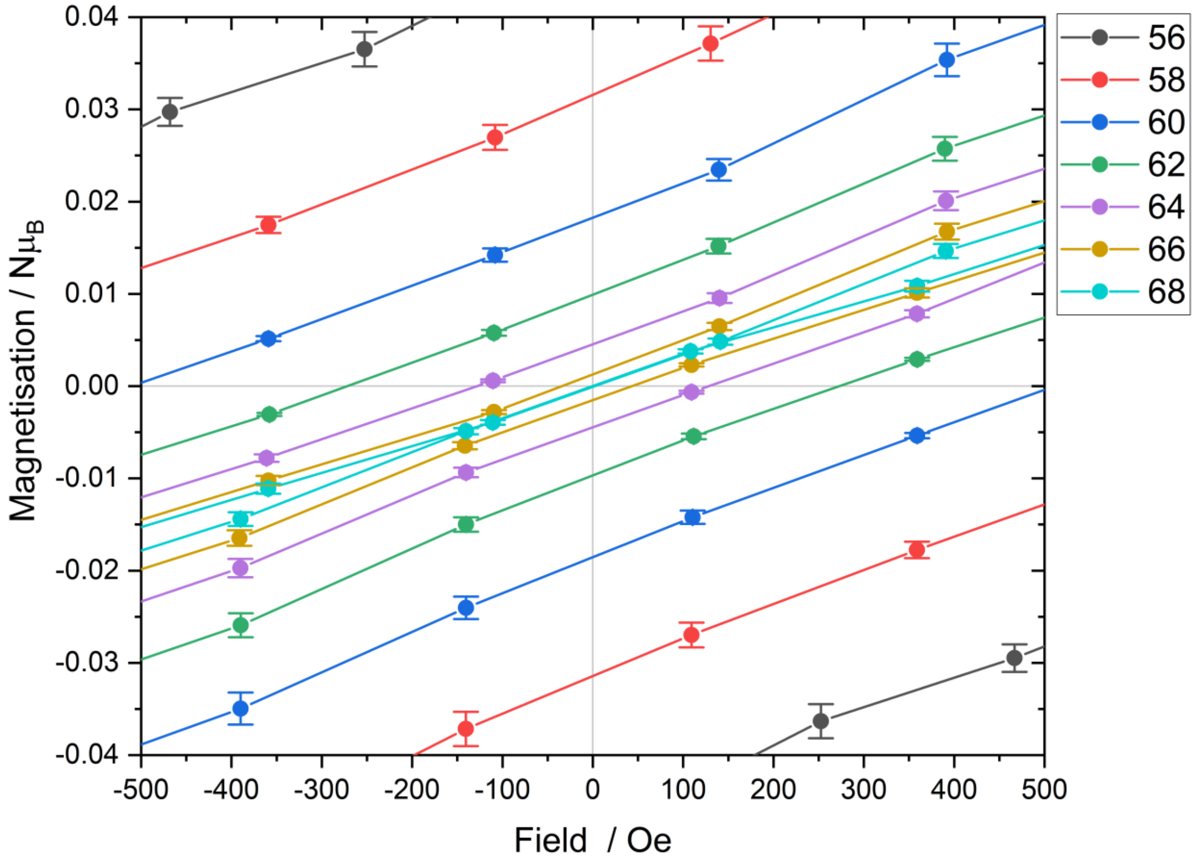

Figure S22. Hysteresis loops at 56-68 K in 2 K steps showing a closing of the hysteresis loop above 66 K. The sweep rate is 90.6(2) Oe s^{-1} for 20 < |H| ≤ 70 kOe, 51.9(8) Oe s^{-1} for 10 < |H| ≤ 20 kOe and 21.96(1) Oe s^{-1} for -10 ≤ H ≤ 10 kOe.

The sample of **1** in eicosane was prepared for the zero-field cooled susceptibility by holding the sample at 150 K for 10 min in zero field, rapidly cooling to 10 K at 30 K min^{-1} and holding for 5 min, and then cooling to 2 K at 5 K min^{-1} and holding for 30 min. The field was then ramped to 500 or 1 kOe at 100 Oe s^{-1} and held constant, at which point the temperature was swept continually to 100 K at a warming rate of (+)0.9 K min^{-1} and the moment was measured approximately every 2 K. After waiting for 20 min at 100 K, the field-cooled trace was obtained by measuring the susceptibility approximately every 2 K while continually cooling at (-)0.9 K min^{-1}. The ZFC and FC traces were considered to have converged when they agreed within 2% of the normalised susceptibility value; this threshold was determined by analogous measurements on a paramagnet without slow relaxation behaviour mounted under the same conditions.

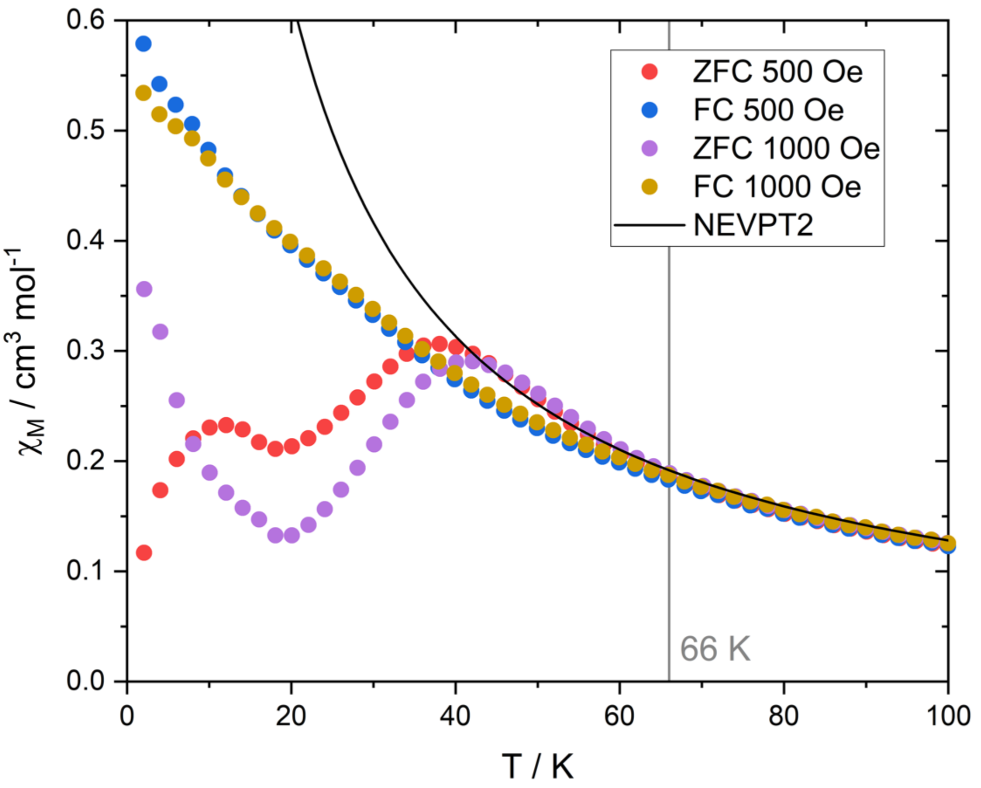

Figure S23. Plot of χ_M vs T for **1** measured at 500 or 1000 Oe on warming after cooling in zero field (ZFC) and in field (FC). The markers show the measured data points and the black line shows the equilibrium susceptibility calculated for the entire asymmetric unit using ORCA under a 1000 G applied field.

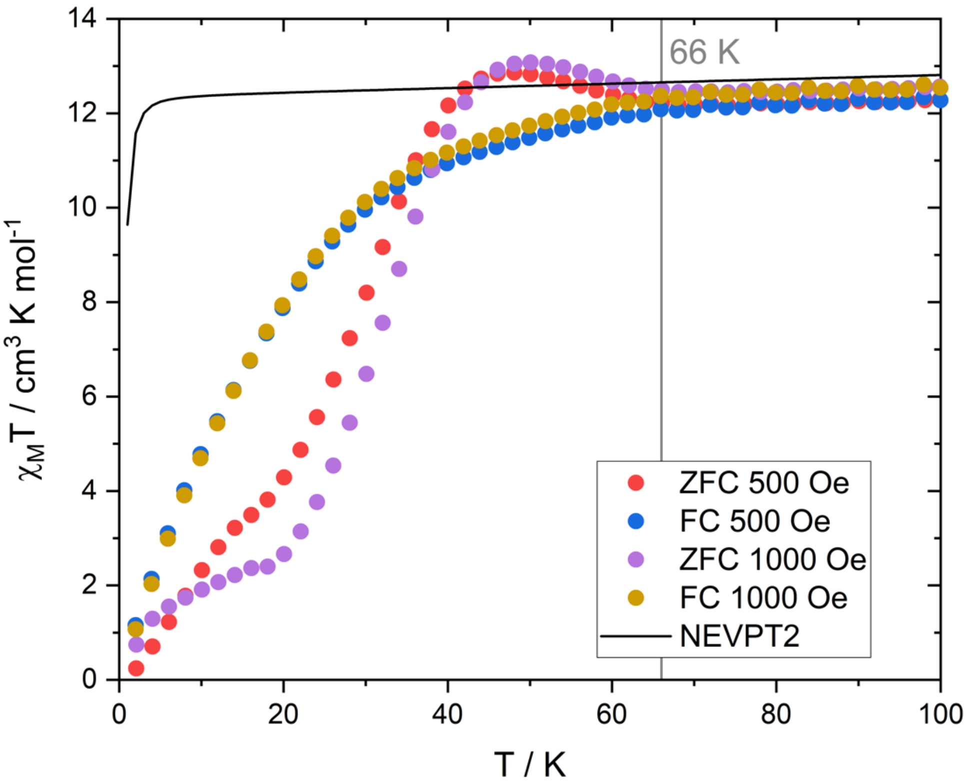

Figure S24. Plot of $\chi_M T$ vs T for **1** measured at 500 or 1000 Oe on warming after cooling in zero field (ZFC) and in field (FC). The markers show the measured data points and the black line shows the equilibrium $\chi_M T$ calculated for the entire asymmetric unit using ORCA under a 1000 G applied field.

Dc magnetisation decay measurements were measured at constant temperature by applying a 30 kOe magnetic field for 5 minutes to saturate the sample and then rapidly removing the field (700 Oe s^{-1}) and measuring the magnetic moment as a function of time as soon as the field reached zero. The decay was measured for at least ten half-lives (10 τ) at each temperature. Dc decay data were fit to a stretched exponential curve (Equation S1).

Equation S1: $$M(t) = M_0 + (M_1 - M_0)\exp\left(-(t/\tau)^{\beta}\right)$$

Where M_1 is the initial magnetisation once the field has been removed (fixed to the value at t = 0), M_0 is the residual magnetisation at infinite time (non-zero due to an imperfect zero-field condition), τ is the relaxation time in seconds and β is the stretching factor (Table S16, Figures S25-S27). The β values indicate a widening distribution of relaxation times as temperature decreases. Datasets with negative M_1 values or < 10 data points before reaching equilibrium were discarded.

Table S16. Best fit parameters for the magnetisation decay curves to the stretched exponential function.

T / K	M_0 / emu	M_1 / emu	M_1^{err} / emu	τ / s	τ^{err} / s	β	β^{err}	R^2	Total time/τ
5.00	0.40607	5.8E-04	6.1E-05	1543.5	1.1	0.6605	5E-04	0.99982	11.5
6.50	0.37299	1.1E-03	1.1E-04	1403.7	1.6	0.6694	8E-04	0.99973	12.2
8.00	0.35919	4.2E-04	7.3E-05	1353.2	1.3	0.6723	7E-04	0.99974	10.9
10.00	0.34396	-9.7E-05	7.0E-05	1291.9	1.3	0.6816	7E-04	0.99976	10.7
13.00	0.33118	-6.4E-05	6.6E-05	1166.5	1.1	0.7100	8E-04	0.99979	10.2
15.00	0.30129	1.4E-04	5.8E-05	1088.9	1.1	0.7434	9E-04	0.99975	10.7
17.00	0.27992	7.1E-06	4.3E-05	945.6	0.7	0.7519	7E-04	0.99987	10.4
20.00	0.24598	1.3E-04	3.7E-05	807.2	0.7	0.8005	8E-04	0.99986	10.6
23.00	0.2187	1.2E-04	3.1E-05	665.8	0.5	0.8372	8E-04	0.99990	10.3
26.00	0.20572	6.7E-05	2.6E-05	542.9	0.4	0.8645	8E-04	0.99993	10.4
29.00	0.18016	2.9E-05	2.2E-05	448.5	0.3	0.8921	8E-04	0.99995	10.3
32.00	0.16679	-9.2E-06	1.8E-05	365.5	0.2	0.9062	7E-04	0.99996	10.4
36.00	0.13911	-3.3E-05	1.5E-05	286.8	0.2	0.9276	8E-04	0.99997	10.3
39.00	0.12956	-5.6E-05	1.2E-05	236.7	0.1	0.9308	7E-04	0.99998	10.1
42.00	0.11586	-5.8E-05	1.3E-05	199.0	0.1	0.9416	9E-04	0.99997	10.6
46.00	0.10269	-6.2E-05	1.4E-05	156.8	0.1	0.949	1E-03	0.99997	10.8
50.00	0.08369	-6.2E-05	1.7E-05	121.1	0.1	0.957	2E-03	0.99995	10.5
54.00	0.05739	-7.7E-05	2.2E-05	85.3	0.2	0.964	3E-03	0.99986	11.7
57.00	0.04583	-8.4E-05	3.9E-05	54.6	0.3	0.954	8E-03	0.99946	13.1

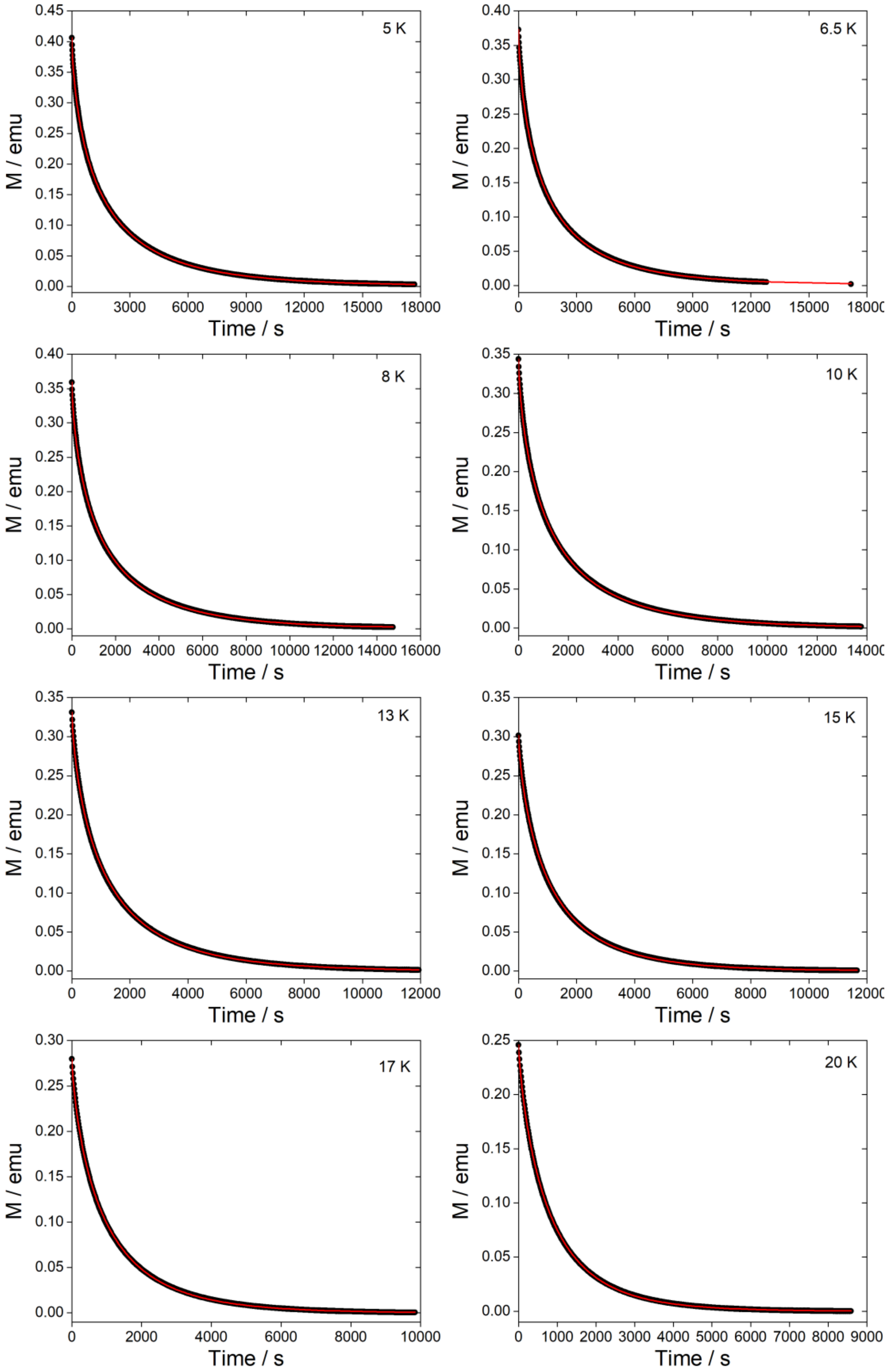

Figure S25. Magnetisation decay curves for **1** at 5, 6.5, 8, 10, 13, 15, 17 and 20 K. The markers show the measured data points and the red lines show fits to a stretched exponential curve.

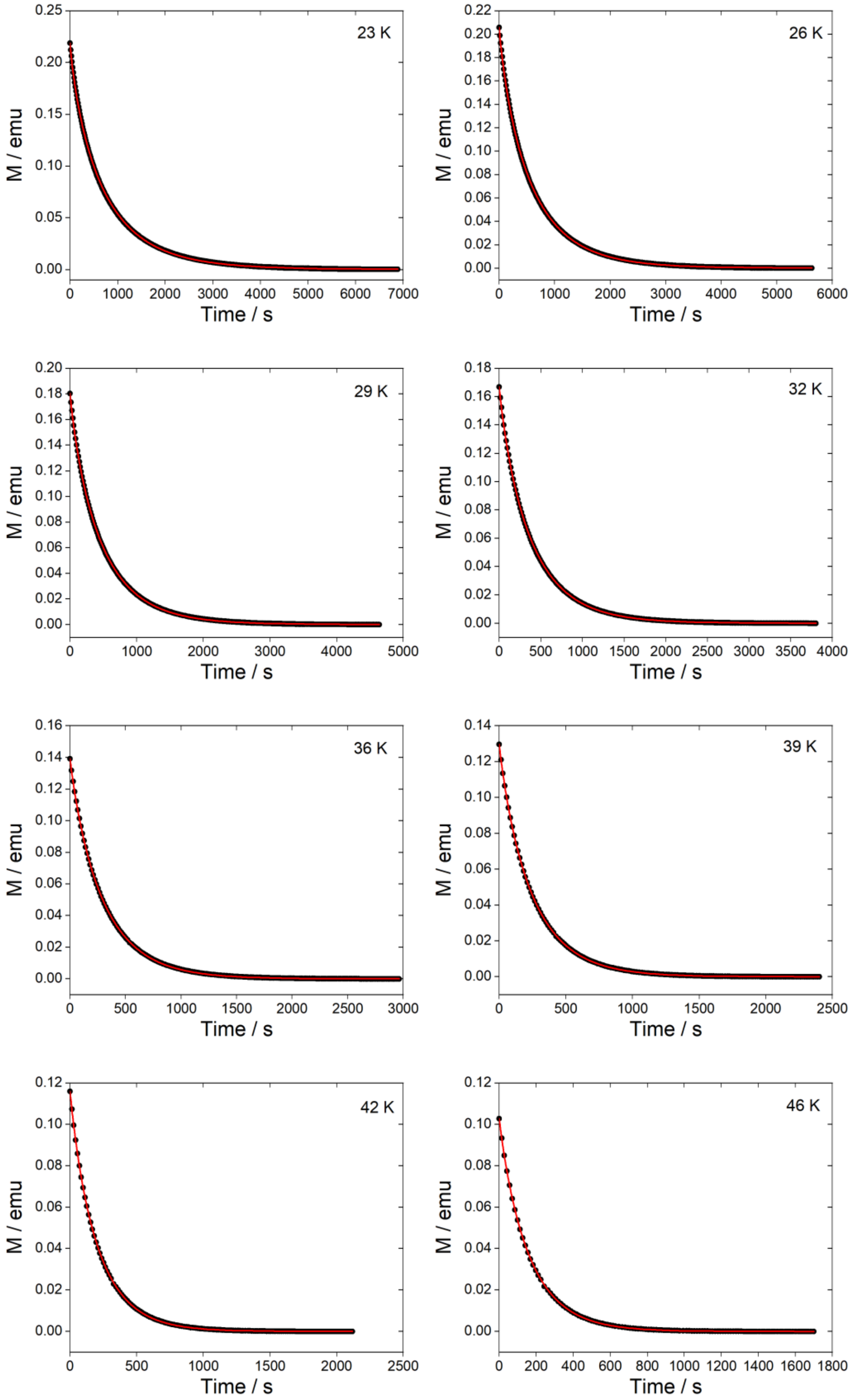

Figure S26. Magnetisation decay curves for **1** at 23, 26, 29, 32, 36, 39, 42 and 46 K. The markers show the measured data points and the red lines show fits to a stretched exponential curve.

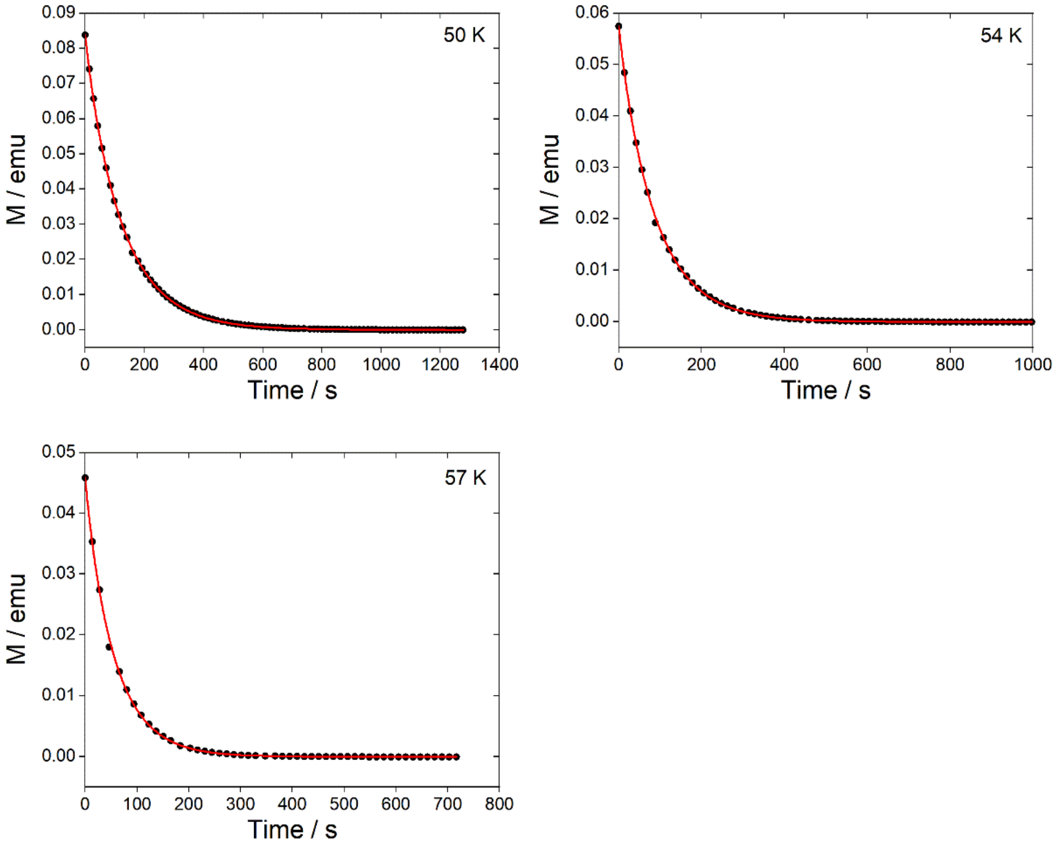

Figure S27. Magnetisation decay curves for **1** at 50, 54 and 57 K. The markers show the measured data points and the red lines show fits to a stretched exponential curve.

Ac susceptibility measurements were performed on the Quantum Design MPM3 with an oscillating field of 2 Oe. Measurements were recorded at temperatures between 70 and 107 K and for 21 frequencies between 0.1 and 1000 Hz. Data was analysed in CC-FIT2,[3] fitting the χ′ vs χ" Cole-Cole plot to the generalised Debye model to obtain relaxation times (τ) and distribution of relaxation times (α) (Table S17; Figures S28-S29). The α-values reveal a very narrow distribution of relaxation times and no significant temperature dependence.

Table S17. Best fit parameters for the ac magnetisation measurements to the generalised Debye model for **1**.

T / K	τ / s	$\tau_{LN}^{err\_upper}$/ s	$\tau_{LN}^{err\_lower}$/ s	τ_{Debye}^{err}/ s	χ_S	χ_S^{err}	χ_T	χ_T^{err}	α	α^{err}
70.00	1.51	2.28	1.00	3.52E-02	1.97E-02	2.13E-04	0.180	2.70E-03	4.67E-02	7.74E-03
72.50	0.645	0.645	0.645	5.20E-03	1.93E-02	1.80E-04	0.164	8.61E-04	8.03E-08[a]	4.57E-03
75.00	0.318	0.413	0.244	3.54E-03	1.87E-02	3.15E-04	0.159	9.53E-04	1.99E-02	6.70E-03
77.50	0.162	0.172	0.152	1.64E-03	1.81E-02	3.40E-04	0.154	7.25E-04	1.21E-03	6.41E-03
80.00	8.10E-02	9.07E-02	7.24E-02	8.50E-04	1.81E-02	3.87E-04	0.153	6.37E-04	3.82E-03	6.56E-03
82.73	4.02E-02	5.08E-02	3.17E-02	3.30E-04	1.75E-02	3.17E-04	0.149	4.17E-04	1.62E-02	5.01E-03
85.45	2.04E-02	2.42E-02	1.72E-02	1.51E-04	1.74E-02	2.99E-04	0.144	3.79E-04	8.63E-03	4.62E-03
88.18	1.06E-02	1.13E-02	1.00E-02	1.10E-04	1.67E-02	4.53E-04	0.138	4.30E-04	1.08E-03	6.46E-03
90.91	5.66E-03	5.66E-03	5.66E-03	9.27E-05	1.66E-02	7.73E-04	0.134	5.71E-04	8.36E-13[a]	1.02E-02
93.64	3.18E-03	3.75E-03	2.70E-03	4.80E-05	1.61E-02	7.65E-04	0.131	5.85E-04	8.03E-03	9.63E-03
96.36	1.78E-03	2.12E-03	1.50E-03	1.77E-05	1.53E-02	5.58E-04	0.126	3.00E-04	9.01E-03	6.26E-03
99.09	1.04E-03	1.18E-03	9.17E-04	5.38E-05	1.59E-02	3.22E-03	0.123	1.35E-03	4.68E-03	3.27E-02
101.82	6.10E-04	7.53E-04	4.94E-04	1.49E-05	1.42E-02	1.69E-03	0.119	5.96E-04	1.31E-02	1.50E-02
104.54	3.75E-04	4.46E-04	3.14E-04	5.74E-06	1.54E-02	1.15E-03	0.117	2.93E-04	9.08E-03	8.72E-03
107.27	2.27E-04	2.78E-04	1.86E-04	1.34E-05	1.47E-02	4.46E-03	0.113	5.52E-04	1.19E-02	2.60E-02

[a] An average value of 1.18E-02 was used to calculate errors in CC-FIT2.

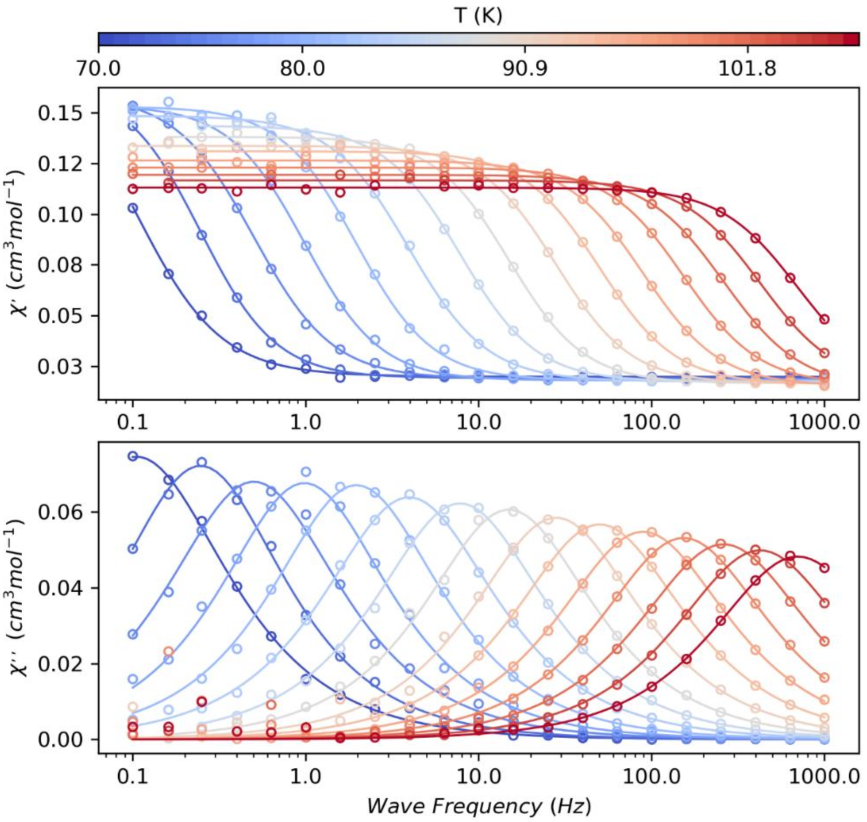

Figure S28. Fitting of the ac susceptibility data to a generalised Debye model in CC-FIT2[3] for **1.**

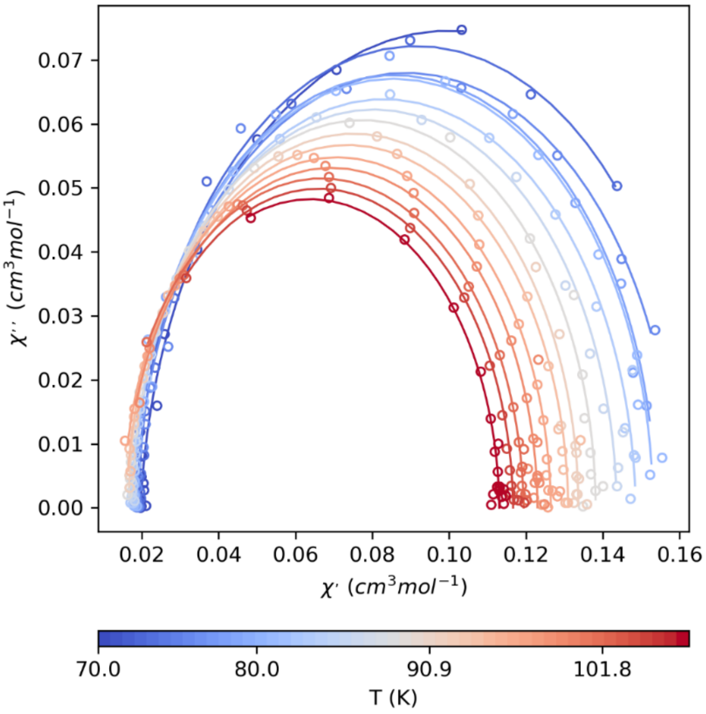

Figure S29. Cole-Cole plot showing the fitting of the ac data to a generalised Debye model in CC-FIT2[3] for **1.**

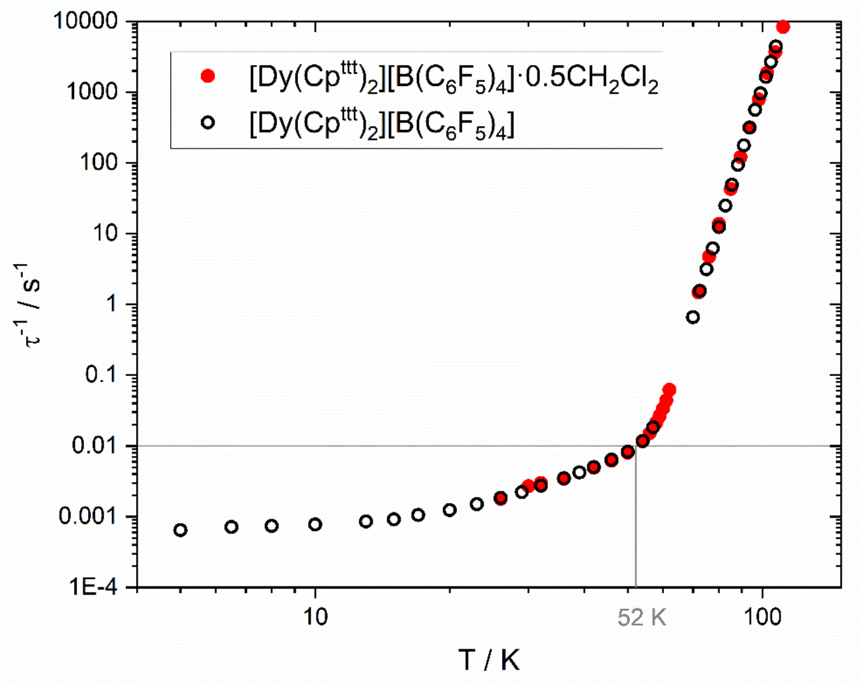

Figure S30. Comparison of the relaxation profile of **1** to the previously reported solvate, **1S**.[1]

The extracted relaxation times for **1** from the ac and dc magnetic data were fit in the program CC-FIT2, which uses the α and β values to assign errors to the relaxation rates. The α values for two temperatures were several orders of magnitude lower than at other temperatures (Table S17) and so were set to the average α value so as to not bias the data. The relaxation profile was fit to a combined Orbach, Raman and quantum tunnelling of magnetization (QTM) rate equation (Equation S2, Figure 4).

Equation S2: $\tau^{-1} = \tau_{QTM}^{-1} + \tau_{Raman}^{-1} + \tau_{Orbach}^{-1} = \tau_{QTM}^{-1} + CT^n + \tau_0^{-1}\exp\left(-U_{eff}/k_BT\right)$

The errors in the fitted parameters reflect the errors in the relaxation rates.

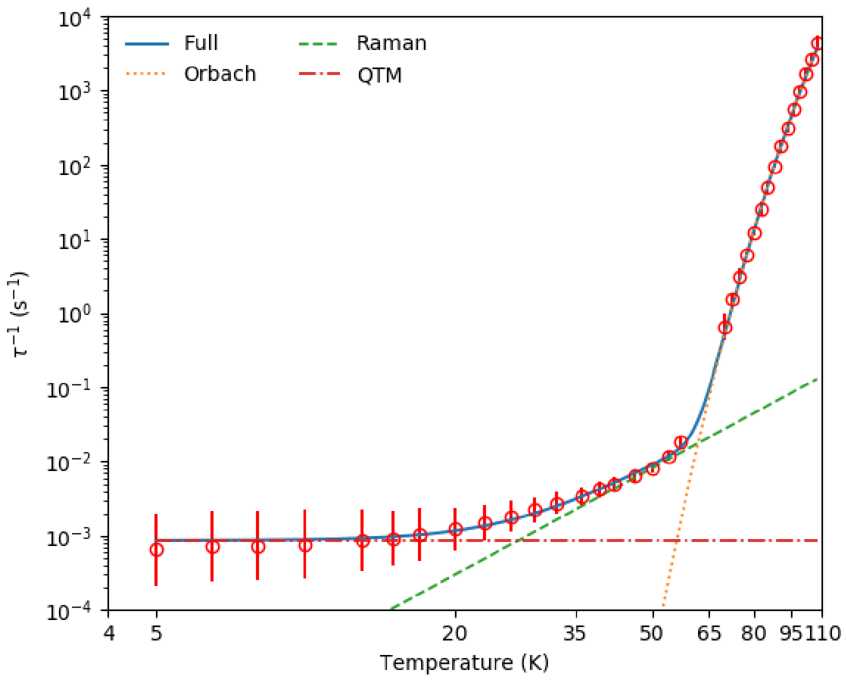

Figure S31. Temperature dependence of the relaxation rates, τ^{-1}, in **1**. The red circles are the relaxation rates extracted from ac susceptibility data (high temperature) and dc magnetization decay data (low temperature). The solid red lines are error bars from the distribution of relaxation times. The solid blue line shows the combined relaxation rate $\tau^{-1} = \tau_{\mathrm{QTM}}^{-1} + CT^{n} + \tau_0^{-1}\exp(-U_{\mathrm{eff}}/k_B T)$, the red dashed line shows the QTM rate τ_{QTM}^{-1}, the green dashed line shows the Raman relaxation rate CT^{n} and the orange dashed line shows the Orbach relaxation rate $\tau_0^{-1}\exp(-U_{\mathrm{eff}}/k_{\mathrm{B}}T)$ with the fitting parameters U_{eff} = 1240(20) cm^{-1}, τ_0 = $10^{-10.8(1)}$ s, C = $10^{-8(1)}$ s^{-1} K^{-n} with n = 3.6(6) and τ_{QTM}^{-1}= $10^{-3.1(2)}$ s^{-1}.

6. High pressure magnetic measurements

High-pressure variable-temperature variable-field (VTVF) magnetization data was measured using VSM on a QMD PPMS equipped with a 9 T magnet. 22.5 mg of ground **1** was loaded in a cut 6.5 x 2.6 mm Teflon tube inside a QMD BeCu magnetometry cell along with a piece of lead for pressure calibration and Fomblin Y oil as the PTM. Fomblin Y was used as PTM due to suspicions that the powder degrades in Daphne 7373. The magnetization versus field was measured from 5 K to 50 K with varying fields of 7 to -7 T with sweep rates of 22 Oe/s from 0 to 1 T, 54 Oe/s from 1 to 2 T and 91 Oe/s from 2 to 7 T. This results in an average sweep rate of ≈ 76 Oe/S, but with slower sweep rate at low fields. A diamagnetic correction of $0.5 \cdot 10^{-6} \cdot M$ of the sample mass was used for calculating the magnetization. The pressure was determined by sweeping the temperature from 6.7 to 7.3 K with an applied magnetic field of 20 Oe, as shown in Figure S32, to obtain the T_C, which was used to calculate the pressure according to:

$$P = \Delta T_C \cdot \left(\frac{dT_C}{dP}\right)^{-1}$$

where ΔT_C is the change in T_C and $\frac{dT_C}{dP}$ is tabulated as approximately -0.379 K/GPa. The T_C for Pb under conditions is approximately 7.19 K. The T_C of 6.78 K shown in Figure S32 indicates in a pressure of 1.11 GPa. The compression of the cell resulted in a change in length of around 1.2 mm, which should typically lead to a pressure of ≈1 GPa, although this depends on the length and filling of the Teflon tube.

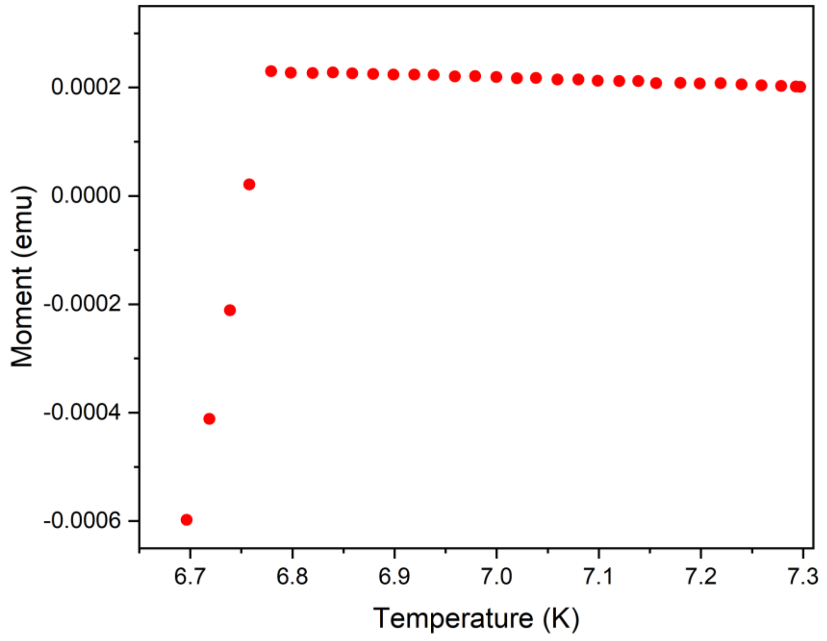

Figure S32. Measurement for determining the critical temperature of lead for pressure calibration. A T_C of 6.78 K is determined, leading to a pressure of around 1 GPa.

When filling the BeCu cell, the sample was weighed as 22.5 mg. However, this value appears to be highly overestimated. Firstly, the sample was weighed as it was being filled into the heavy BeCu cell inside the glovebox. Secondly, **1** is highly air- and light/heat-sensitive, and some crystals were seen to have decomposed *via* clear color changes. Thirdly, the crystals crushed for the measurement were coated in Fomblin Y oil. Thus, the weighed sample mass is expected be higher than the actual sample mass. It was also later found that actually fitting 22.5 mg sample into the Teflon sample chamber was unrealistic. To ensure that the overestimated mass was not due to the background signal from the BeCu cell, data was measured on an empty BeCu cell with a 2.6 x 6.5 mm Teflon tube filled with only Fomblin Y oil in magnetic fields of -7 to 7 T with a 100 Oe/s sweep rate and at temperatures from 10 to 2 K in steps of 1 K. This clearly showed that the background moments are much smaller than those measured for the sample (e.g. the measured background moment constitutes 0.3 % of the measured sample moment at 10 K and 7 T), and thus the background signal alone cannot be explain the overestimated sample mass.

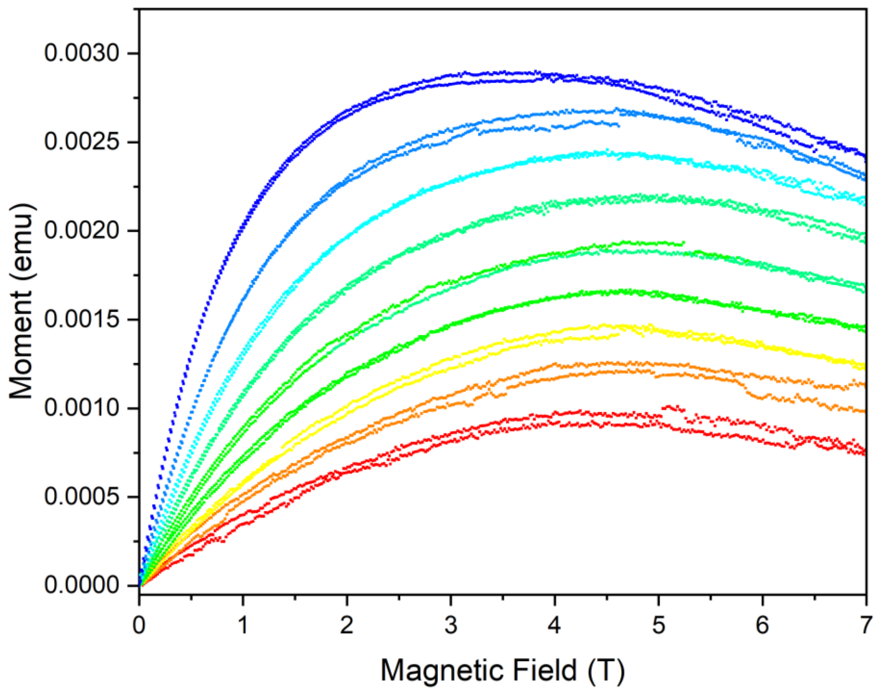

Figure S33. Background measurements on an empty BeCu cell between 2 K (blue) and 10 K (red).

High-pressure magnetic decay data was measured on **1** using a QMD PPMS in VSM mode with a QMD BeCu cell at temperatures from 8 K to 68 K in steps of 2-4 K. 11.49 mg of ground sample was loaded into a cut Teflon tube of size 5 x 2.6 mm inside the cell along with a piece of lead for pressure calibration and Fomblin Y oil as the PTM. The decay data shown here has not been subject to background correction. All decay measurements were carried out by first setting the field to 1000 Oe and waiting 600 s, setting the field to 0 with a 175 Oe/s sweep rate using driven mode (no overshoot), and then changing to persistent mode after 600 s to save helium.

The pressure was determined by measuring the magnetization at temperatures from 6.5 to 7.3 K with a temperature rate 0.05 K/min (0.02 K steps) and an applied field of 20 Oe. The measured pressure calibrations can be found in Figure S34 and Table S18.

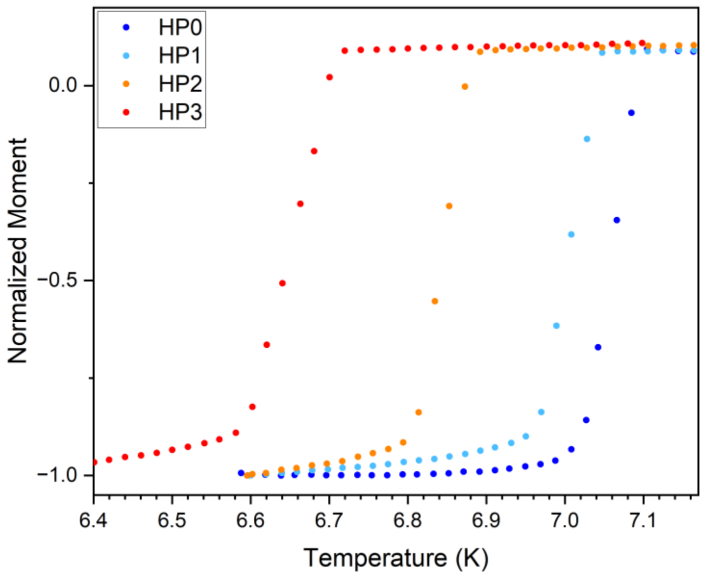

Figure S34. Pressure calibration measurements for determining the pressures of the high-pressure decay magnetization series on **1**: HP0 (blue), HP1 (light blue), HP2 (orange) and HP3 (red). The moments have been normalized to allow the series to be overlaid.

Table S18. T_c, cell length, cell compression and calculated pressures for the decay series on **1**. Measured using Pb as manometer with a 20 Oe applied field.

Pressure point	T_c (K)	Pressure (GPa)	Cell length (mm)	Cell compression (mm)
**HP0 (ambient)**	7.11	0	69.0	0
**HP1**	7.05	0.37	68.4	0.6
**HP2**	6.89	0.79	67.85	1.15
**HP3**	6.72	1.20	67.52	1.48

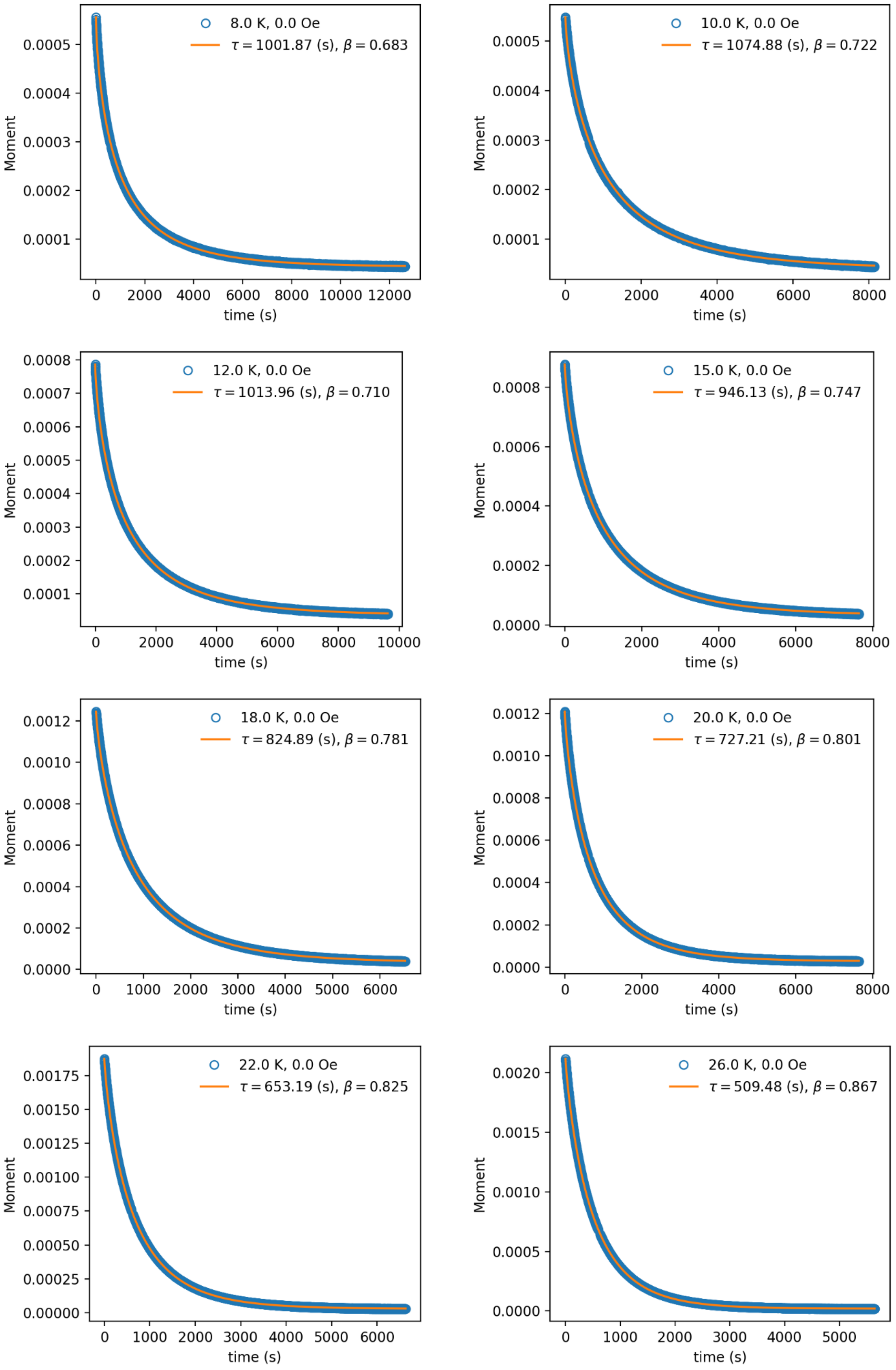
8.0 K, 0.0 Oe
$\tau = 1001.87$ (s), $\beta = 0.683$
10.0 K, 0.0 Oe
$\tau = 1074.88$ (s), $\beta = 0.722$
12.0 K, 0.0 Oe
$\tau = 1013.96$ (s), $\beta = 0.710$
15.0 K, 0.0 Oe
$\tau = 946.13$ (s), $\beta = 0.747$
18.0 K, 0.0 Oe
$\tau = 824.89$ (s), $\beta = 0.781$
20.0 K, 0.0 Oe
$\tau = 727.21$ (s), $\beta = 0.801$
22.0 K, 0.0 Oe
$\tau = 653.19$ (s), $\beta = 0.825$
26.0 K, 0.0 Oe
$\tau = 509.48$ (s), $\beta = 0.867$
Moment
time (s)

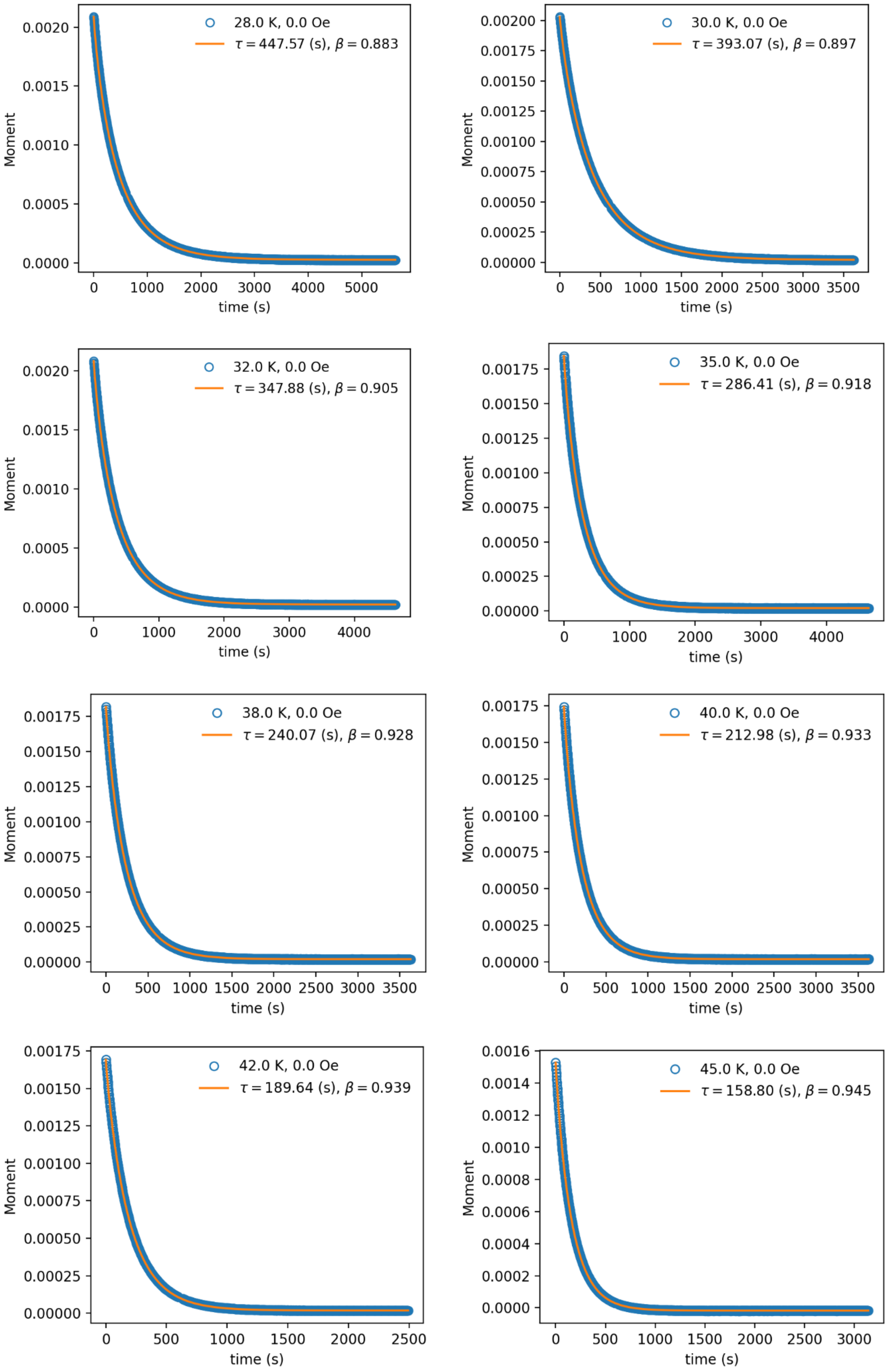
28.0 K, 0.0 Oe
τ = 447.57 (s), β = 0.883
30.0 K, 0.0 Oe
τ = 393.07 (s), β = 0.897
32.0 K, 0.0 Oe
τ = 347.88 (s), β = 0.905
35.0 K, 0.0 Oe
τ = 286.41 (s), β = 0.918
38.0 K, 0.0 Oe
τ = 240.07 (s), β = 0.928
40.0 K, 0.0 Oe
τ = 212.98 (s), β = 0.933
42.0 K, 0.0 Oe
τ = 189.64 (s), β = 0.939
45.0 K, 0.0 Oe
τ = 158.80 (s), β = 0.945
Moment
time (s)

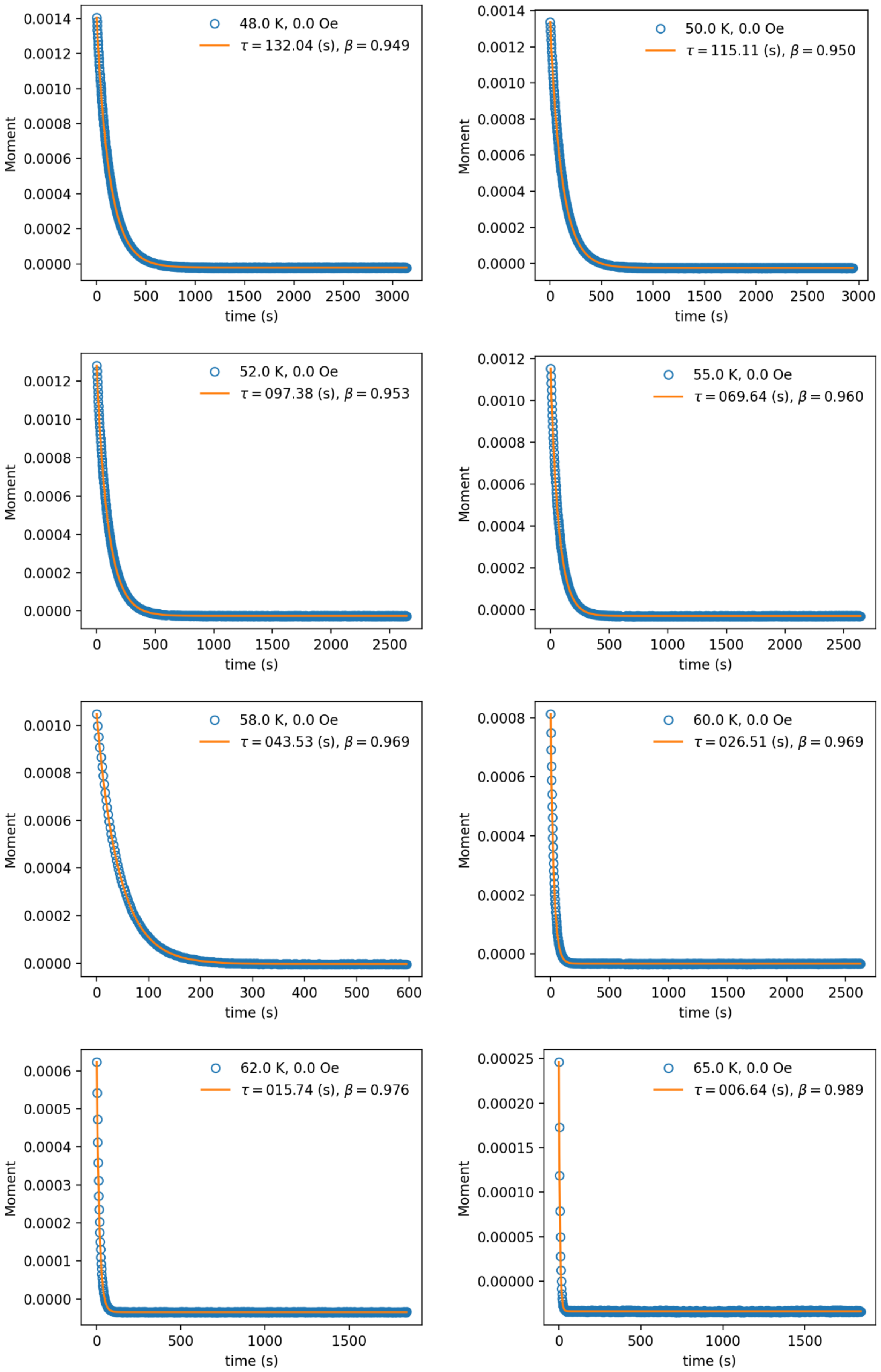
48.0 K, 0.0 Oe
$\tau = 132.04$ (s), $\beta = 0.949$
50.0 K, 0.0 Oe
$\tau = 115.11$ (s), $\beta = 0.950$
52.0 K, 0.0 Oe
$\tau = 097.38$ (s), $\beta = 0.953$
55.0 K, 0.0 Oe
$\tau = 069.64$ (s), $\beta = 0.960$
58.0 K, 0.0 Oe
$\tau = 043.53$ (s), $\beta = 0.969$
60.0 K, 0.0 Oe
$\tau = 026.51$ (s), $\beta = 0.969$
62.0 K, 0.0 Oe
$\tau = 015.74$ (s), $\beta = 0.976$
65.0 K, 0.0 Oe
$\tau = 006.64$ (s), $\beta = 0.989$
Moment
time (s)

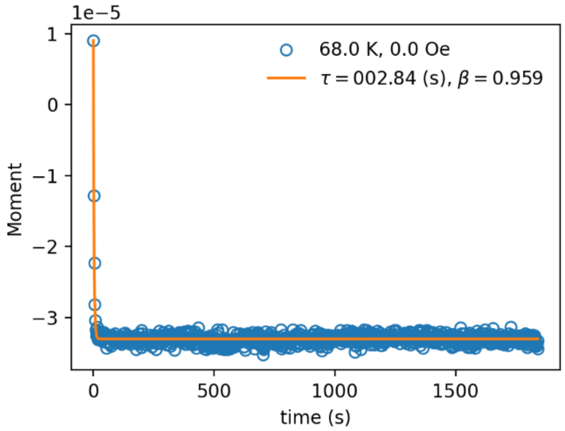

Figure S35. Magnetisation decays for **1** at ambient pressure inside the HP cell in the temperature range 8-68 K. The blue circles show the observed data points and the orange solid line shows the best fit to the relaxation time. Individual temperatures and fitted relaxation times are shown on the graphs.

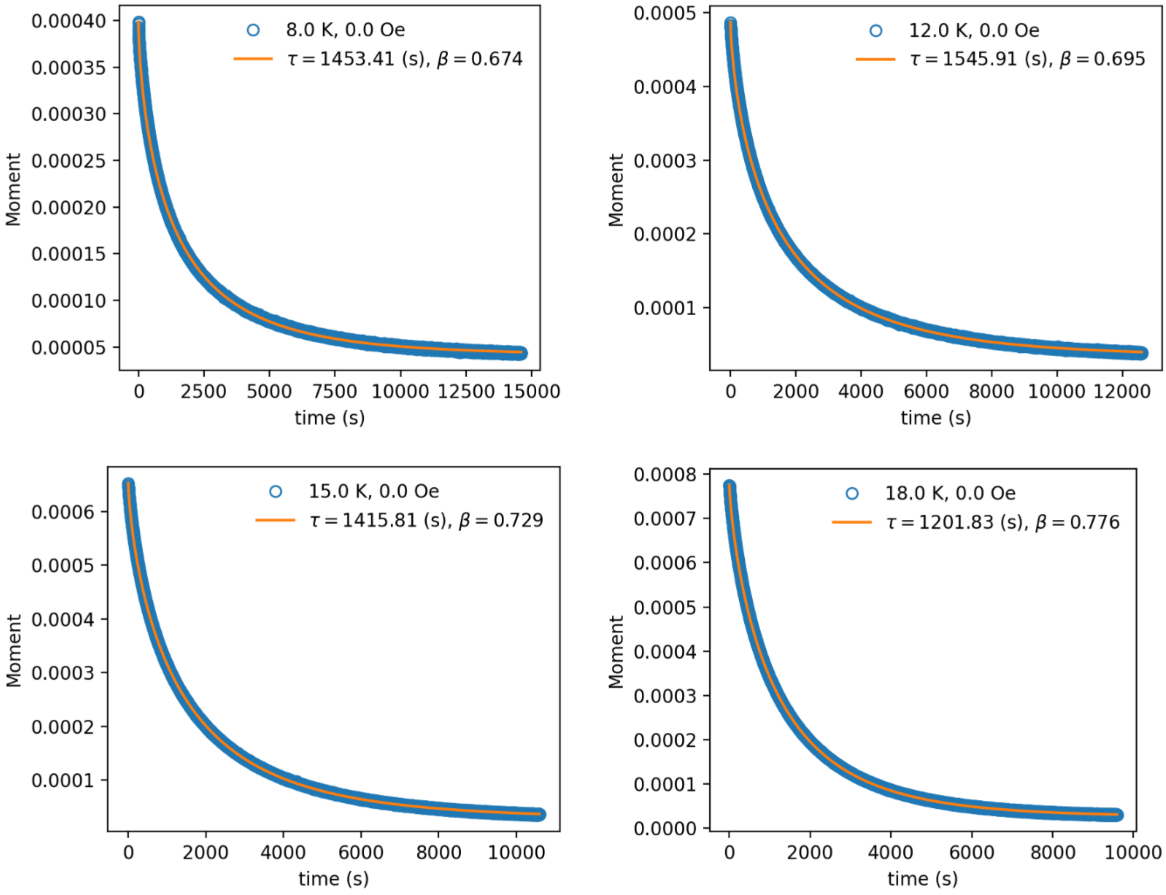

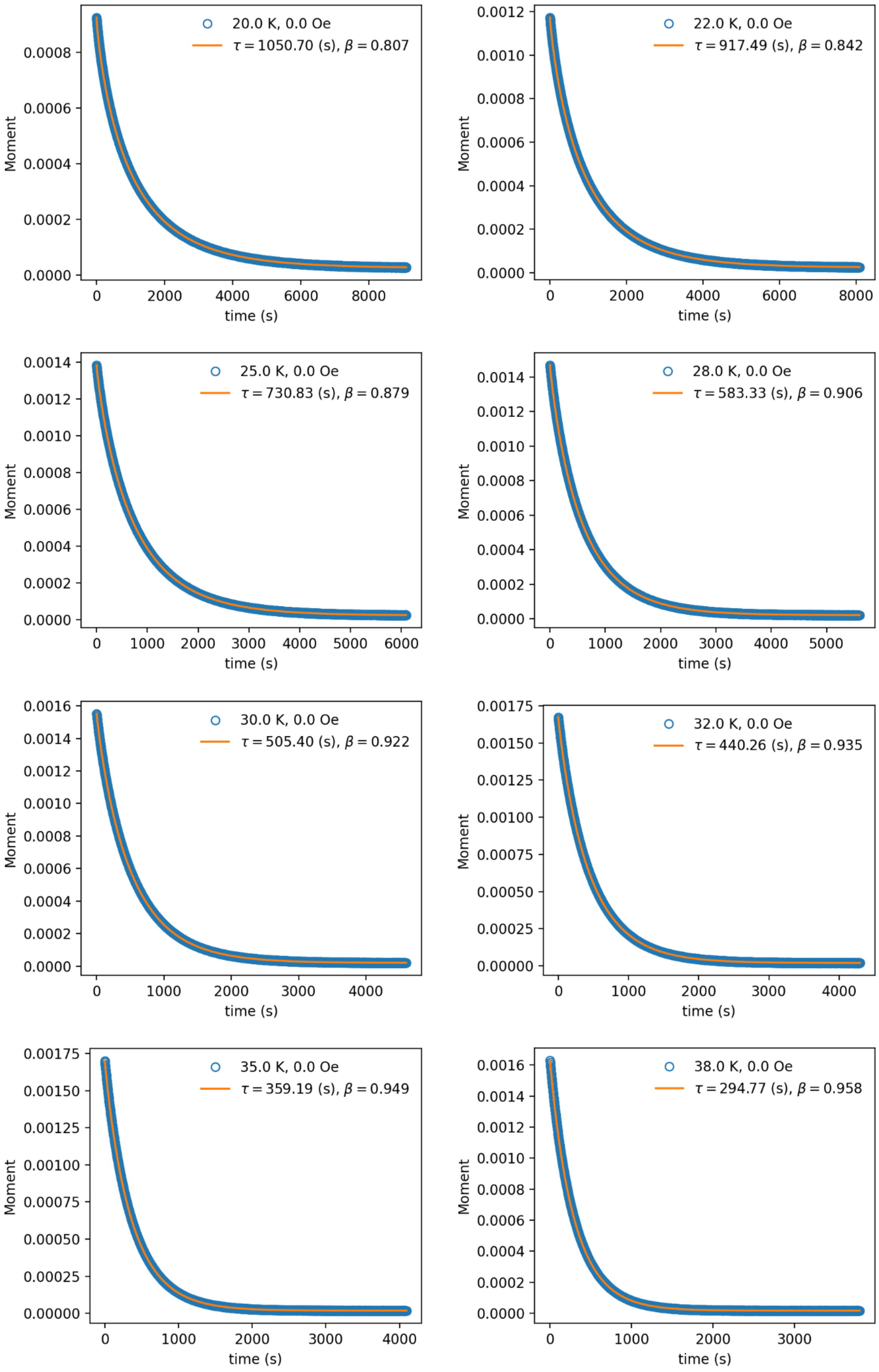
20.0 K, 0.0 Oe
$\tau = 1050.70$ (s), $\beta = 0.807$
22.0 K, 0.0 Oe
$\tau = 917.49$ (s), $\beta = 0.842$
25.0 K, 0.0 Oe
$\tau = 730.83$ (s), $\beta = 0.879$
28.0 K, 0.0 Oe
$\tau = 583.33$ (s), $\beta = 0.906$
30.0 K, 0.0 Oe
$\tau = 505.40$ (s), $\beta = 0.922$
32.0 K, 0.0 Oe
$\tau = 440.26$ (s), $\beta = 0.935$
35.0 K, 0.0 Oe
$\tau = 359.19$ (s), $\beta = 0.949$
38.0 K, 0.0 Oe
$\tau = 294.77$ (s), $\beta = 0.958$
Moment
time (s)

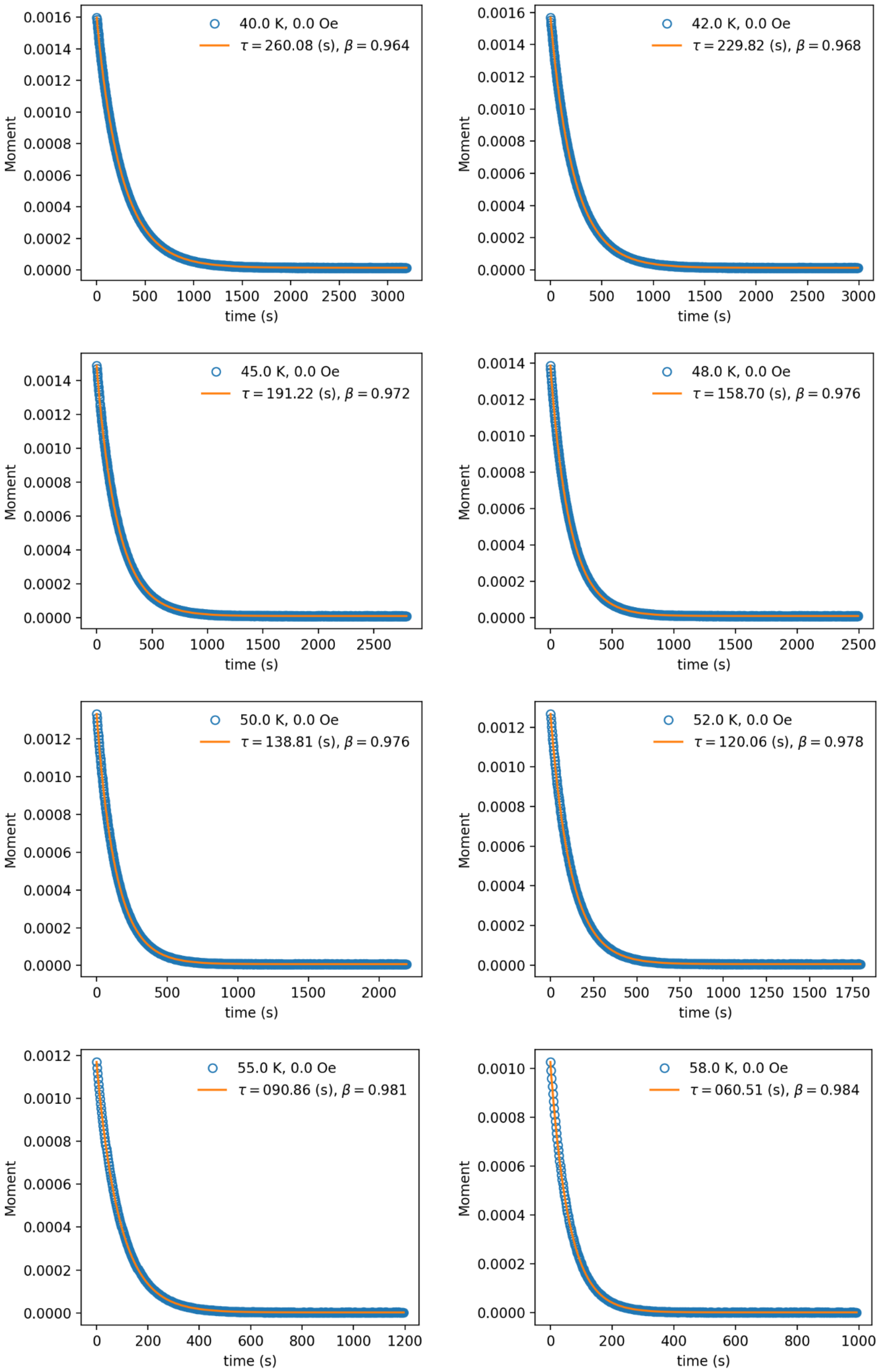
40.0 K, 0.0 Oe
τ = 260.08 (s), β = 0.964
42.0 K, 0.0 Oe
τ = 229.82 (s), β = 0.968
45.0 K, 0.0 Oe
τ = 191.22 (s), β = 0.972
48.0 K, 0.0 Oe
τ = 158.70 (s), β = 0.976
50.0 K, 0.0 Oe
τ = 138.81 (s), β = 0.976
52.0 K, 0.0 Oe
τ = 120.06 (s), β = 0.978
55.0 K, 0.0 Oe
τ = 090.86 (s), β = 0.981
58.0 K, 0.0 Oe
τ = 060.51 (s), β = 0.984
Moment
time (s)

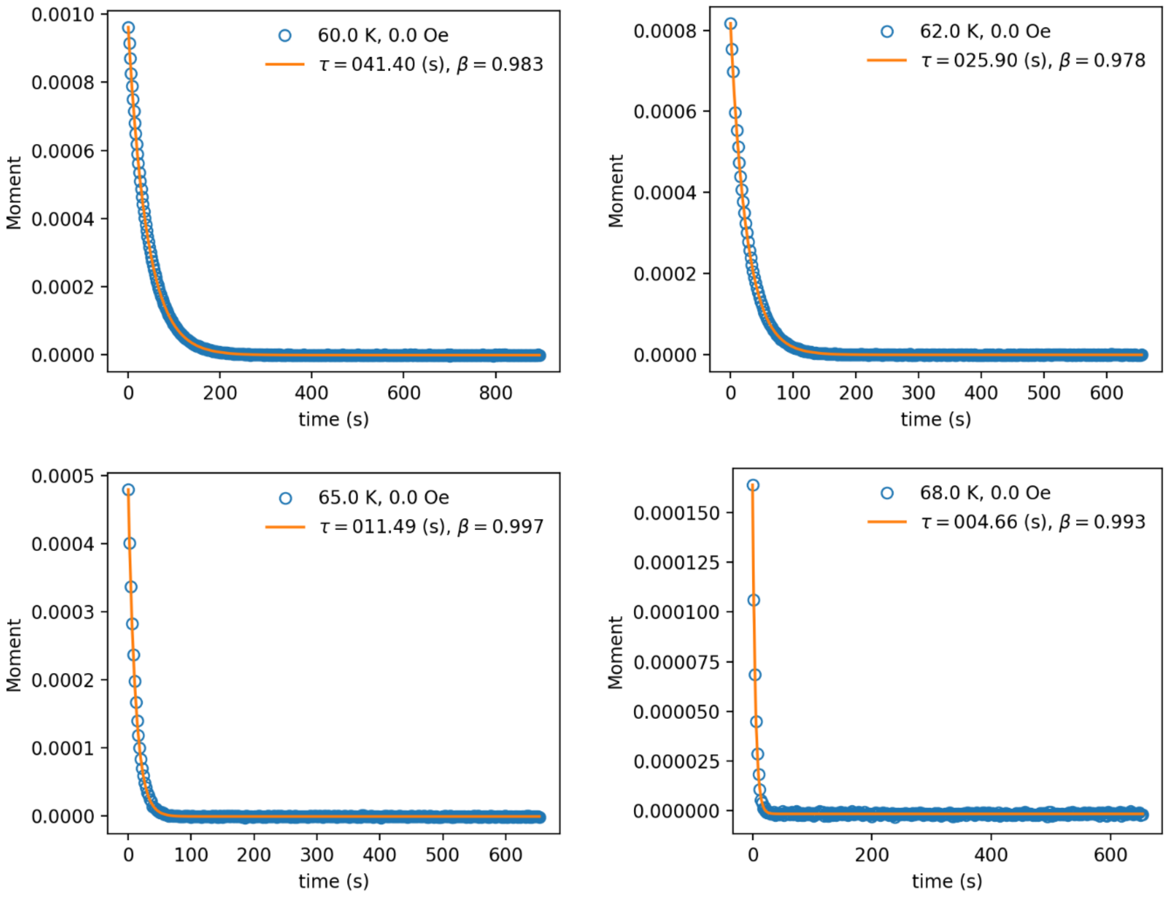

Figure S36. Magnetisation decays for **1** at HP1 (0.37 GPa) inside the HP cell in the temperature range 8-68 K. The blue circles show the observed data points and the orange solid line shows the best fit to the relaxation time. Individual temperatures and fitted relaxation times are shown on the graphs.

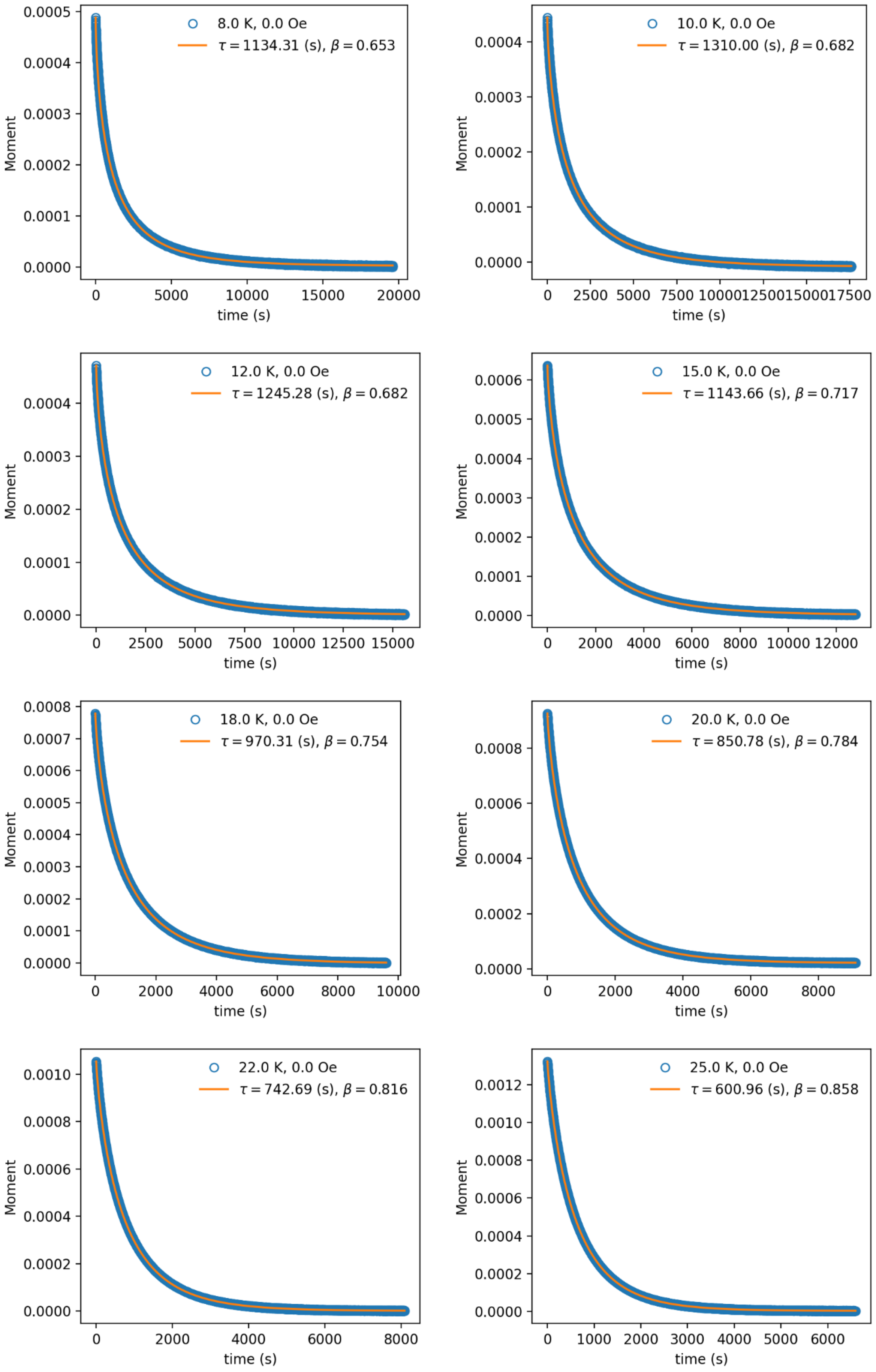
8.0 K, 0.0 Oe
τ = 1134.31 (s), β = 0.653
10.0 K, 0.0 Oe
τ = 1310.00 (s), β = 0.682
12.0 K, 0.0 Oe
τ = 1245.28 (s), β = 0.682
15.0 K, 0.0 Oe
τ = 1143.66 (s), β = 0.717
18.0 K, 0.0 Oe
τ = 970.31 (s), β = 0.754
20.0 K, 0.0 Oe
τ = 850.78 (s), β = 0.784
22.0 K, 0.0 Oe
τ = 742.69 (s), β = 0.816
25.0 K, 0.0 Oe
τ = 600.96 (s), β = 0.858
Moment
time (s)

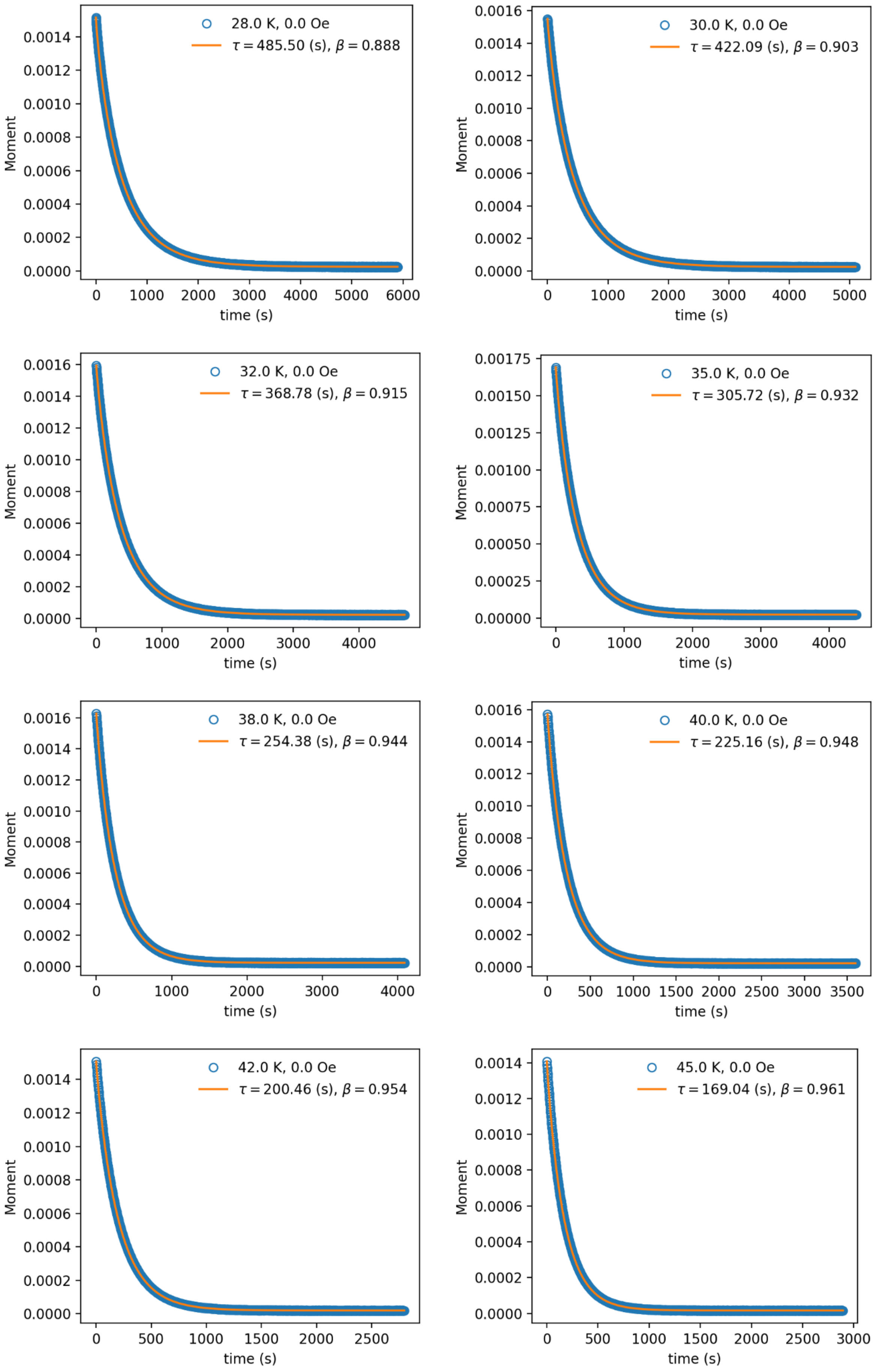
28.0 K, 0.0 Oe
$\tau = 485.50$ (s), $\beta = 0.888$
30.0 K, 0.0 Oe
$\tau = 422.09$ (s), $\beta = 0.903$
32.0 K, 0.0 Oe
$\tau = 368.78$ (s), $\beta = 0.915$
35.0 K, 0.0 Oe
$\tau = 305.72$ (s), $\beta = 0.932$
38.0 K, 0.0 Oe
$\tau = 254.38$ (s), $\beta = 0.944$
40.0 K, 0.0 Oe
$\tau = 225.16$ (s), $\beta = 0.948$
42.0 K, 0.0 Oe
$\tau = 200.46$ (s), $\beta = 0.954$
45.0 K, 0.0 Oe
$\tau = 169.04$ (s), $\beta = 0.961$
Moment
time (s)

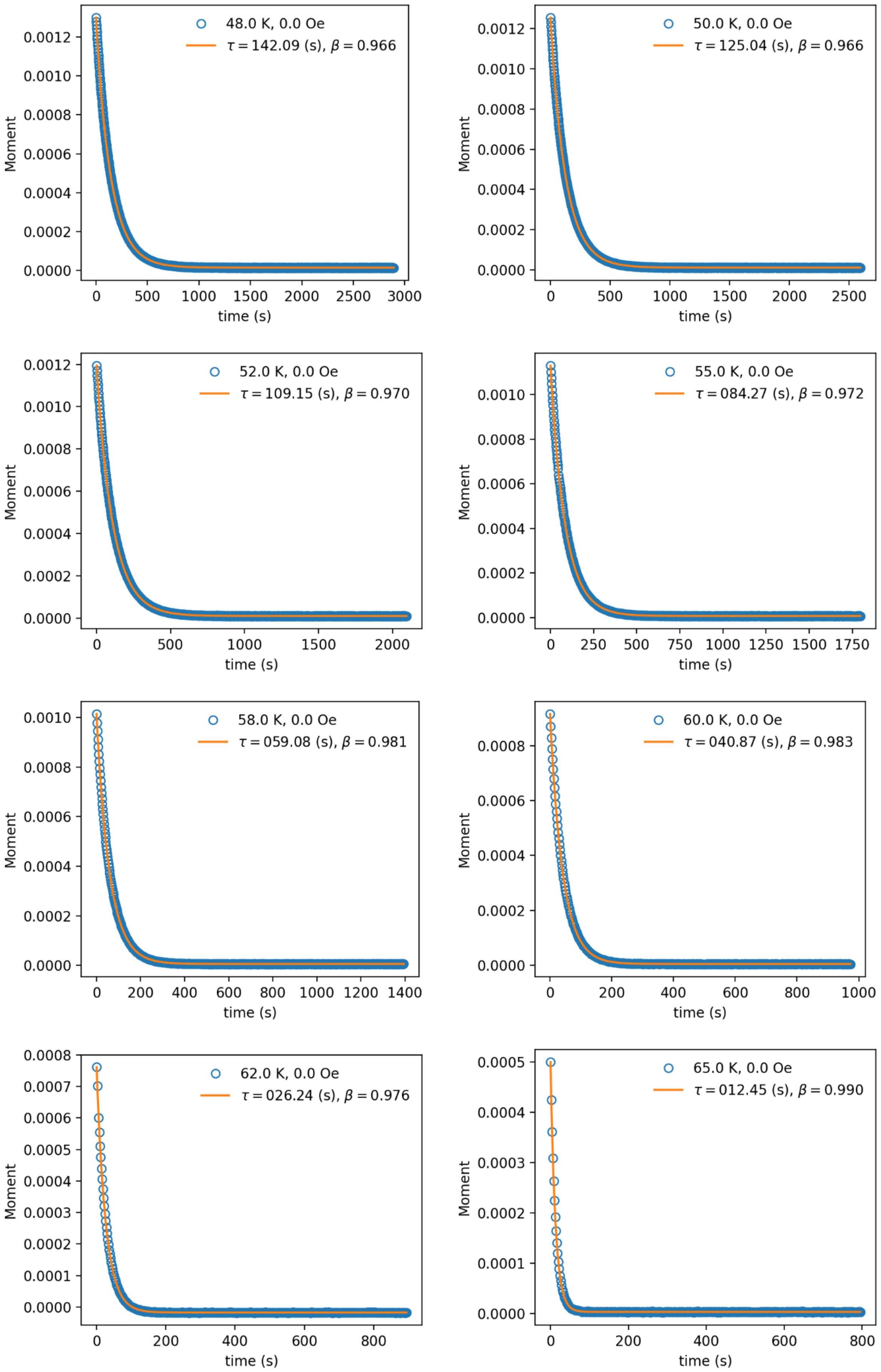
48.0 K, 0.0 Oe
τ = 142.09 (s), β = 0.966
50.0 K, 0.0 Oe
τ = 125.04 (s), β = 0.966
52.0 K, 0.0 Oe
τ = 109.15 (s), β = 0.970
55.0 K, 0.0 Oe
τ = 084.27 (s), β = 0.972
58.0 K, 0.0 Oe
τ = 059.08 (s), β = 0.981
60.0 K, 0.0 Oe
τ = 040.87 (s), β = 0.983
62.0 K, 0.0 Oe
τ = 026.24 (s), β = 0.976
65.0 K, 0.0 Oe
τ = 012.45 (s), β = 0.990
Moment
time (s)

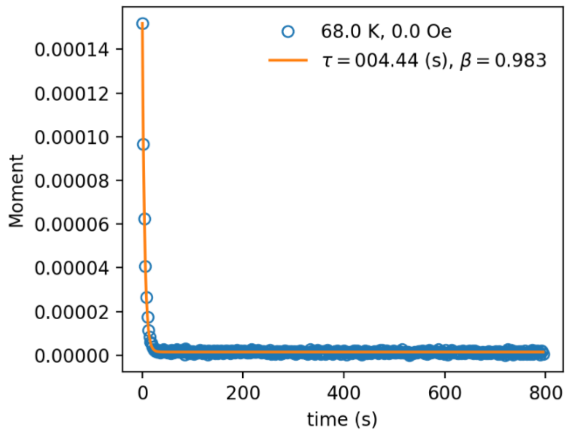

Figure S37. Magnetisation decays for **1** at HP2 (0.79 GPa) inside the HP cell in the temperature range 8-68 K. The blue circles show the observed data points and the orange solid line shows the best fit to the relaxation time. Individual temperatures and fitted relaxation times are shown on the graphs.

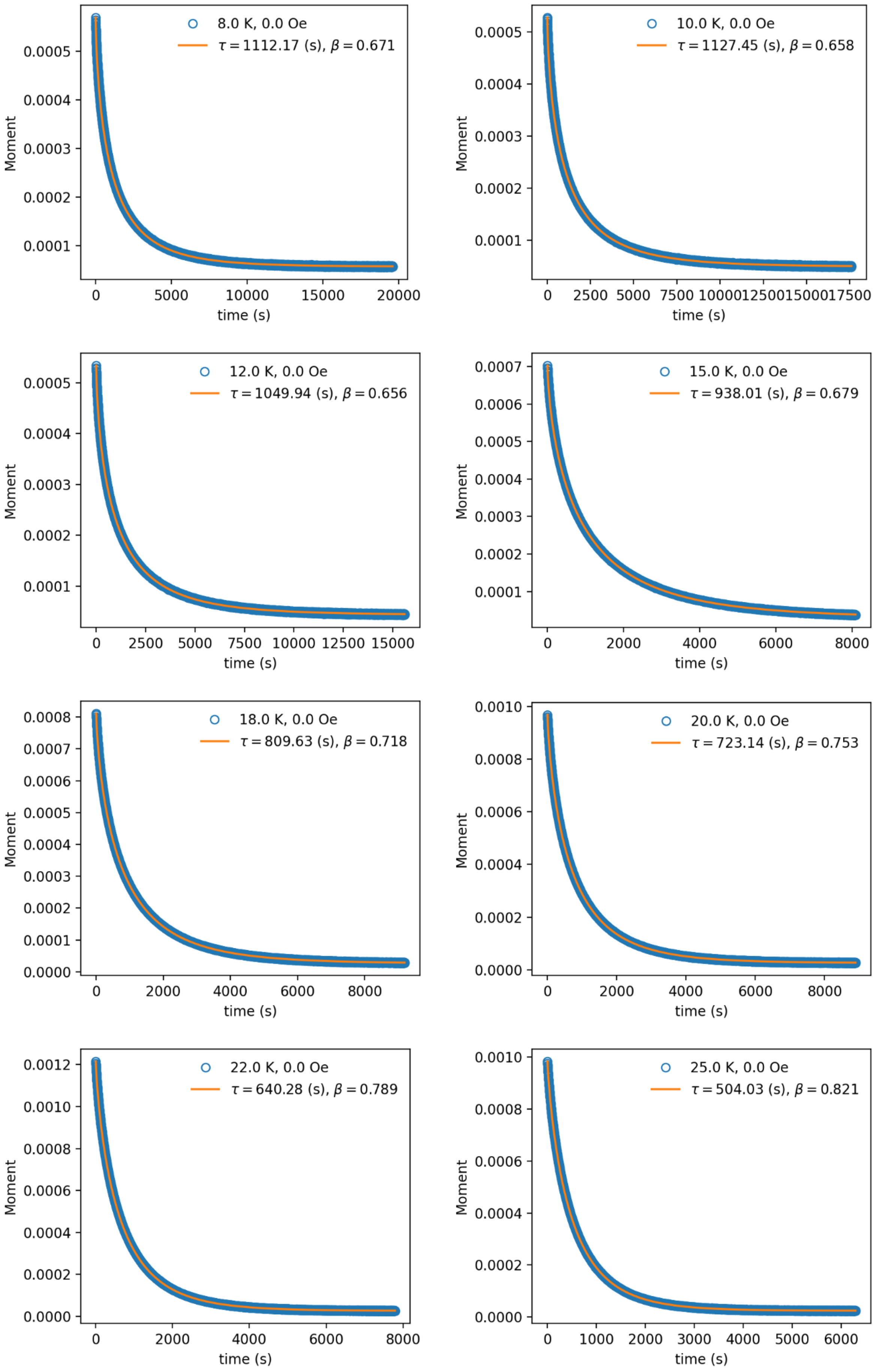
8.0 K, 0.0 Oe
$\tau = 1112.17$ (s), $\beta = 0.671$
10.0 K, 0.0 Oe
$\tau = 1127.45$ (s), $\beta = 0.658$
12.0 K, 0.0 Oe
$\tau = 1049.94$ (s), $\beta = 0.656$
15.0 K, 0.0 Oe
$\tau = 938.01$ (s), $\beta = 0.679$
18.0 K, 0.0 Oe
$\tau = 809.63$ (s), $\beta = 0.718$
20.0 K, 0.0 Oe
$\tau = 723.14$ (s), $\beta = 0.753$
22.0 K, 0.0 Oe
$\tau = 640.28$ (s), $\beta = 0.789$
25.0 K, 0.0 Oe
$\tau = 504.03$ (s), $\beta = 0.821$
Moment
time (s)

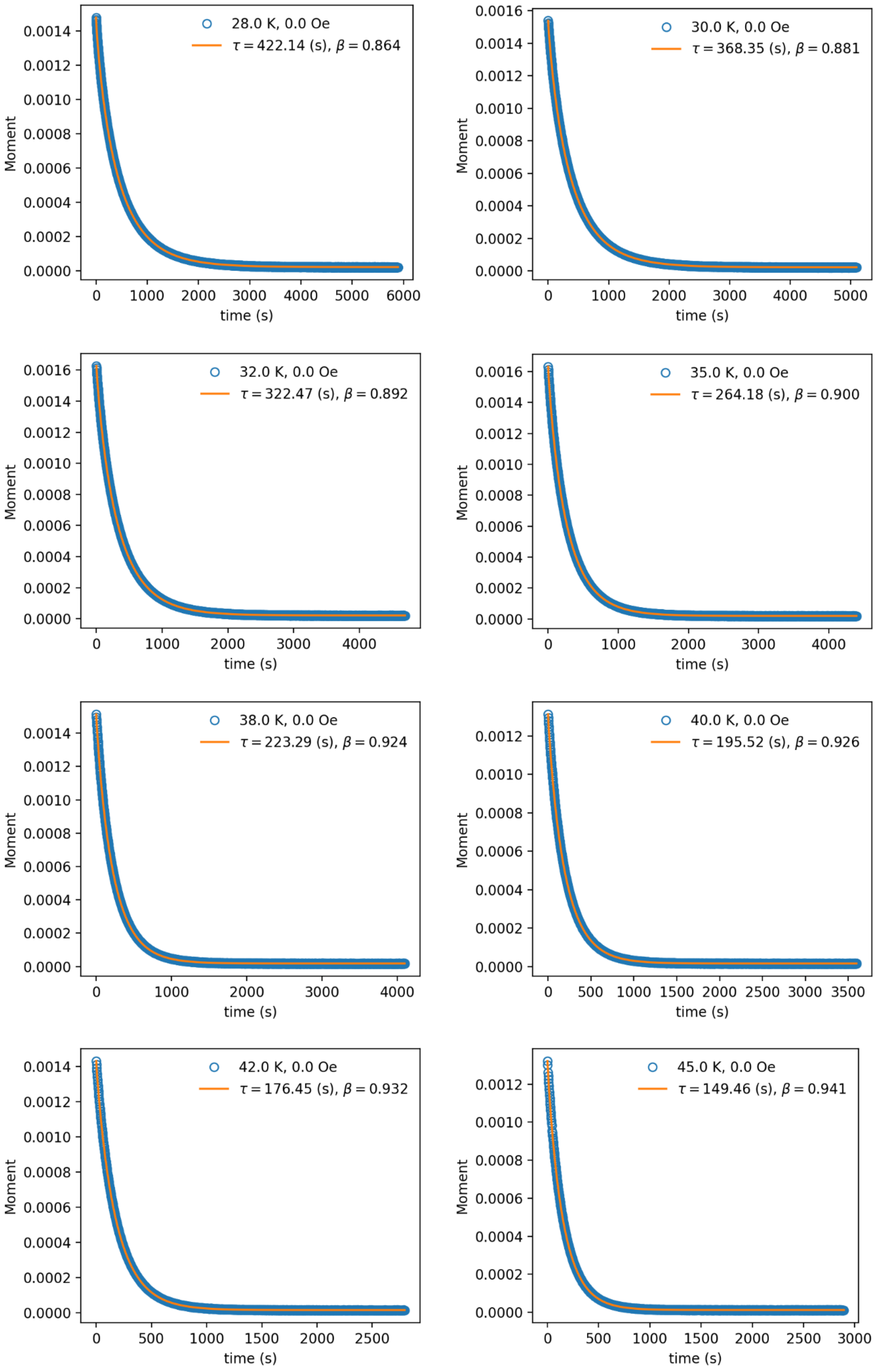
28.0 K, 0.0 Oe
τ = 422.14 (s), β = 0.864
30.0 K, 0.0 Oe
τ = 368.35 (s), β = 0.881
32.0 K, 0.0 Oe
τ = 322.47 (s), β = 0.892
35.0 K, 0.0 Oe
τ = 264.18 (s), β = 0.900
38.0 K, 0.0 Oe
τ = 223.29 (s), β = 0.924
40.0 K, 0.0 Oe
τ = 195.52 (s), β = 0.926
42.0 K, 0.0 Oe
τ = 176.45 (s), β = 0.932
45.0 K, 0.0 Oe
τ = 149.46 (s), β = 0.941
Moment
time (s)

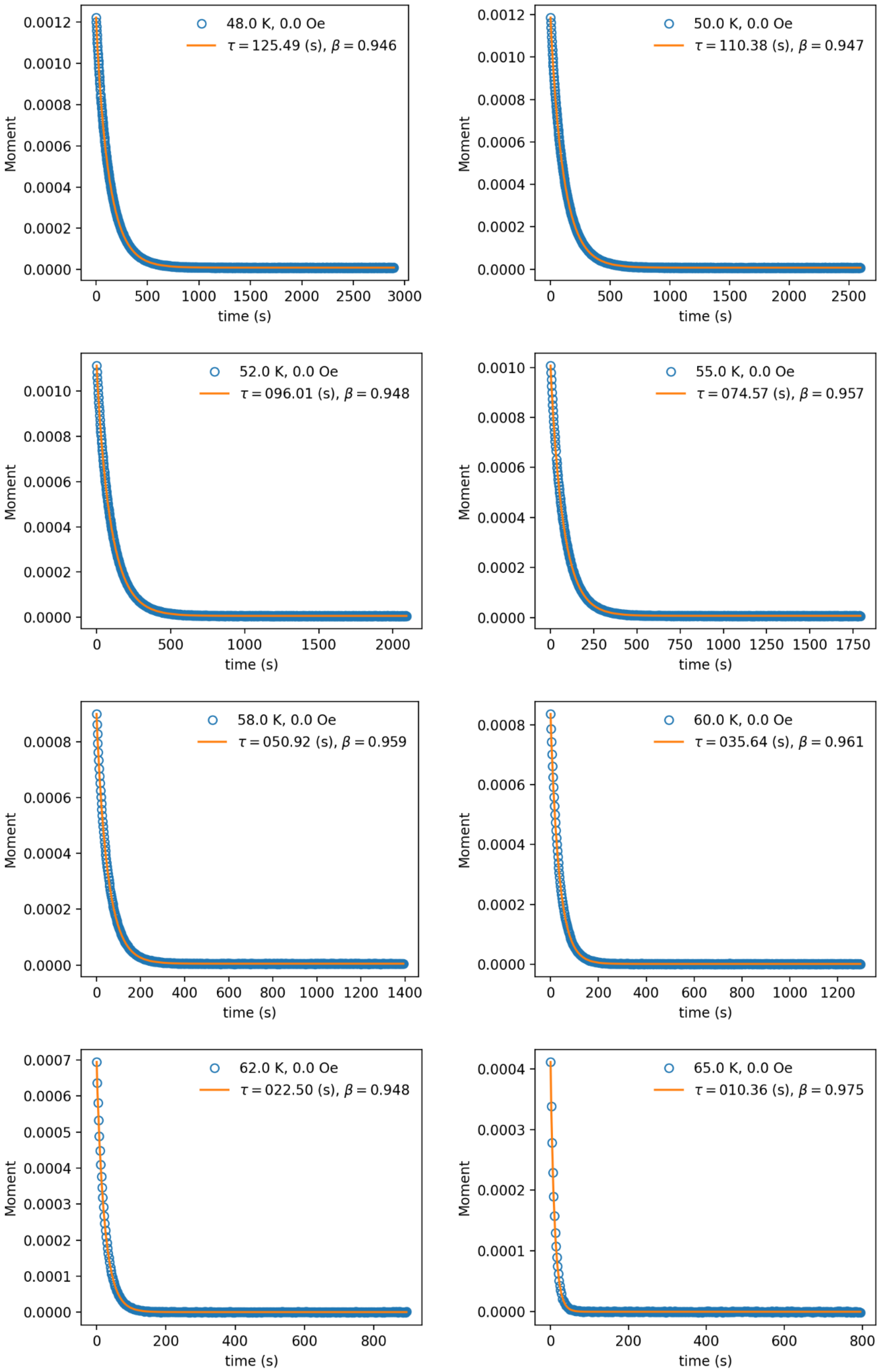
48.0 K, 0.0 Oe
τ = 125.49 (s), β = 0.946
50.0 K, 0.0 Oe
τ = 110.38 (s), β = 0.947
52.0 K, 0.0 Oe
τ = 096.01 (s), β = 0.948
55.0 K, 0.0 Oe
τ = 074.57 (s), β = 0.957
58.0 K, 0.0 Oe
τ = 050.92 (s), β = 0.959
60.0 K, 0.0 Oe
τ = 035.64 (s), β = 0.961
62.0 K, 0.0 Oe
τ = 022.50 (s), β = 0.948
65.0 K, 0.0 Oe
τ = 010.36 (s), β = 0.975
Moment
time (s)

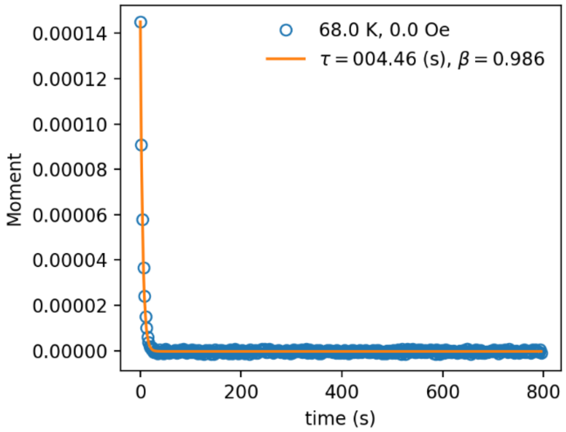

Figure S38. Magnetisation decays for **1** at HP3 (1.2 GPa) inside the HP cell in the temperature range 8-68 K. The blue circles show the observed data points and the orange solid line shows the best fit to the relaxation time. Individual temperatures and fitted relaxation times are shown on the graphs.

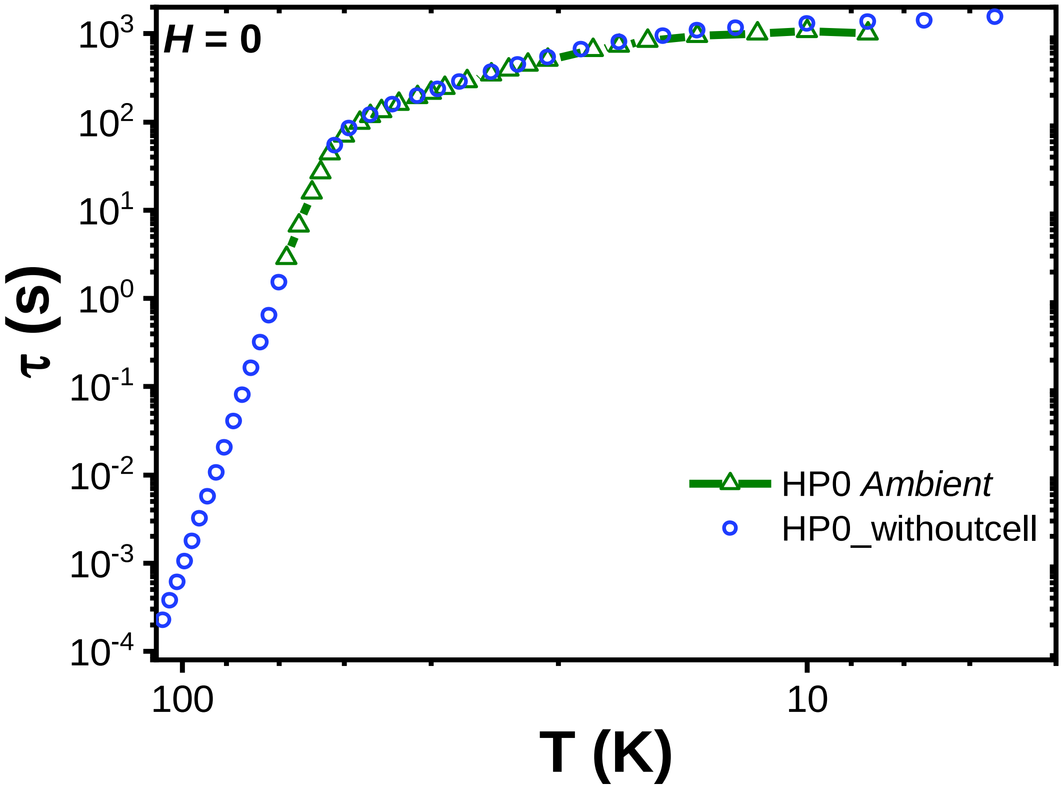

Figure S39. Relaxation profile (τ vs T) for **1** at ambient pressure with (green line) and without (blue circles) the HP cell.

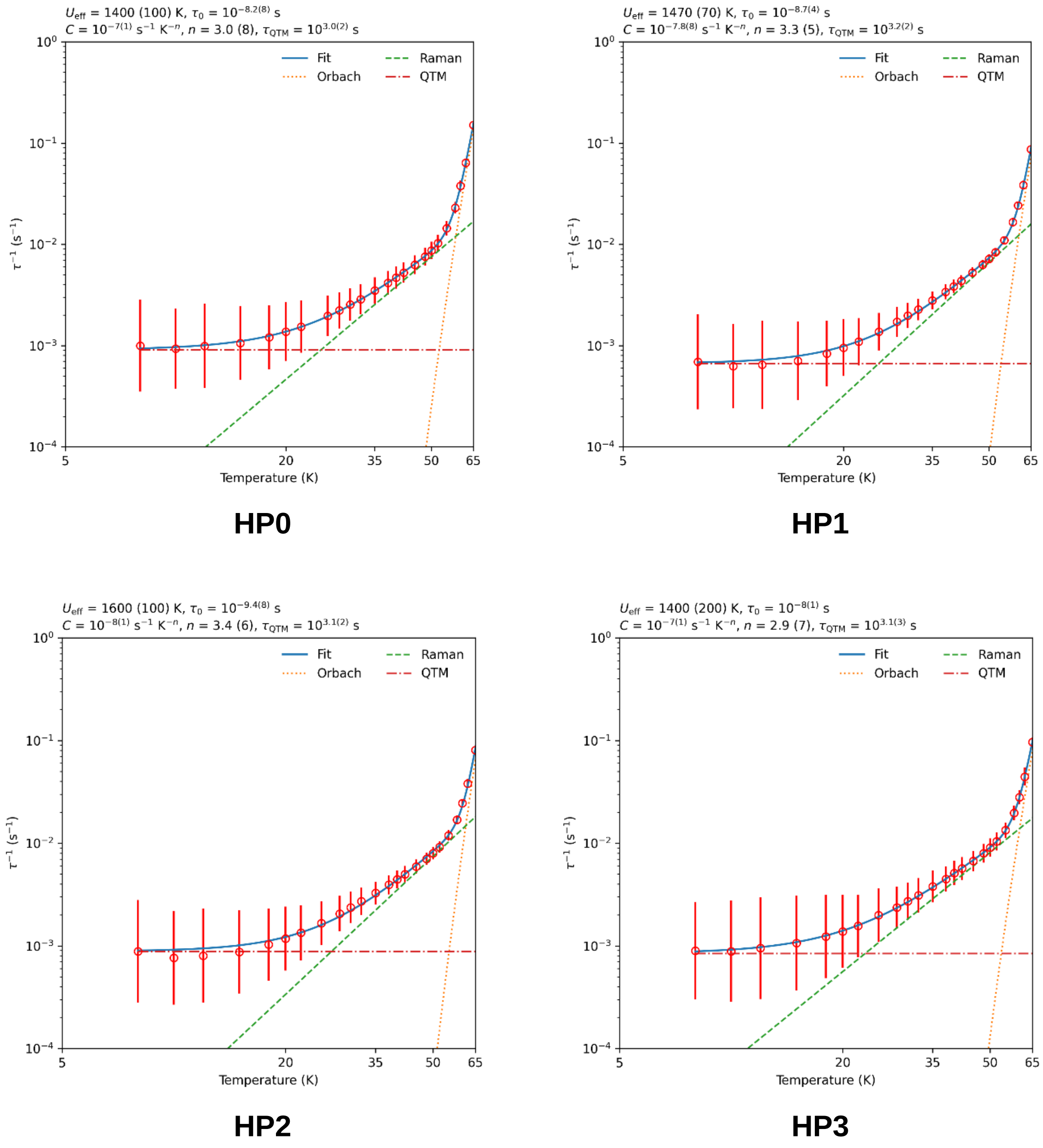

Figure S40. Relaxation profiles (τ vs T) and their best fits via CC-FIT2[3] for **1** at various pressure points.

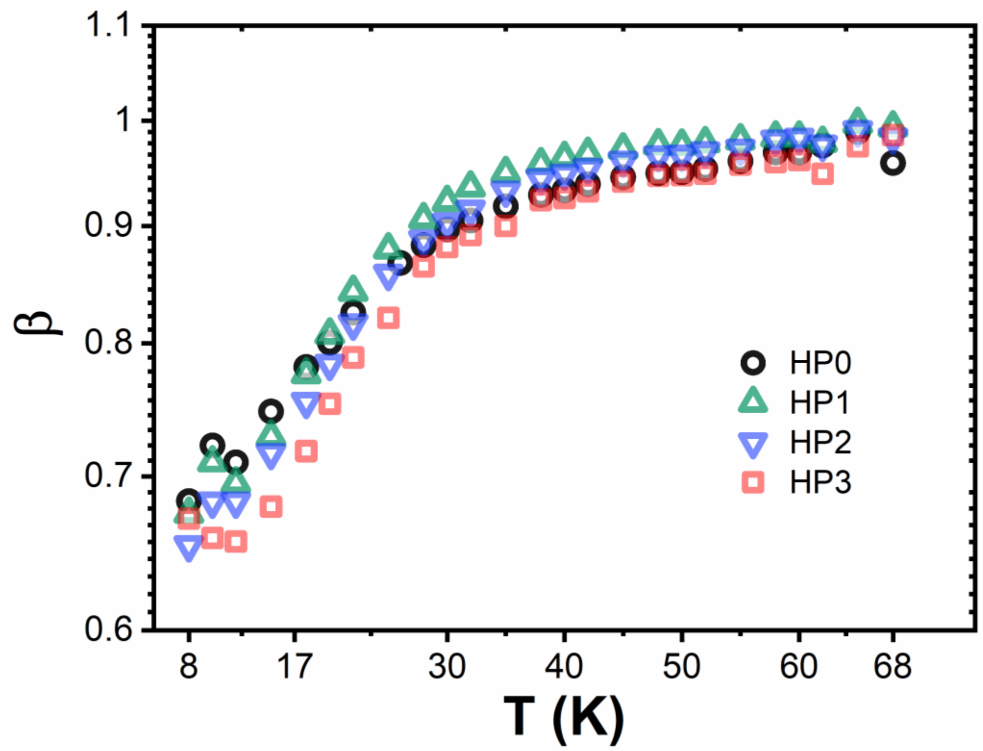

Figure S41. The distribution of magnetic relaxation times (β) at various pressure as a function of temperature for **1**.

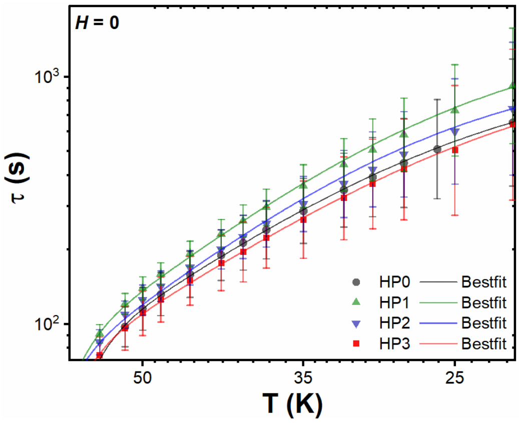

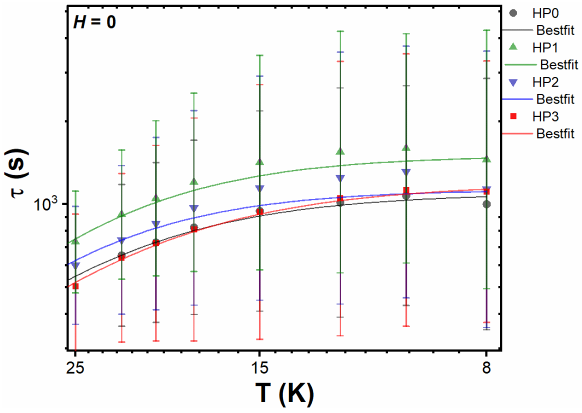

Figure S42. Zoomed-in sections of the relaxation profile from Figure 5 in the main text; *left*: Raman region, *right*: QTM region.

Table S19. Fitting results from the high-pressure magnetic decay measurements on **1** at ambient pressure (HP0). Magnetic decays measurements were fitted with a stretched exponential function (Equation S1) using CC-FIT2[3].

T **(K)**	M_0	M_{eq}	τ **(s)**	τ_{err}	**β**	β_{err}	τ_{ESD}^{up}	τ_{ESD}^{lw}
8	5.56E-04	4.30E-05	1.00E+03	4.52E-01	6.83E-01	3.44E-04	2.86E+03	3.51E+02
10	5.48E-04	3.96E-05	1.07E+03	7.37E-01	7.22E-01	5.27E-04	2.69E+03	4.30E+02
12	7.86E-04	3.63E-05	1.01E+03	4.87E-01	7.10E-01	3.83E-04	2.64E+03	3.90E+02
15	8.76E-04	3.23E-05	9.46E+02	4.20E-01	7.47E-01	3.77E-04	2.18E+03	4.10E+02
18	1.25E-03	3.32E-05	8.25E+02	3.17E-01	7.81E-01	3.53E-04	1.71E+03	3.99E+02
20	1.21E-03	2.81E-05	7.27E+02	2.07E-01	8.01E-01	2.81E-04	1.42E+03	3.73E+02
22	1.87E-03	2.98E-05	6.53E+02	1.76E-01	8.25E-01	2.80E-04	1.18E+03	3.62E+02
26	2.12E-03	1.97E-05	5.09E+02	1.18E-01	8.67E-01	2.62E-04	8.08E+02	3.21E+02
28	2.09E-03	2.61E-05	4.48E+02	1.01E-01	8.83E-01	2.63E-04	6.77E+02	2.96E+02
30	2.03E-03	1.99E-05	3.93E+02	1.04E-01	8.97E-01	3.18E-04	5.69E+02	2.72E+02
32	2.08E-03	2.36E-05	3.48E+02	7.32E-02	9.05E-01	2.57E-04	4.91E+02	2.47E+02
35	1.84E-03	2.10E-05	2.86E+02	5.33E-02	9.18E-01	2.35E-04	3.87E+02	2.12E+02
38	1.82E-03	2.04E-05	2.40E+02	4.66E-02	9.28E-01	2.53E-04	3.14E+02	1.84E+02
40	1.74E-03	1.73E-05	2.13E+02	4.11E-02	9.33E-01	2.52E-04	2.75E+02	1.65E+02
42	1.69E-03	1.74E-05	1.90E+02	4.09E-02	9.39E-01	2.87E-04	2.40E+02	1.50E+02
45	1.53E-03	-1.78E-05	1.59E+02	2.84E-02	9.45E-01	2.40E-04	1.97E+02	1.28E+02
48	1.41E-03	-2.24E-05	1.32E+02	2.59E-02	9.49E-01	2.66E-04	1.61E+02	1.08E+02
50	1.34E-03	-2.44E-05	1.15E+02	2.78E-02	9.50E-01	3.26E-04	1.40E+02	9.44E+01
52	1.28E-03	-2.63E-05	9.74E+01	2.10E-02	9.53E-01	2.90E-04	1.17E+02	8.08E+01
55	1.15E-03	-2.91E-05	6.96E+01	1.76E-02	9.60E-01	3.46E-04	8.18E+01	5.93E+01
58	1.05E-03	-4.35E-06	4.35E+01	1.73E-02	9.69E-01	5.58E-04	4.96E+01	3.82E+01
60	8.13E-04	-3.29E-05	2.65E+01	1.46E-02	9.69E-01	7.70E-04	3.02E+01	2.33E+01
62	6.23E-04	-3.47E-05	1.57E+01	1.08E-02	9.76E-01	9.81E-04	1.75E+01	1.41E+01
65	2.46E-04	-3.40E-05	6.64E+00	1.56E-02	9.89E-01	3.49E-03	7.00E+00	6.29E+00
68	9.07E-06	-3.30E-05	2.84E+00	8.09E-02	9.59E-01	4.35E-02	3.35E+00	2.40E+00

Table S20. Fitting results from high-pressure magnetic decay measurements on **1** at HP1. Magnetic decays measurements were fitted with a stretched exponential function (Equation S1) using CC-FIT2[3].

T (K)	M_0	M_{eq}	τ (s)	τ_{err}	β	$β_{err}$	τ_{ESD}^{up}	τ_{ESD}^{lw}
8	4.14E-05	1.45E+03	6.65E-01	6.74E-01	3.34E-04	4.27E+03	4.94E+02	4.14E-05
10	3.56E-05	1.60E+03	5.06E-01	7.10E-01	2.57E-04	4.15E+03	6.13E+02	-9.56E-05
12	3.35E-05	1.55E+03	6.68E-01	6.95E-01	3.14E-04	4.23E+03	5.65E+02	3.35E-05
15	2.84E-05	1.42E+03	4.57E-01	7.29E-01	2.51E-04	3.47E+03	5.78E+02	2.84E-05
18	2.61E-05	1.20E+03	2.38E-01	7.76E-01	1.80E-04	2.53E+03	5.71E+02	2.61E-05
20	2.49E-05	1.05E+03	1.37E-01	8.07E-01	1.31E-04	2.01E+03	5.50E+02	2.49E-05
22	2.39E-05	9.17E+02	1.05E-01	8.42E-01	1.24E-04	1.57E+03	5.36E+02	2.39E-05
25	2.22E-05	7.31E+02	5.36E-02	8.79E-01	8.56E-05	1.12E+03	4.78E+02	2.22E-05
28	2.05E-05	5.83E+02	3.39E-02	9.06E-01	7.20E-05	8.19E+02	4.15E+02	2.05E-05
30	1.95E-05	5.05E+02	3.03E-02	9.22E-01	7.66E-05	6.75E+02	3.78E+02	1.95E-05
32	1.89E-05	4.40E+02	2.52E-02	9.35E-01	7.47E-05	5.63E+02	3.44E+02	1.89E-05
35	1.78E-05	3.59E+02	2.16E-02	9.49E-01	8.10E-05	4.39E+02	2.94E+02	1.78E-05
38	1.59E-05	2.95E+02	2.04E-02	9.58E-01	9.46E-05	3.50E+02	2.48E+02	1.59E-05
40	1.43E-05	2.60E+02	1.85E-02	9.64E-01	9.87E-05	3.02E+02	2.24E+02	1.43E-05
42	1.31E-05	2.30E+02	1.85E-02	9.68E-01	1.12E-04	2.63E+02	2.01E+02	1.31E-05
45	1.06E-05	1.91E+02	1.90E-02	9.72E-01	1.41E-04	2.16E+02	1.70E+02	1.06E-05
48	8.32E-06	1.59E+02	1.56E-02	9.76E-01	1.40E-04	1.76E+02	1.43E+02	8.32E-06
50	6.61E-06	1.39E+02	1.62E-02	9.76E-01	1.66E-04	1.54E+02	1.25E+02	6.61E-06
52	4.62E-06	1.20E+02	1.44E-02	9.78E-01	1.71E-04	1.32E+02	1.09E+02	4.62E-06
55	2.63E-06	9.09E+01	1.45E-02	9.81E-01	2.29E-04	9.91E+01	8.33E+01	2.63E-06
58	1.61E-06	6.05E+01	1.25E-02	9.84E-01	2.98E-04	6.52E+01	5.61E+01	1.61E-06
60	-9.28E-07	4.14E+01	1.12E-02	9.83E-01	3.91E-04	4.47E+01	3.84E+01	-9.28E-07
62	-1.06E-06	2.59E+01	2.57E-02	9.78E-01	1.45E-03	2.85E+01	2.35E+01	-1.06E-06
65	-1.26E-06	1.15E+01	1.25E-02	9.97E-01	1.65E-03	1.17E+01	1.13E+01	-1.26E-06
68	-1.61E-06	4.66E+00	2.14E-02	9.93E-01	7.04E-03	4.83E+00	4.51E+00	-1.61E-06

Table S21. Fitting results from high-pressure magnetic decay measurements on **1** at HP2. Magnetic decays measurements were fitted with a stretched exponential function (Equation S1) using CC-FIT2[3].

T (K)	M_0	M_{eq}	τ (s)	τ_{err}	β	β_{err}	τ_{ESD}^{up}	τ_{ESD}^{lw}
8	4.89E-04	2.76E-06	1.13E+03	5.35E-01	6.53E-01	3.27E-04	3.59E+03	3.58E+02
10	4.45E-04	-8.81E-06	1.31E+03	4.43E-01	6.82E-01	2.57E-04	3.74E+03	4.58E+02
12	4.72E-04	2.80E-07	1.25E+03	3.92E-01	6.82E-01	2.40E-04	3.56E+03	4.36E+02
15	6.37E-04	7.91E-07	1.14E+03	2.93E-01	7.17E-01	2.12E-04	2.91E+03	4.49E+02
18	7.79E-04	-2.25E-06	9.70E+02	2.02E-01	7.54E-01	1.87E-04	2.19E+03	4.31E+02
20	9.25E-04	2.09E-05	8.51E+02	1.28E-01	7.84E-01	1.44E-04	1.75E+03	4.14E+02
22	1.05E-03	4.44E-07	7.43E+02	9.52E-02	8.16E-01	1.32E-04	1.38E+03	4.00E+02
25	1.32E-03	3.31E-06	6.01E+02	6.62E-02	8.58E-01	1.24E-04	9.82E+02	3.68E+02
28	1.51E-03	2.48E-05	4.86E+02	4.20E-02	8.88E-01	1.03E-04	7.23E+02	3.26E+02
30	1.55E-03	2.46E-05	4.22E+02	3.62E-02	9.03E-01	1.06E-04	5.99E+02	2.97E+02
32	1.59E-03	2.41E-05	3.69E+02	2.80E-02	9.15E-01	9.53E-05	5.04E+02	2.70E+02
35	1.69E-03	2.34E-05	3.06E+02	2.49E-02	9.32E-01	1.06E-04	3.95E+02	2.37E+02
38	1.63E-03	2.21E-05	2.54E+02	2.21E-02	9.44E-01	1.15E-04	3.16E+02	2.05E+02
40	1.57E-03	2.12E-05	2.25E+02	1.89E-02	9.48E-01	1.13E-04	2.76E+02	1.84E+02
42	1.51E-03	1.97E-05	2.00E+02	2.00E-02	9.54E-01	1.36E-04	2.41E+02	1.67E+02
45	1.41E-03	1.67E-05	1.69E+02	1.86E-02	9.61E-01	1.51E-04	1.99E+02	1.44E+02
48	1.30E-03	1.42E-05	1.42E+02	1.84E-02	9.66E-01	1.81E-04	1.64E+02	1.23E+02
50	1.25E-03	1.20E-05	1.25E+02	1.64E-02	9.66E-01	1.83E-04	1.44E+02	1.09E+02
52	1.19E-03	9.69E-06	1.09E+02	1.66E-02	9.70E-01	2.13E-04	1.24E+02	9.61E+01
55	1.13E-03	7.07E-06	8.43E+01	1.47E-02	9.72E-01	2.46E-04	9.48E+01	7.49E+01
58	1.01E-03	5.28E-06	5.91E+01	1.25E-02	9.81E-01	3.04E-04	6.43E+01	5.43E+01
60	9.15E-04	4.67E-06	4.09E+01	1.19E-02	9.83E-01	4.19E-04	4.42E+01	3.78E+01
62	7.61E-04	-1.78E-05	2.62E+01	2.44E-02	9.76E-01	1.35E-03	2.91E+01	2.37E+01
65	4.99E-04	2.94E-06	1.24E+01	1.14E-02	9.90E-01	1.35E-03	1.31E+01	1.19E+01
68	1.52E-04	1.45E-06	4.44E+00	2.31E-02	9.83E-01	7.87E-03	4.79E+00	4.12E+00

Table S22. Fitting results from high-pressure magnetic decay measurements on **1** at HP3. Magnetic decays measurements were fitted with a stretched exponential function (Equation S1) using CC-FIT2[3].

***T* (K)**	$\boldsymbol{M_0}$	$\boldsymbol{M_{eq}}$	**τ (s)**	$\boldsymbol{\tau_{err}}$	**β**	$\boldsymbol{\beta_{err}}$	$\boldsymbol{\tau_{ESD}^{up}}$	$\boldsymbol{\tau_{ESD}^{lw}}$
8	5.69E-04	5.67E-05	1.11E+03	2.91E-01	6.71E-01	1.89E-04	3.31E+03	3.74E+02
10	5.28E-04	4.90E-05	1.13E+03	3.48E-01	6.58E-01	2.19E-04	3.51E+03	3.62E+02
12	5.34E-04	4.36E-05	1.05E+03	3.20E-01	6.56E-01	2.16E-04	3.30E+03	3.34E+02
15	7.03E-04	2.97E-05	9.38E+02	4.18E-01	6.79E-01	3.14E-04	2.71E+03	3.24E+02
18	8.11E-04	2.64E-05	8.10E+02	1.80E-01	7.18E-01	1.84E-04	2.05E+03	3.19E+02
20	9.69E-04	2.56E-05	7.23E+02	1.17E-01	7.53E-01	1.46E-04	1.64E+03	3.20E+02
22	1.21E-03	2.59E-05	6.40E+02	9.10E-02	7.89E-01	1.37E-04	1.29E+03	3.17E+02
25	9.82E-04	2.45E-05	5.04E+02	5.99E-02	8.21E-01	1.23E-04	9.23E+02	2.75E+02
28	1.48E-03	2.29E-05	4.22E+02	5.40E-02	8.64E-01	1.45E-04	6.76E+02	2.64E+02
30	1.54E-03	2.23E-05	3.68E+02	4.92E-02	8.81E-01	1.57E-04	5.60E+02	2.42E+02
32	1.63E-03	2.14E-05	3.22E+02	3.67E-02	8.92E-01	1.36E-04	4.74E+02	2.19E+02
35	1.63E-03	1.94E-05	2.64E+02	3.12E-02	9.00E-01	1.44E-04	3.78E+02	1.84E+02
38	1.51E-03	1.80E-05	2.23E+02	3.43E-02	9.24E-01	1.96E-04	2.97E+02	1.68E+02
40	1.31E-03	1.64E-05	1.96E+02	2.92E-02	9.26E-01	1.91E-04	2.58E+02	1.48E+02
42	1.43E-03	1.51E-05	1.76E+02	2.73E-02	9.32E-01	2.01E-04	2.28E+02	1.36E+02
45	1.32E-03	1.19E-05	1.49E+02	2.79E-02	9.41E-01	2.48E-04	1.88E+02	1.19E+02
48	1.22E-03	8.67E-06	1.25E+02	2.59E-02	9.46E-01	2.77E-04	1.55E+02	1.02E+02
50	1.19E-03	7.38E-06	1.10E+02	2.29E-02	9.47E-01	2.79E-04	1.36E+02	8.98E+01
52	1.11E-03	6.15E-06	9.60E+01	2.15E-02	9.48E-01	3.01E-04	1.18E+02	7.84E+01
55	1.01E-03	6.44E-06	7.46E+01	2.29E-02	9.57E-01	4.20E-04	8.86E+01	6.28E+01
58	8.99E-04	4.93E-06	5.09E+01	1.70E-02	9.59E-01	4.59E-04	6.00E+01	4.32E+01
60	8.36E-04	8.09E-07	3.56E+01	1.36E-02	9.61E-01	5.25E-04	4.17E+01	3.04E+01
62	6.94E-04	5.24E-07	2.25E+01	1.51E-02	9.48E-01	9.00E-04	2.76E+01	1.84E+01
65	4.11E-04	-3.24E-07	1.04E+01	1.43E-02	9.75E-01	2.00E-03	1.16E+01	9.29E+00
68	1.45E-04	-3.95E-07	4.46E+00	2.34E-02	9.86E-01	7.98E-03	4.76E+00	4.17E+00

7. References

1 C. A. P. Goodwin, F. Ortu, D. Reta, N. F. Chilton and D. P. Mills, *Nature*, 2017, **548**, 439–442.

2 F.-S. Guo, B. M. Day, Y.-C. Chen, M.-L. Tong, A. Mansikkamäki and R. A. Layfield, *Angew. Chemie Int. Ed.*, 2017, **56**, 11445–11449.

3 F.-S. Guo, B. M. Day, Y.-C. Chen, M.-L. Tong, A. Mansikkamäki and R. A. Layfield, *Angew. Chemie Int. Ed.*, 2020, **59**, 18844.

4 J. B. Lambert, L. Lin and S. Keinan, *Org. Biomol. Chem.*, 2003, **1**, 2559–2565.

5 E. S. Stoyanov, I. V. Stoyanova and C. A. Reed, *Chem. Eur. J.*, 2008, **14**, 7880–7891.

6 K. Yokogawa, K. Murata, H. Yoshino and S. Aoyama, *Jpn. J. Appl. Phys.*, 2007, **46**, 3636–3639.

7 D. Jayatilaka, S. K. Wolff, D. J. Grimwood, J. J. McKinnon and M. A. Spackman, *Acta Crystallogr. Sect. A Found. Crystallogr.*, 2006, **62**, s90–s90.

8 G. Kresse and J. Hafner, *Phys. Rev. B*, 1993, **47**, 558–561.

9 G. Kresse and D. Joubert, *Phys. Rev. B*, 1999, **59**, 1758–1775.

10 G. Kresse and J. Furthmüller, *Comput. Mater. Sci.*, 1996, **6**, 15–50.

11 J. P. Perdew, K. Burke and M. Ernzerhof, *Phys. Rev. Lett.*, 1998, **80**, 891.

12 S. Grimme, J. Antony, S. Ehrlich and H. Krieg, *J. Chem. Phys.*, 2010, **132**, 154104.

13 J. P. Perdew, K. Burke and M. Ernzerhof, *Phys. Rev. Lett.*, 1996, **77**, 3865–3868.

14 K. Parlinski, Z. Q. Li and Y. Kawazoe, *Phys. Rev. Lett.*, 1997, **78**, 4063–4066.

15 A. Togo and I. Tanaka, *Scr. Mater.*, 2015, **108**, 1–5.

16 F. Neese, F. Wennmohs, U. Becker and C. Riplinger, *J. Chem. Phys.*, 2020, **152**, 224108.

17 B. O. Roos, P. R. Taylor and P. E. M. Sigbahn, *Chem. Phys.*, 1980, **48**, 157–173.

18 D. Aravena, F. Neese and D. A. Pantazis, *J. Chem. Theory Comput.*, 2016, **12**, 1148–1156.

19 F. Weigend, *J. Comput. Chem.*, 2008, **29**, 167–175.

20 F. Weigend and R. Ahlrichs, *Phys. Chem. Chem. Phys.*, 2005, **7**, 3297–3305.
